# Neoflavonoids as Inhibitors of HIV-1 Replication by Targeting the Tat and NF-κB Pathways

**DOI:** 10.3390/molecules22020321

**Published:** 2017-02-19

**Authors:** Dionisio A. Olmedo, José Luis López-Pérez, Esther del Olmo, Luis M. Bedoya, Rocío Sancho, José Alcamí, Eduardo Muñoz, Arturo San Feliciano, Mahabir P. Gupta

**Affiliations:** 1 Pharmaceutical Chemistry Area, Department of Pharmaceutical Sciences, University of Salamanca, Faculty of Pharmacy, CIETUS, IBSAL, Campus Miguel de Unamuno, 37007 Salamanca, Spain; lopez@usal.es (J.L.L.-P.); olmo@usal.es (E.d.O.); artsf@usal.es (A.S.F.); 2 Department of Cellular Biology, Physiology and Immunology, University of Córdoba, Faculty of Medicine Avda de Menendez Pidal s/n, 14004 Córdoba, Spain; Rocio.Sancho@cancer.org.uk (R.S.); fi1muble@uco.es (E.M.); 3 CIFLORPAN, Center for Pharmacognostic Research on Panamanian Flora, College of Pharmacy, University of Panama, P.O. Box 0824-00172 Panama, Panama; 4 National Centre of Microbiology, Institute Carlos III, Crt. Majadahonda a Pozuelo, 28220 Majadahonda, Madrid, Spain; lmbedoya@ucm.es (L.M.B.); ppalcami@isciii.es (J.A.); 5 Pharmacology Department, College of Pharmacy, Complutense University. Pz. Ramón Y Cajal s/n, 28040 Madrid, Spain

**Keywords:** neoflavonoids, 4-phenyl-chromen-one, AIDS, Tat protein, NF-κB inhibition, anti-HIV activity

## Abstract

Twenty-eight neoflavonoids have been prepared and evaluated in vitro against HIV-1. Antiviral activity was assessed on MT-2 cells infected with viral clones carrying the luciferase reporter gene. Inhibition of HIV transcription and Tat function were tested on cells stably transfected with the HIV-LTR and Tat protein. Seven 4-phenylchromen-2-one derivatives showed HIV transcriptional inhibitory activity but only the phenylchrome-2-one **10** inhibited NF-κB and displayed anti-Tat activity simultaneously. Compounds **10**, **14**, and **25**, inhibited HIV replication in both targets at concentrations <25 μM. The assays of these synthetic 4-phenylchromen-2-ones may aid in the investigation of some aspects of the anti-HIV activity of such compounds and could serve as a scaffold for designing better anti-HIV compounds, which may lead to a potential anti-HIV therapeutic drug.

## 1. Introduction

UNAIDS report in 2016 indicated that tuberculosis remains the leading cause of death among people living with HIV, accounting for around one in three AIDS-related deaths. In 2014, the percentage of identified HIV-positive tuberculosis patients who started or continued on ART reached 77% [1]. This relevant fact should promote further studies to take advantage of this therapeutic ambivalence and to evaluate the possibility of using 4-phenylchromene-2-ones for treating patients suffering from both AIDS and tuberculosis.

Human immunodeficiency virus (HIV) is the cause of acquired immunodeficiency syndrome (AIDS) which is one of the leading causes of 2.9% of mortality in the world [2]. Although modern antiretroviral therapy (ART) using a combination of anti-HIV drugs has been highly effective in suppressing HIV load and decreasing mortality in AIDS patients, the emergence of drug resistances in HIV and the toxicity of the therapies currently in use have made the continued search for novel anti-HIV drugs necessary [3,4]. On the other hand, failures in efforts to develop an effective vaccine against HIV-1 infection [5] have emphasized the importance of ART in treating HIV-1-infected patients. Therefore, medicinal chemists are interested in the development of novel anti-HIV agents that might be particularly effective in controlling strains of HIV that are resistant to the current drugs [6].

The HIV viral cycle can be divided into early and late stages. Early stages comprise several steps, from viral attachment on the cell surface to integration in the host genome. Late stages include the processes of HIV mRNA synthesis, protein expression and morphogenesis. Once integrated, HIV can remain in a latent state in resting lymphocytes or undergo active replication. Transition from latency to HIV expression occurs mainly when cells are activated and requires the concerted action of cellular transcription factors and regulatory HIV proteins [7,8]. Among the transcription factors involved in LTR transactivation, the HIV proximal enhancer contains three binding sites for SP1 transcription factor and two binding sites for NF-κB. The NF-κB/Rel family of transcription factors represents a major inducible regulatory element involved in HIV transcription [9]. Located downstream of the basal promoter TAR sequence is the RNA target for the viral protein Tat, which acts in concert with other cellular factors [10], to generate full-length RNA transcripts [11]. Furthermore, NF-κB and Tat cooperate in driving HIV replication from the state of latency. Therefore, inhibition of the activity of these critical proteins should result in an effective blocking of viral replication [12,13,14]. 

The neoflavonoids with anti-HIV activity possessing 4-phenylcoumarin skeleton have been obtained mainly from Calophyllaceae family that includes the genera: *Calophyllum* [15,16,17,18,19,20,21]; *Mammea* [21,22,23,24,25,26]; *Mesua* [27,28,29,30,31,32,33]; *Kielmeyera* [34,35,36,37,38]; and *Marila* [39], from which several 4-phenyl chromen-2-one derivatives have been isolated.

The discovery, structural modification and structure-activity relationships studies of natural neoflavonoids with anti-HIV activity:(+)-Inophyllum B [40], (+)-Inophyllum C [19], and Inophyllum P [40], and a number of their synthetic derivatives have been successfully obtained in this work. These 4-phenylcoumarins have been proposed as suppressors of LTR-dependent transcription, but the mechanism of action has not been fully characterized [41]. In addition, isomesuol and mesuol inhibit TNF-α-induced HIV-1-LTR transcriptional activity by targeting the nuclear factor-κB (NF-κB) pathway. Mesuol inhibited the phosphorylation and the transcriptional activity of the NF-κB p65 subunit in TNFα-stimulated cells [42]. Isodispar B and Disparinol A are HIV transcription inhibitors, which inhibit both, NF-κB and Tat targets, affecting the HIV replication by synergistic effect [43]. Synthetic 4-phenylchromen-2-ones have been also reported to show antimicrobial [30,44,45], anti-mycobacterial [46] and anti-inflammatory activities [47].

In a previous paper we reported the anti-HIV activity of natural 4-phenylcoumarins isolated from *Marila pluricostata*. They were structurally related to *Inophyllum* coumarins series, but with one prenyl and other cyclized group across the hydroxyl group at position C-7 [43]. Furthermore, these compounds showed moderate anti-mycobacterial activity. This relevant fact induced us to prepare new similar, but simpler, derivatives with the idea in mind to obtain compounds with activity against both HIV and tuberculosis, and also to increase the structural diversity. With that, a better structure-activity relationship could be established. In this paper we reported the preparation and the anti-HIV activity of several neoflavone derivatives that showed anti-mycobacterial activity [48].

## 2. Results and Discussion

### 2.1. Chemistry

**Scheme 1:**
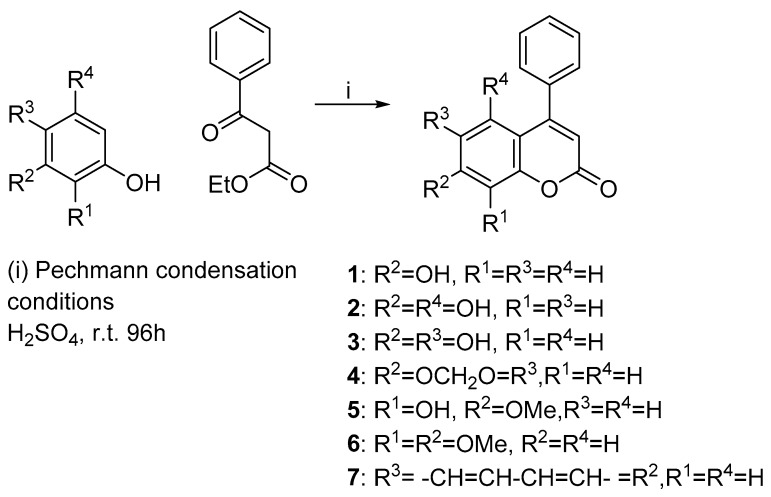
Preparation of 4-phenylcoumarins **1**–**7**.

The 4-phenylchromen-2-ones or neoflavones **1**–**7** have been obtained by the Peckman condensation between ethyl benzoylacetate and different phenol derivatives that is, resorcinol for **1**, phloroglucinol for **2**, hydroxyhydroquinone for **3**, 3,4-methylenedioxyphenol for **4**, 3-methoxycatechol for **5**, 2,3-dimethoxyphenol for **6** and β-naphthol for **7** in presence of concentred H_2_SO_4_ as condensing agent (Scheme 1). Some differences in the yield of the different compounds can be appreciated depending on the phenol derivative used (see the Experimental Section).

**Scheme 2:**
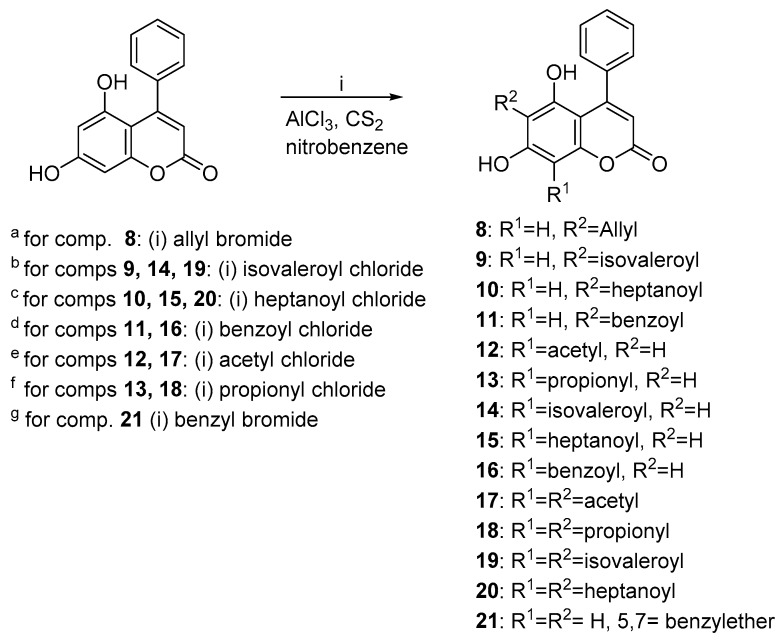
Preparation of neoflavones **8**–**21**.

Phloroglucinol, as the starting material (Scheme 1), was treated with ethyl benzoylacetate by the Pechmann-Duisberg reaction giving 5,7-dihydroxy-4-phenylcoumarin (**2**). Friedel-Craft acylation or benzoylation of compound **2**, by refluxing the phenol derivative with the corresponding acylchloride in a carbon disulfide/nitrobenzene mixture, and in the presence of aluminum trichloride, followed by Fries rearrangement provided mixtures of the 6-, 8-monoacylated and benzoylated neoflavones and 6,8-diacylated or dibenzoylated neoflavones [49,50,51,52]. Workup of the crude reaction products led to the isolation of neoflavones **8**–**21** whose spectroscopic properties were consistent with the structures shown in the Scheme 2. On the basis of detailed analysis of the H-H COSY (Correlation Spectroscopy), H-C HMQC (Heteronuclear Single Quantum Correlation), and H-C HMBC (Heteronuclear Multiple-Bond Correlation) 2D-NMR spectra, all of the compounds described were correctly characterized and their ^13^C-NMR data will be introduced in NAPROC-13 RMN spectroscopic database [53], for posterior online identification of natural products and their analogs and derivatives.

**Scheme 3:**
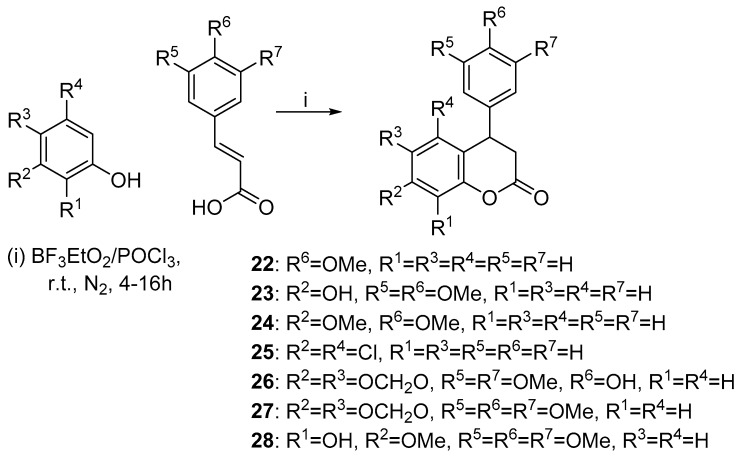
Preparation of neoflavones **22**–**28**.

Neoflavones **22**–**28** were obtained by the condensation of substituted cinnamic acids with the corresponding phenols, that is, phenol for **22**, resorcinol for **23** and **24**, 3,5-dichlorophenol for **25**, 3,4-methylenedioxyphenol for **26** and **27**, and 3-methoxybenzene-1,2-diol for **28**. The reaction occurred in the presence of milder Friedel-Crafts catalyst BF_3_–Et_2_O and POCl_3_ in 54%–75% yields (Scheme 3). Previous attempts towards condensation of substituted cinnamic acids with corresponding phenols for the preparation of 4-phenylchromen-2-ones **22**–**28** in the presence of concentrated HCl and HCl gas were unsuccessful. Additionally, some of these compounds were synthesized in low yields by microwave irradiation.

Some compounds prepared in this study have been described already in the literature (see the Experimental Section) and their structures were verified from an iterative search by ^13^C-NMR chemical shifts carried out within our NAPROC-13 RMN spectroscopic database [53], and later identified unambiguously. The rest of the prepared 4-phenylcoumarins were established on the basis of ^1^H- and ^13^C-NMR spectra. A combination of COSY, HMQC, and NOE experiments was utilized when necessary for a correct assignment of ^1^H and ^13^C chemical shifts.

The synthesized neoflavonoids **1**–**8** were evaluated in vitro against HIV-1 with the results shown in Table 1.

### 2.2. Evaluation of Antiviral Activity

The neoflavonoids synthesized (**1**–**28**) belong to three groups: simple 4-phenylchromen-2-ones, acyl-4-phenylchromen-2-ones and 3,4-dihydro-4-phenylchromen-2-ones, which have been evaluated in the anti HIV bioassay. The analysis of the results of inhibitory activity of NF-κB indicates that 4-phenylchromenones **9**, **10**, **13**, **14**, and **15** show a fair inhibitory activity at 50 μM. Regarding the specific HeLa-Tat-Luc assay results, compounds **9**, **13**, and **15** were nonspecific, whereas compounds **10** and **14** showed specificity. Furthermore compound **14** is only slightly toxic at 50 μM, but not toxic at 25 μM, and their activity as NF-κB and tat inhibitors is still strong (83.06% for NF-κB and 41.87% for Tat). Compound **14** turned out to be identical to the natural neoflavonoid Isodispar B previously isolated from *Marila pluricostata* [43]. Compound **10** NF-κB activity is also strong (70.53 at 25 μM) and it is nontoxic at 10 μM. Interestingly, the dichlorinated 3,4-dihydroflavonoid **25** showed specific anti-Tat activity, whereas all other 3,4-dihydroanalogs resulted inactive in this assay. It must be noted that simple structural differences within this series of 4-phenylchromen-2-ones and acyl 4-phenylchromen-2-ones, determine substantial changes in activity and selectivity. As an example, we could compare the specific NF-κB inhibitor 8-isovaleroyl-4-phenylchromen-2-one **14** and the specific Tat inhibitor 3,4-dihydro-5,7-dichloro-4-phenyl-chroman-2-one **25**, also a 4-phenylchromen related. The drastic difference in bioactivity for these compounds should be due either to the presence of an isovaleroyl or heptanoyl group in C-6 or C-8, which potentiates the activity in both targets. However, regarding the comparison of compounds **7** and **14**, it is worth noting that the presence of a benzene ring fused to the chromenone moiety, increases the anti-Tat activity, but the introduction 8-isovaleroyl or 6,8-diacetyl groups enhances the NF-κB inhibitory activity. Compound **10**, when compared to Disparinol A, showed higher anti-HIV potency than this natural neoflavonoid. The acyl derivatives of 4-phenylchromen-2-ones are the most potent dual-target inhibitors.

The assays of these 4-phenyl-chromen-2-one derivatives may aid in the investigation of some aspects of the anti-HIV activity of this kind of compound that inhibited the transcription and could serve as a scaffold for designing better anti-HIV compounds, which may lead to a potential HIV therapeutic drug.

## 3. Experimental Section

### 3.1. General Information

All of the reagents for synthesis were commercially available and either used without further purification or purified by standard methods prior to use. Melting points were determined on a Büchi 510-K melting point apparatus (Büchi Labortechnik AG, Flawil, Switzerland) and are uncorrected. IR spectra were recorded (KBr 1%) in a Nicolet Impact 410 spectrophotometer. ^1^H-, ^13^C-NMR, COSY, HMQC, and HMBC were recorded on Brüker AC 200 (200 MHz) and Brüker DRX 400 (400 MHz) instruments. Chemical shifts (δ) are expressed in parts per million (ppm) relative to the residual solvent peak, and coupling constants are reported in Hertz (Hz). All signals assigned to hydroxyl groups were exchangeable with D_2_O. Reaction progress was monitored using analytical thin-layer chromatography (TLC) on precoated Merck silica gel Kiesegel 60 F_254_ plates, and the spots were detected under UV light (254 nm). The flash chromatography was conducted using silica gel 230–400 mesh. For EIMS and HRFABMS analysis, a VG-TS250 mass spectrometer (70 eV) was used. Elementary analyses were obtained with a LECO CHNS-932 and were within ±0.4% of the theoretical values.

### 3.2. General Procedures I for the Synthesis of Compounds ***1***–***7***

To a mixture of appropriate phenol (2 mmol) and ethyl benzoyl acetate (2 mmol), concentrated H_2_SO_4_, (1 mL) was added and stirred at room temperature for four days; after which the mixture was poured over crushed ice and extracted with AcOEt, (50 mL × 5). Evaporation gave a brown solid which, after chromatography (silica gel, hexane/AcOEt 10:1→1:1), afforded the corresponding 4-phenylcoumarin (**1**–**7**) as a white solid (yield, 25%–30%).

*7-Hydroxy-4-phenyl-2H-chromen-2-one* (**1**). Yield 85%; A white solid; m.p. 232–234 °C (MeOH). The spectral data (^1^H-NMR) were quite comparable with the data reported in [54].

*5,7-Dihydroxy-4-phenyl-2H-chromen-2-one* (**2**). Yield 70%; A white solid; m.p. 227–229 °C (MeOH). The spectral data (^1^H-NMR) were quite comparable with the data reported in [52].

*6,7-Dihydroxy-4-phenyl-2H-chromen-2-one* (**3**). Yield 68%; A white solid; m.p. 230–232 °C (CHCl_3_/MeOH); IR (KBr): ν = 3437, 3414, 1686, 1617, 1562 cm^−1^; ^1^H-NMR (MeOD) δ 7.52 (m, 2H), 7.52 (m, 3H), 6.86 (s, 1H), 6.83 (s, 1H), 6.13 (s, 1H); ^13^C-NMR (MeOD) δ 104.0, 111.3, 111.9, 112.3, 129.4, 129.4, 129.9, 129.9, 130.6, 137.2, 144.4, 150.5, 152.0, 158.4, 164.1. MS (EI) *m*/*z*: 254 (M^+^ C_15_H_10_O_4_, 8), 252 (52), 224 (100), 152 (80), 139 (13).

*8-Phenyl-6H-[1,3]dioxolo[4,5-g]chromen-6-one* (**4**). Prepared from benzo[d][1,3]dioxol-5-ol (2 mol) as described in the general procedure I. Yield 65%; A white solid; m.p. 190–192 °C (CDCl_3_/MeOH). IR (KBr): ν = 1712, 1627, 1563, 1503 cm^−1^; ^1^H-NMR (CDCl3) δ 7.50 (m, 3H), 7.41 (m, 2H), 6.86 (s, 1H), 6.82 (s, 1H), 6.22 (s, 1H), 6.04 (s, 2H); ^13^C-NMR (CDCl_3_) δ 98.5, 102.3, 104.3, 112.1, 112.8, 128.2, 128.2, 128.8, 128.8, 129.6, 135.6, 144.8, 151.1, 151.2, 155.8, 161.1. MS (EI) *m*/*z*: 266 (M^+^ C_16_H_10_O_4_, 66), 265 (88), 238 (100), 152 (22).

*8-Hydroxy-7-methoxy-4-phenyl-2H-chromen-2-one* (**5**). Prepared as described in general procedure I from 3-methoxybenzene-1,2-diol (2 mmol), yield 65%; A white solid; m.p. 220–222 °C (CDCl_3_/MeOH); IR (KBr): ν = 3342, 2937, 2838, 1697, 1562 cm^‒1^; ^1^H-NMR (MeOD) δ 7.47 (m, 2H), 7.47 (m, 3H), 6.95 (d, *J* = 9.1 Hz, 1H), 6.87 (d, *J* = 9.1 Hz, 1H), 6.15 (s, 1H), 3.94 (s, 3H); ^13^C-NMR (MeOD) δ 56.7, 108.7, 112.1, 114.3, 118.4, 129.2, 129.2, 129.5, 129.5, 130.4, 136.5, 143.8, 151.7, 158.2, 162.7. MS (EI) *m*/*z*: 268 (M^+^ C_16_H_12_O_4_, 60), 265 (88), 225 (100), 152 (19), 141 (65).

*7,8-Dimethoxy-4-phenyl-2H-chromen-2-one* (**6**). This was prepared from 2,3-dimethoxyphenol (2 mol) as described in the general procedure I, yield 70%; A white solid; m.p. 175–177 °C (CDCl_3_); IR (KBr): ν = 2968, 2933, 2844, 1718, 1602, 1557 cm^−1^; ^1^H-NMR (CDCl_3_) δ 7.51 (m, 3H), 7.43 (m, 2H), 7.18 (d, *J* = 9.2 Hz, 1H), 6.83 (d, *J* = 9.2 Hz, 1H), 6.22 (s, 1H), 4.02 (s, 3H), 3.95 (s, 3H); ^13^C-NMR (CDCl_3_) δ 56.4, 61.5, 108.1, 112.3, 113.8, 122.2, 128.4, 128.4, 128.8, 128.8, 129.6, 135.6, 136.5, 148.4, 155.5, 155.9, 160.6. MS (EI) *m*/*z*: 282 (M^+^ C_17_H_14_O_4_, 100), 267 (8), 254 (19), 239 (42), 152 (24), 139 (53).

*4-Phenyl-2H-benzo[g]chromen-2-one* (**7**). Prepared as described in general procedure II from naphthalen-2-ol (2 mmol), yield 60%; A white solid; m.p. 211–213 (CDCl_3_); IR (KBr): ν = 3054, 1722, 1633, 1593, 1553 cm^‒1^; ^1^H-NMR (CDCl_3_) δ 8.57 (m, 1H), 7.83 (m, 1H), 7.52–7,62 (m, 9H), 6.44 (s, 1H); ^13^C-NMR (CDCl_3_) δ 114.1, 114.5, 122.3, 122.7, 123.3, 123.9, 127.1, 127.6, 128.5, 128.5, 128.9, 128.9, 128.9, 129.6, 134.8, 135.6, 151.4, 156.5, 160.8. MS (EI) *m*/*z*: 272 (M^+^ C_19_H_12_O_2_, 52), 245 (18), 244 (100), 215 (64), 189 (10), 139 (7).

### 3.3. General Procedures II: Synthesis of Compounds ***8***–***22***

Anhydrous aluminum trichloride (0.4 mmol) was added to a stirred suspension of compound **2** (0.1 mmol) in carbon disulfide (6 mL). Nitrobenzene (2 mL) was then added over 40 min, forming a homogeneous solution with evolution of HCl. The solution was heated under reflux for 30 min, appropriate acyl chloride (0.1 mmol) in nitrobenzene (1 mL) was added over 40 min before allowing it to cool with stirring. The mixture was poured onto ice/water and aqueous HCl and was extracted with ethyl acetate (25 mL, twice). Workup of the crude product by chromatography on silica gel led to the isolation of the different acyl derivatives products.

Following the general procedure II using allyl bromide and benzyl bromide the crude reaction product was chromatographed and eluted with hexane/EtOAc, to yield **8** and **21**.

*6-Allyl-5,7-dihydroxy-4-phenyl-2H-chromen-2-one* (**8**). Yield 41%; A white solid; m.p. 196–198 °C (CHCl_3_/MeOH); IR (KBr): ν = 3217, 1686, 1626, 1591 cm^‒1^; ^1^H-NMR (CDCl_3_) δ 7.24 (m, 2H), 7.22 (m, 3H), 6.55 (s, 1H), 6.36 (s, 1H), 5.70 (m, 1H), 4.82 (t, *J* = 1.8 Hz, 1H) 4.76 (dd, *J* = 1.2 Hz, 1H), 3.16 (d, *J* = 6.2 Hz, 2H); ^13^C-NMR (CDCl_3_) δ 26.7, 95.8, 110.8, 110.9, 114.5, 114.6, 127.2, 128.9, 129.4, 135.9, 137.3, 154.3, 154.4, 155.3, 160.1, 161.8. (EI) *m*/*z*: 294 (M^+^ C_18_H_14_O_4_, 88), 281 (100), 268 (24), 253(46), 139 (15).

*5,7-Dibenzyloxy-4-phenyl-2H-chromen-2-one* (**21**). Yield 45%; A white solid; m.p. 175–177 °C (CH_2_Cl_2_). IR (KBr): ν = 3089, 3059, 3031, 2927, 2865, 1720, 1611, 1597, 1432, 1337, 1159, 1111, 1064, 726 cm^‒1^. ^1^H-NMR (CDCl_3_) δ 7.38 (m, 3H), 7.20 (m, 5H), 7.18 (m, 10 H), 5.99 (s, 1H), 6.61 (d, *J* = 2.4 Hz, 1H), 6.41 (d, *J* = 2.4 Hz, 1H), 5.10 (s, 1 H), 4.72 (s, 1H). ^13^C-NMR (CDCl_3_) δ 70.5, 70.8, 94.37, 94.87, 103.8, 113.10, 127.03, 127.11, 127.51, 127.62, 127.73, 127.99, 128.25, 128.40, 128.80, 135.08, 135.78, 139.71, 156.74, 157.21, 157.35, 160.74, 162.32. (EI) *m*/*z*: 434 (M^+^ C_29_H_22_O_4_, 17), 343 (12), 181 (14), 139 (6), 114 (6), 92 (52), 91 (100).

Following the general procedure II using acetyl chloride, the crude reaction product was chromatographed and eluted with hexane/EtOAc, to yield **12** and **17**.

*8-Acetyl-5,7-dihydroxy-4-phenyl-2H-chromen-2-one* (**12**). Yield 25%; A white solid; m.p. 206–208 °C (Hex:AcOEt); IR (KBr): ν = 3224, 3083, 1689, 1627, 1592 cm^‒1^; ^1^H-NMR (CDCl_3_) δ 14.01 (s, 1H), 7.57 (m, 3H), 7.42 (m, 2H), 6.25 (s, 1H), 6.02 (s, 1H), 5.85 (s, 1H), 2.93 (s, 3H); ^13^C-NMR (CDCl_3_) δ 32.9, 99.3, 101.8, 103.7, 110.9, 126.8, 127.2, 127.9, 139.0, 157.9, 158.0, 159.9, 162.0, 167.8, 202.7. (EI) *m*/*z*, 272 (M^+^ C_19_H_12_O_2_, 52), 245 (18), 244 (100), 215(64), 189 (10). (EI) *m*/*z*: 296 (M^+^ C_17_H_12_O_5_, 18), 295 (100), 277 (22), 221(16), 165 (20), 139 (43).

*6,8-Diacetyl-5,7-dihydroxy-4-phenyl-2H-chromen-2-one* or (*1,1’-*(*5,7-dihydroxy-2-oxo-4-phenyl-2H-chromene-6,8-diyl-diethanone*) (**17**). Yield 52%; A white solid; m.p. 162–164 °C (Hex/AcOEt); IR (KBr): ν = 3467, 1733, 1717, 1593 cm^‒1^; ^1^H-NMR (CDCl_3_) δ 16.53 (s, 1H), 15.92 (s, 1H), 7.40 (m, 3H), 7.28 (m, 2H), 6.04 (s, 1H), 2.95 (s, 3H), 2.75 (s, 3H); ^13^C-NMR (CDCl_3_) δ 33.4, 33.5, 102.8, 106.3, 112.4, 126.9, 127.8, 128.5, 138.9, 156.5, 158.0, 161.5, 170.2, 172.2, 203.9, 205.5. (EI) *m*/*z*: 338 (M^+^ C_19_H_14_O_6_, 100), 323 (94), 313 (48), 295(90), 277 (26), 139 (29).

Following the general procedure II using propionyl chloride, the crude reaction product was chromatographed and eluted with hexane/EtOAc, to yield **13** and **18**.

*5,7-Dihydroxy-4-phenyl-8-propionyl-2H-chromen-2-one* (**13**). Yield 28%; A white solid; m.p. 216–218 °C (Hex:AcOEt); IR (KBr): ν = 3218, 3067, 1693, 1616, 1592 cm^−1^; ^1^H-NMR (MeOD) δ 7.37 (m, 2H), 7.37 (m, 3H), 6.18 (s, 1H), 5.99 (s, 1H), 3.36 (c, *J* = 7.3 Hz, 2H), 1.26 (t, *J* = 7.3 Hz, 3H); ^13^C-NMR (MeOD) δ 8.6, 38.5, 100.2, 111.5, 127.6, 127.9, 128.6, 140.1, 158.3, 160.9, 162.6, 168.7, 206.7. (EI) *m*/*z*: 310 (M^+^ C_18_H_14_O_5_, 36), 282 (18), 281 (100), 252 (8), 171 (7), 139 (8).

*5,7-Dihydroxy-4-phenyl-6,8-dipropionyl-2H-chromen-2-one* (**18**). Yield 49%; A white solid; m.p. 152–154 °C (Hex:AcOEt); IR (KBr): ν = 3468, 3437, 3067, 2978, 2937, 2876 cm^‒1^; ^1^H-NMR (CDCl_3_) δ 16.64 (s, 1H), 15.97 (s, 1H), 7.40 (m, 3H), 7.28 (m, 2H), 6.03 (s, 1H), 3.41 (q, *J* = 7.3 Hz, 2H), 3.19 (q, *J* = 6.7 Hz, 2H), 1.28 (t, *J* = 7.3 Hz, 3H), 1.15 (t, *J* = 6.7 Hz, 3H); ^13^C-NMR (CDCl_3_) δ 8.2, 8.5, 38.2, 38.2, 101.4, 102.4, 106.1, 112.2, 126.9, 127.8, 128.5, 139.0, 156.7, 161.2, 169.9, 170.0, 172.0, 207.3, 208.7. (EI) *m*/*z*: 366 (M^+^ C_21_H_18_O_6_, 36), 338 (44), 337 (100), 319 (27), 308 (28), 281 (40), 178 (9), 138 (14).

Following the general procedure II using 3-methylbutanoyl chloride, the crude reaction product was chromatographed and eluted with hexane/EtOAc, to yield **9**, **14**, and **19**.

*5,7-Dihydroxy-6-(3-methylbutanoyl)-4-phenyl-2H-chromen-2-one* (**9**). Yield 12%; A white solid; m.p. 206–208 °C (Hex:AcOEt); IR (KBr): ν = 3139, 3111, 2956, 2928, 2870, 1689, 1617, 1580 cm^‒1^; ^1^H-NMR (CDCl_3_) δ 15.79 (s, 1H), 11.39 (s, 1H), 7.52 (m, 3H), 7.39 (m, 2H), 6.63 (s, 1H), 5.98 (s, 1H), 2.90 (d, *J* = 6.3 Hz, 2H), 2.23 (m, 1H), 0.92 (d, *J* = 6.3 Hz, 3H), 0.92 (d, *J* = 6.3 Hz, 3H); ^13^C-NMR (CDCl_3_) δ 22.5, 22.5, 25.0, 53.0, 94.8, 101.4, 106.9, 111.3, 127.1, 127.4, 128.1, 139.0, 157.2, 159.6, 161.1, 163.7, 164.9, 207.3. (EI) *m*/*z*: 338 (M^+^ C_20_H_18_O_5_, 10), 282 (18), 281(100), 253 (3), 225(1), 171 (6), 139 (4).

*5,7-Dihydroxy-8-(3-methylbutanoyl)-4-phenyl-2H-chromen-2-one* (**14**). Yield 13%; A white solid; m.p. 210–212 °C (Hex:AcOEt); IR (KBr): ν = 3293, 2959, 2934, 2874, 2454, 1745, 1685, 1620, 1592 cm^‒1^; ^1^H-NMR (CDCl_3_) δ 14.14 (s, 1H), 7.55 (m, 3H), 7.43 (m, 2H), 6.25 (s, 1H), 6.01 (s, 1H), 6.00 (s, 1H), 3.17 (d, *J* = 6.3 Hz, 2H), 2.28 (m, 1H), 1.05 (d, *J* = 6.3 Hz, 3H), 1.05 (d, *J* = 6.3 Hz, 3H); ^13^C-NMR (CDCl_3_) δ 22.7, 22.7, 25.5, 53.6, 101.6, 101.8, 104.9, 112.0, 127.5, 129.6, 130.3, 136.5, 154.1, 155.7, 158.7, 159.7, 168.8, 205.9. (EI) *m*/*z*: 338 (M^+^ C_20_H_18_O_5_, 20), 323 (12), 281 (100), 254 (9), 171(6), 141 (6).

*5,7-Dihydroxy-6,8-bis(3-methylbutanoyl)-4-phenyl-2H-chromen-2-one or 1,1′-(5,7-dihydroxy-2-oxo-4-phenyl-2H-chromene-6,8-diyl)bis(3-methylbutan-1-one)* (**19**). Yield 50%; A white solid; m.p. 148–150 °C (Hex:AcOEt); IR (KBr): ν = 3469, 3435, 2959, 2931, 2871, 1756, 1618, 1597 cm^‒1^; ^1^H-NMR (CDCl_3_) δ 16.77 (s, 1H), 16.09 (s, 1H), 7.40 (m, 3H), 7.28 (m, 2H), 6.02 (s, 1H), 3.18 (d, *J* = 6.7 Hz, 2H), 3.02 (d, *J* = 6.6 Hz, 2H), 2.27 (m, 1H), 2.27 (m, 1H), 1.06 (d, *J* = 6.7 Hz, 3H), 1.06 (d, *J* = 6.7 Hz, 3H), 0.95 (d, *J* = 6.6 Hz, 3H), 0.95 (d, *J* = 6.6 Hz, 3H); ^13^C-NMR (CDCl_3_) δ 22.7, 22.7, 22.7, 22.7, 24.9, 25.8, 53.3, 53.3, 101.5, 102.6, 106.4, 112.2, 126.9, 127.8, 128.4, 139.1, 156.6, 158.0, 161.8, 170.3, 172.4, 206.6, 207.9. (EI) *m*/*z*: 422 (M^+^ C_25_H_26_O_6_, 34), 418 (25), 394 (36), 381 (43), 365 (100), 347 (36), 337(32), 281 (49), 171 (25), 139 (36).

Following the general procedure II using heptanoyl chloride, the crude reaction product was chromatographed and eluted with hexane/EtOAc, to yield **10**, **15**, and **20**.

*6-Heptanoyl-5,7-dihydroxy-4-phenyl-2H-chromen-2-one* (**10**). Yield 12%; A white solid; m.p. 210–212 °C (Hex:AcOEt); IR (KBr): ν = 3596, 3254, 3058, 2954, 2928, 2856, 1774, 1688, 1717, 1593 cm^‒1^; ^1^H-NMR (CDCl_3_) δ 14.03 (s, 1H), 10.11 (s, 1H), 7.44 (m, 3H), 7.35 (m, 2H), 6.99 (s, 1H), 5.99 (s, 1H), 3.10 (t, *J* = 6.7 Hz, 2H), 1.64 (m, 4H), 1.24 (m, 4H), 0.87 (t, *J* = 6.7 Hz, 3H); ^13^C-NMR (CDCl_3_) δ 14.1, 22.6, 24.4, 29.0, 31.7, 44.7, 95.9, 101.9, 107.1, 111.6, 127.4, 128.4, 128.9, 138.3, 157.7, 159.3, 161.8, 164.0, 164.8, 207.8. (EI) *m*/*z*: 366 (M^+^ C_22_H_22_O_5_, 32), 351 (14), 310 (30), (309 (100), 281 (82), 253(11), 171 (18), 139 (20).

*8-Heptanoyl-5,7-dihydroxy-4-phenyl-2H-chromen-2-one* (**15**). Yield 27%; A white solid; m.p. 212–214 °C (Hex:AcOEt); IR (KBr): ν = 3246, 2951, 2928, 2868, 2844 cm^−1^, 1685, 1637, 1591; ^1^H-NMR (CDCl_3_) δ 14.11 (s, 1H), 7.54 (m, 3H), 7.43 (m, 2H), 6.25 (s, 1H), 6.11 (s, 1H), 6.00 (s, 1H), 3.30 (t, *J* = 6.7 Hz, 2H), 1.73 (m, 4H), 1.33 (m, 4H), 0.89 (t, *J* = 6.7 Hz, 3H); ^13^C-NMR (CDCl_3_) δ 14.1, 22.6, 24.6, 29.0, 31.8, 44.9, 101.5, 105.0, 111.9, 127.5, 129.7, 130.2, 136.4, 154.0, 158.3, 158.6, 159.6, 168.7, 206.2. (EI) *m*/*z*: 366 (M^+^ C_22_H_22_O_5_, 44), 351 (31), 309 (91), 281 (100), 267 (3), 253 (18), 171 (23), 139 (39).

*6,8-Diheptanoyl-5,7-dihydroxy-4-phenyl-2H-chromen-2-one* or *1,1’-(5,7-dihydroxy-2-oxo-4-phenyl-2H-chromene-6,8-diyl)diheptan-1-one* (**20**). Yield 50%; A white solid; m.p. 164–166 °C (Hex:AcOEt); IR (KBr): ν = 3458, 34240, 2958, 2930, 2855, 1744, 1620, 1582 cm^‒1^; ^1^H-NMR (CDCl_3_) δ 16.69 (s, 1H), 16.02 (s, 1H), 7.40 (m, 3H), 7.28 (m, 2H), 6.02 (s, 1H), 3.33 (t, *J* = 7.3 Hz, 2H), 3.14 (t, *J* = 7.3 Hz, 2H), 1.76 (m, 4H), 1.64 (m, 4H), 1.32 (m, 4H), 1.32 (m, 4H), 0.90 (m, 3H), 0.90 (m, 3H); ^13^C-NMR (CDCl_3_) δ 14.0, 14.0, 22.6, 22.6, 24.3, 24.8, 29.0, 29.0, 31.6, 31.8, 44.8, 44.8, 101.4, 102.5, 106.2, 112.3, 127.0, 127.8, 128.5, 139.1, 156.6, 158.0, 161.2, 170.1, 172.2, 207.0, 208.4. (EI) *m*/*z*: 478 (M^+^ C_29_H_34_O_6_, 24), 421 (34), 393 (100), 323 (38), 293(52), 171 (20), 139 (14).

Following the general procedure II using benzoyl chloride, the crude reaction product was chromatographed and eluted with hexane/EtOAc, to **11** and **16**.

*6-Benzoyl-5,7-dihydroxy-4-phenyl-2H-chromen-2-one* (**11**). Yield 18%; A white solid; m.p. 245–247 °C (AcOEt). The spectral data (IR, ^1^H-NMR and ^13^C-NMR) were quite comparable with the data reported in [46,48].

*8-Benzoyl-5,7-dihydroxy-4-phenyl-2H-chromen-2-one* (**16**). Yield 34%; A white solid; m.p. 253–256 °C (AcOEt). The spectral data (IR, ^1^H-NMR and ^13^C-NMR) were quite comparable with the data reported in [50,51].

### 3.4. General Procedures III: Synthesis of Compounds ***22***–***28***

To a mixture of POCl_3_ (10 mmol) and BF_3_–Et_2_O (20 mmol) at 0 °C, appropriated cinnamic acid (5 mmol) was added and the reaction mixture stirred for 15 min at 0 °C. Phenol (5 mmol) was added to the above reaction mixture in small portions and stirring continued at 25–28 °C for 4–12 h. The reaction mixture was poured on to ice-water; sodium acetate (1 g) was added and the mixture was warmed on a water bath for 2 min. It was cooled, extracted with ethyl acetate (2 × 150 mL), washed with water (150 mL), dried, and the solvent removed under reduced pressure to obtain the crude product, which was purified by column chromatography using acetone–chloroform as eluent to afford pure 4-phenyldihydro-coumarins **22**–**28** in 60%–75% yields.

*4-(4-Methoxyphenyl)chroman-2-one* (**22**). This was prepared from *p*-methoxycinnamic acid and phenol using the general procedure III. Yield 62%; A white solid; m.p. 160–162 °C (CH_2_Cl_2_/MeOH); IR (KBr): ν = 2943, 2927, 2907, 2833, 1832, 1717, 1701, 1608 cm^‒1^; ^1^H-NMR (CDCl_3_) δ 7.31 (br d, *J* = 8.1 Hz, 1H), 7.12 (d, *J* = 8.2 Hz, 2H), 7.07 (br d, *J* = 8.2 Hz, 1H), 6.97 (m, 2H), 6.87 (d, *J* = 8.2 Hz, 3H), 4.30 (t, *J* = 7.3 Hz, 1H), 3.79 (s, 3H), 3.08 (dd, *J* = 7.3 Hz, 15.7 Hz, 1H), 2.96 (dd, *J* = 7.3, 15.7 Hz, 2H); ^13^C-NMR (CDCl_3_) δ 37.2, 40.0, 55.3, 114.5 (2C), 117.1, 124.7, 126.3, 128.3, 128.6 (3C), 132.2, 151.7, 159.0, 167.8. (EI) *m*/*z*: 254 (M^+^ C_16_H_14_O_3_, 65), 226 (14), 212 (15), 211 (72), 197 (22), 182 (15), 181 (100), 168 (13), 139 (12).

*4-(3,4-Dimethoxyphenyl)-7-hydroxychroman-2-one* (**23**). This was prepared from 3,4-dimethoxycinnamic acid and resorcinol as described in the general procedure III. Yield 67%; A white solid; m.p. 167–169 °C (CHCl_3_/MeOH); IR (KBr): ν = 3434, 2960, 2936, 1762, 1624, 1595 cm^‒1^; ^1^H-NMR (CDCl_3_) δ 6.83 (d, *J* = 7.7 Hz, 1H), 6.68 (dd, *J* = 7.7, 2.4 Hz, 1H), 6.68 (d, *J* = 2.4 Hz, 1H), 6.67 (d, *J* = 7.7 Hz, 1H), 6.66 (s, 1H), 6.59 (dd, *J* = 8.2, 2.4 Hz, 1H), 6.46 (br s, 1H), 4.21 (t, *J* =7.3 Hz, 1H), 3.85 (s, 3H), 3.81 (s, 3H), 3.06 (dd, *J* = 7.3 Hz, 14.9 Hz, 1H), 2.95 (dd, *J* = 7.3, 14.9 Hz, 1H); ^13^C-NMR (CDCl_3_) δ 37.5, 39.6, 55.9, 55.9, 104.2, 110.6, 111.6, 112.1, 117.5, 119.8, 129.1, 133.2, 148.3, 149.3, 152.1, 156.5, 168.7. (EI) *m*/*z*: 300 (M^+^ C_17_H_16_O_5_, 100), 269 (14), 257 (36), 243 (26), 227 (81), 190 (14), 139 (8).

*4-(4-Hydroxyphenyl)-7-methoxychroman-2-one* (**24**). This was prepared from *p*-coumaric acid and resorcinol as described in the general procedure III. Yield 68%; A white solid; m.p. 169–171 °C (CHCl_3_/MeOH); IR (KBr): ν = 3436, 2938, 2904, 2840, 1762, 1615 cm^‒1^; ^1^H-NMR (CDCl_3_) δ 6.98 (d, *J* = 8.3 Hz, 2H), 6.89 (d, *J* = 8.3 Hz, 1H), 6.77 (d, *J* = 8.3 Hz, 2H), 6.66 (d, *J* = 2.4 Hz, 1H), 6.64 (dd, *J* = 8.3, 2.4 Hz, 1H), 5.54 (br s, 1H), 4.22 (t, *J* = 6.8 Hz, 1H), 3.80 (s, 3H), 3.05 (dd, *J* = 6.8, 15.1Hz, 1H), 2.93 (dd, *J* = 6.8, 15.1 Hz, 1H); ^13^C-NMR (CDCl_3_) δ 37.4, 39.3, 55.6, 102.4, 110.7, 115.6, 115.6, 118.0, 128.7, 128.7, 128.8, 132.6, 152.3, 155.0, 159.9, 168.1. (EI) *m*/*z*: 270 (M^+^ C_16_H_14_O_4_, 38), 242 (17), 228 (10), 227 (100), 211 (40), 184 (15), 128 (18).

*5,7-Dichloro-4-phenylchroman-2-one* (**25**). This was prepared from cinnamic acid and 3,5-dichorophenol as described in the general procedure III. Yield 70%; A white solid; m.p. 230–232 °C (CHCl_3_); IR (KBr): ν = 3077, 1713, 1599, 1567 cm^‒1^; ^1^H-NMR (CDCl_3_) δ 7.27 (m, 3H), 7.22 (d, *J* = 1.9 Hz, 1H), 7.11 (d, *J* = 1.9 Hz, 1H), 7.06 (m, 2H), 4.64 (dd, *J* = 5.4, 3.4 Hz, 1H), 3.10 (dd, *J* = 3.4, 12.2 Hz, 1H), 3.04 (dd, *J* = 5.4, 12.2 Hz, 1H); ^13^C-NMR (CDCl_3_) δ 36.7, 38.5, 116.5, 122.2, 125.6, 126.7, 126.7, 127.9, 129.2, 129.2, 134.5, 138.9, 152.9, 165.7. (EI) *m*/*z*: 293 (M^+^ C_15_H_10_O_2_Cl_2_, 36), 291 (59), 276 (23), 274 (36), 256 (13), 251 (64), 249 (100), 215 (19), 152 (33).

*8-(4-Hydroxy-3,5-dimethoxyphenyl)-7,8-dihydro-[1,3]dioxolo[4,5-g]chromen-6-one* (**26**). This was prepared from 4-hydroxy-3,5-dimethoxycinnamic acid and sesamol as described in the general procedure III. Yield 69%; A white solid; m.p. 162–164 °C (eter); IR (KBr): ν = 3458, 3023, 3007, 2956, 2930, 2913, 2836, 1742, 1627, 1609 cm^‒1^; ^1^H-NMR (CDCl_3_) δ 6.65 (s, 1H), 6.41 (s, 1H), 6.36 (s, 2H), 5.96 (s, 2H), 5.52 (br s, 1H), 4.13 (t, *J* =7.3 Hz, 1H), 3.84 (s, 3H), 3.84 (s, 3H), 3.09 (dd, *J* = 7.3, 15.8 Hz, 1H), 2.92 (dd, *J* = 7.3, 15.8 Hz, 1H); ^13^C-NMR (CDCl_3_) δ 37.2, 40.8, 56.4, 56.4, 99.1, 101.7, 104.2, 104.2, 107.2, 118.2, 131.5, 134.2, 144.5, 146.1, 147.5, 147.5, 147.5, 167.8. (EI) *m*/*z*: 344 (M^+^ C_18_H_16_O_7_, 100), 326 (13), 295 (14), 271 (82), 256 (21), 167 (37), 133 (12).

*8-(3,4,5-Trimethoxyphenyl)-7,8-dihydro-[1,3]dioxolo[4,5-g]chromen-6-one* (**27**). This was prepared from 3,4,5-trimethoxycinnamic acid and sesamol as described in the general procedure III. Yield 61%; A white solid; m.p. 162–164 °C (diethyl ether); IR (KBr): ν = 3023, 3007, 2956, 2930, 2913, 2836, 1742, 1627, 1609, 1584 cm^‒1^; ^1^H-NMR (CDCl_3_) δ 6.66 (s, 1H), 6.43 (s, 1H), 6.35 (s, 2H), 5.96 (s, 2H), 4.15 (t, *J* = 6.8 Hz, 1H), 3.83 (s, 3H), 3.81 (s, 6H), 3.05 (dd, *J* = 6.8, 16.1 Hz, 1H), 2.92 (dd, *J* = 6.8, 16.1 Hz, 1H); ^13^C-NMR (CDCl_3_) δ 37.1, 41.0, 56.1, 56.1, 60.8, 99.2, 101.8, 104.4, 104.5, 107.2, 117.8, 136.1, 136.1, 137.5, 144.5, 146.1, 147.6, 153.7, 167.6. (EI) *m*/*z*: 358 (M^+^ C_19_H_18_O_7_, 100), 325 (29), 315 (21), 285 (83), 241 (27), 215 (35), 181 (81), 133 (38).

*8-Hydroxy-7-methoxy-4-(3,4,5-trimethoxyphenyl)chroman-2-one* (**28**). This was prepared from 3,4,5-trimethoxycinnamic acid and 3-methoxybenzene-1,2-diol as described in the general procedure III. Yield 60%; A white solid; m.p. 164–166 °C (CHCl_3_/MeOH); IR (KBr): ν = 3309, 3001, 2951, 2841, 1759, 1629, 1588 cm^‒1^; ^1^H-NMR (CDCl_3_) δ 6.64 (d, *J* = 8.5 Hz, 1H), 6.48 (d, *J* = 8.5 Hz, 1H), 6.37 (s, 2H), 5.73 (s, 1H), 4.24 (t, *J* = 7.3 Hz, 1H), 3.90 (s, 3H), 3.83 (s, 3H), 3.80 (s, 6H), 3.09 (dd, *J* = 7.3, 14.1 Hz, 1H), 2.97 (dd, *J* = 7.3, 14.1 Hz, 1H); ^13^C-NMR (CDCl_3_) δ 37.2, 40.8, 56.4, 56.4, 56.4, 60.8, 104.6, 104.6, 107.0, 117.9, 119.2, 133.8, 136.2, 137.4, 139.4, 147.2, 153.6, 153.6, 166.8. (EI) *m*/*z*: 360 (M^+^ C_19_H_20_O_7_, 100), 345 (22), 327 (35), 317 (19), 287 (99), 272 (24), 217 (17), 181 (31), 173 (22).

### 3.5. Antiviral Activity Assays

The anti-HIV activity of these neoflavonoids on Tat and NF-κB functions has been evaluated. To this aim, we have used two stably transfected cell lines. The previously described 5.1 cell line [55] is a Jurkat-derived clone stably transfected with a plasmid containing the luciferase gene under the control of HIV-LTR. In this cell clone, activation with TNFα induces NF-κB activation and subsequent HIV-1 expression. We have also analysed the anti-HIV activity in HeLa-Tat-Luc cells, in which the HIV-1 LTR is directly activated by the HIV-1 Tat protein. A compound was considered active in one assay if it inhibited the target function by more than 50% (NF-κB) or 30% (Tat) at either 25 or 50 μM concentration. The active compounds were submitted for further evaluation through a HeLa-Tet-ON assay, as previously described [56]. In the Hela-Tet-ON cells the luciferase expression is under control of an artificial promoter that can be activated by tetracycline. Therefore, compounds that inhibit tetracycline-induced luciferase activity were considered non-specific for luciferase-based anti-HIV assays.

Cell viability was evaluated in non-infected treated cultures following the same protocol as in the recombinant virus assay and measuring cell toxicity with a classical MTT assay. IC_50_ were calculated using GraphPad Prism software (non-linear regression, log (inhibitor) vs. response).

## 4. Conclusions

A series of twenty-eight neoflavonoids have been synthesized and evaluated against HIV-1 in vitro. Ten 4-phenylchromen-2-one derivatives displayed HIV specific transcriptional inhibition and five displayed nonspecific mechanisms of action. The heptanoylchromen-one **10** was the more potent Tat antagonist, while compound **14** showed high inhibition of the NF-κB pathway. A preliminary SAR analysis established that the presence of the acyl group is essential for the anti HIV in both targets.

## Figures and Tables

**Table 1:** Anti-HIV Activity of neoflavonoids.

Compound	NF-κB (5.1 LTR)		Hela-Tat-luc		Specificity (HeLa-Tet-On-Luc)	Toxicity MT2 (%)
	25 µM	50 µM	25 µM	50 µM	50 µM	50 µM
**1**	NT	−11.28	NT	27.88	NT	18.40
**2**	NT	−4.10	NT	−7.43	NT	8.78
**3**	NT	34.27	NT	6.59	NT	2.22
**4**	7.30	21.66	5.58	34.64	S	3.55
**5**	22.98	23.83	−2.63	44.60	S	11.30
**6**	NT	19.12	NT	16.25	NT	2.88
**7**	NT	51.71	NT	94.69	U	4.00
**8**	NT	9.25	NT	21.74	NT	8.33
**9**	NT	68.74	NT	80.84	U	NT
**10**	70.53	68.19	NT	83.32	S	<10
**11**	NT	37.81	NT	26.63	S	NT
**12**	NT	20.40	NT	5.81	S	NT
**13**	NT	67.29	NT	66.72	U	NT
**14**	83.06	86.60	41.87	69.32	S	17.02
**15**	NT	79.41	NT	80.37	U	NT
**16**	NT	−17.05	NT	20.87	S	NT
**17**	NT	83.70	NT	44.93	U	NT
**18**	NT	35.05	NT	30.20	S	NT
**19**	59.86	66.04	NT	12.99	S	NT
**20**	NT	10.70	NT	22.30	S	NT
**22**	NT	15.20	NT	6.06	NT	1.61
**23**	NT	11.94	NT	−34.54	NT	2.55
**24**	36.95	43.21	NT	−18.03	NT	2.00
**25**	35.20	53.99	57.46	72.27	S	3.50
**26**	NT	13.37	NT	−28.08	NT	2.59
**27**	NT	20.48	NT	15.08	NT	4.73
**28**	NT	5.67	NT	−65.28	NT	6.13
Mesuol	71.00	77.90	NT	71.30	S	>4 μM

S = Specific activity; U = Unspecific mode of action; NT = Not tested.

## Supplementary Materials

The following are available online at http://www.mdpi.com/1420-3049/22/2/321/s1, Figure S1: Spectroscopic data of neoflavonoid derivatives **3**–**10**, **12**–**15**, **17**–**28**.
