# Supplementary material for: Neoflavonoids as Inhibitors of HIV-1 Replication by Targeting the Tat and NF-κB Pathways
Source: Molecules. 2017 Feb 19;22(2):321. doi: 10.3390/molecules22020321 (PMC6155902; doi:10.3390/molecules22020321)
Supplement: Supplementary file 1 [file molecules-22-00321-s001.pdf]

INFORMATION SUPPLEMENTARY

Neoflavonoids as Inhibitors of HIV-1 Replication by targeting the Tat and NF-*k*B Pathways

Dionisio A. Olmedo [1*], José Luis López-Pérez [1], Esther del Olmo [1], Luis M. Bedoya [4, 5], Rocío Sancho [2], José Alcamí [4], Eduardo Muñoz [2], Arturo San Feliciano [1] and Mahabir P. Gupta[3*].

FIGURE S1: SPECTRA OF THE NEOFLAVONOIDS DERIVATIVES 3-10, 12-15, 17-28

*S1: 6,7-dihydroxy-4-phenyl-2H-chromen-2-one (***3***)*
S2: 8-phenyl-6H-[1,3]dioxolo[4,5-g]chromen-6-one (**4**)
S3: 8-hydroxy-7-methoxy-4-phenyl-2H-chromen-2-one (**5**)

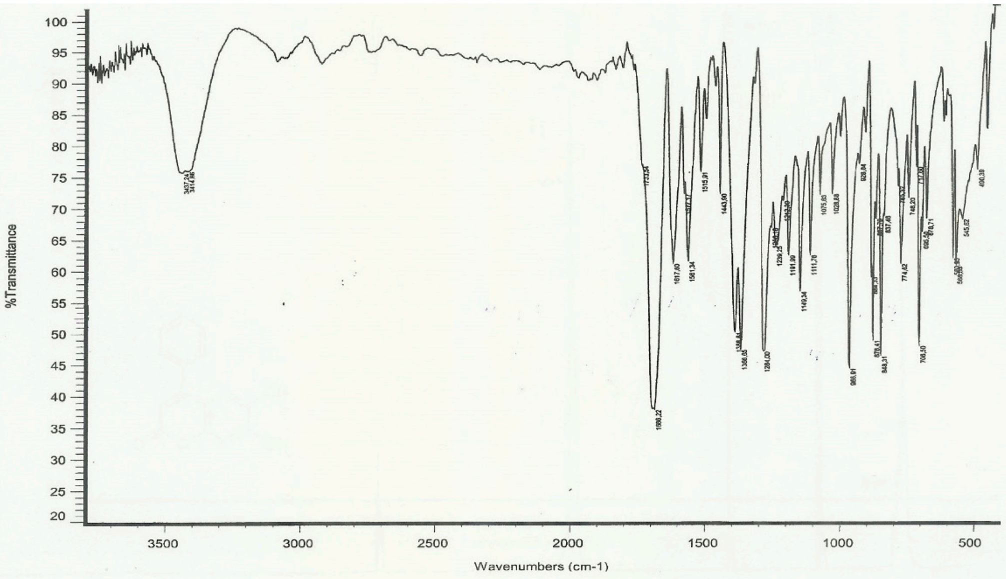

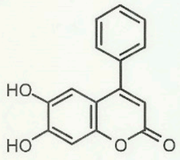

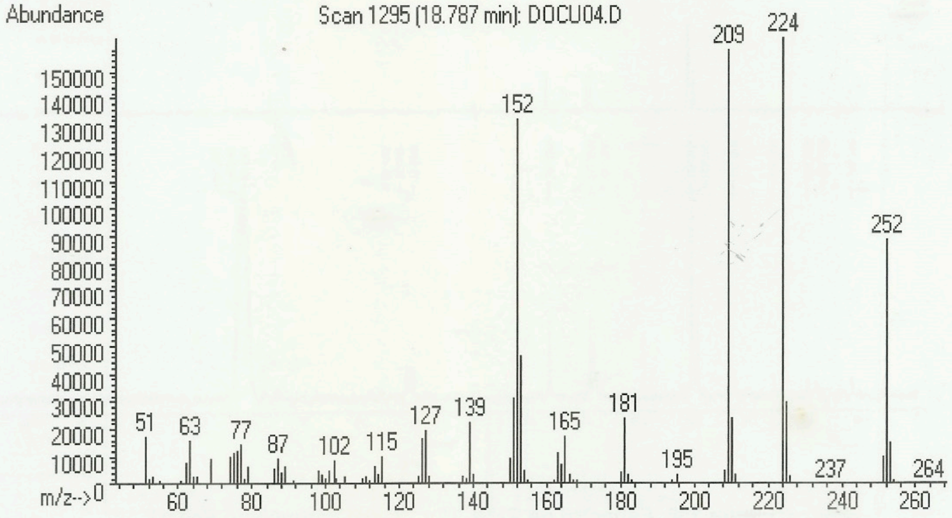

6,7-dihydroxy-4-phenyl-2H-chromen-2-one (**3**)

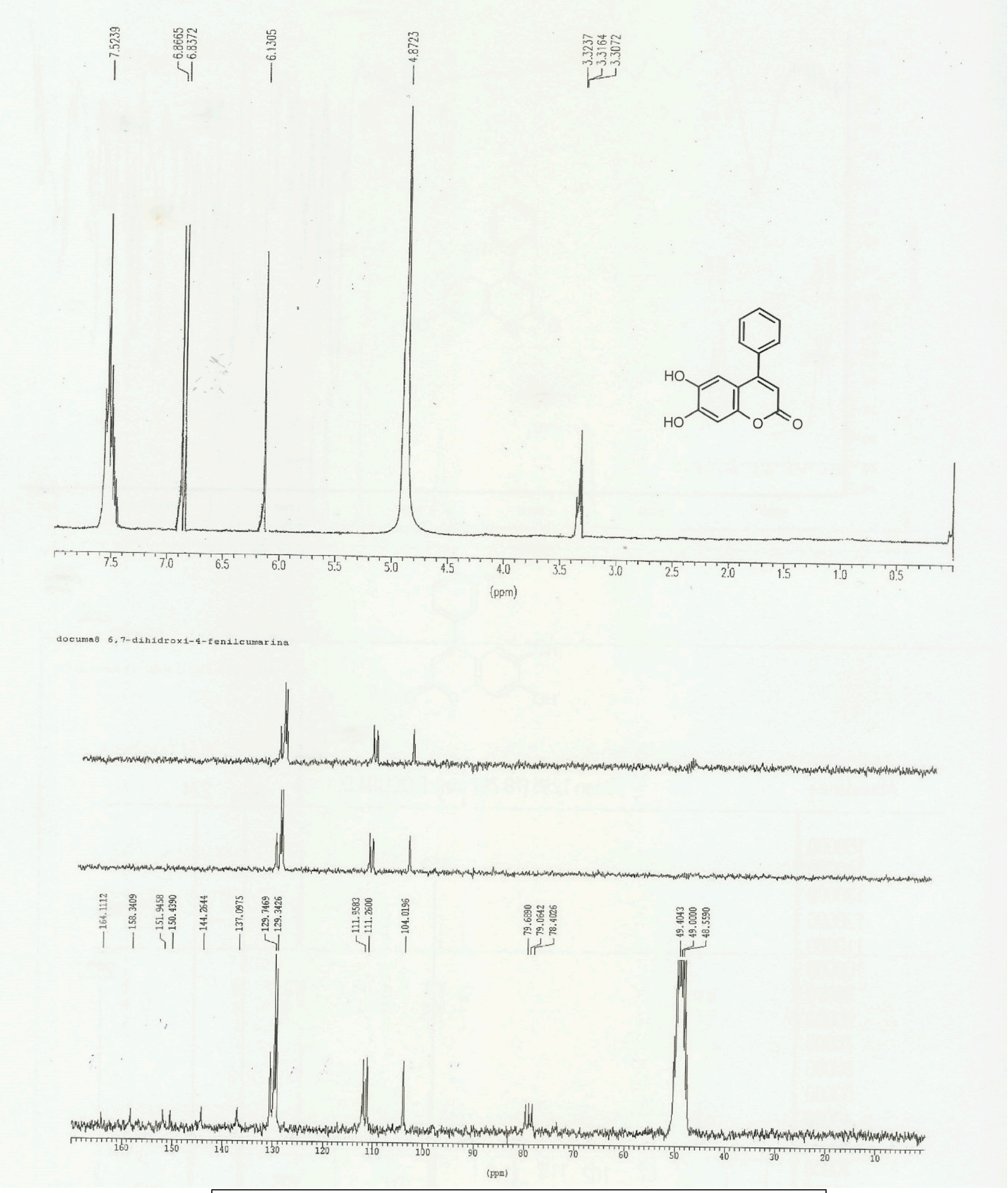

6,7-dihydroxy-4-phenyl-2H-chromen-2-one (**3**)

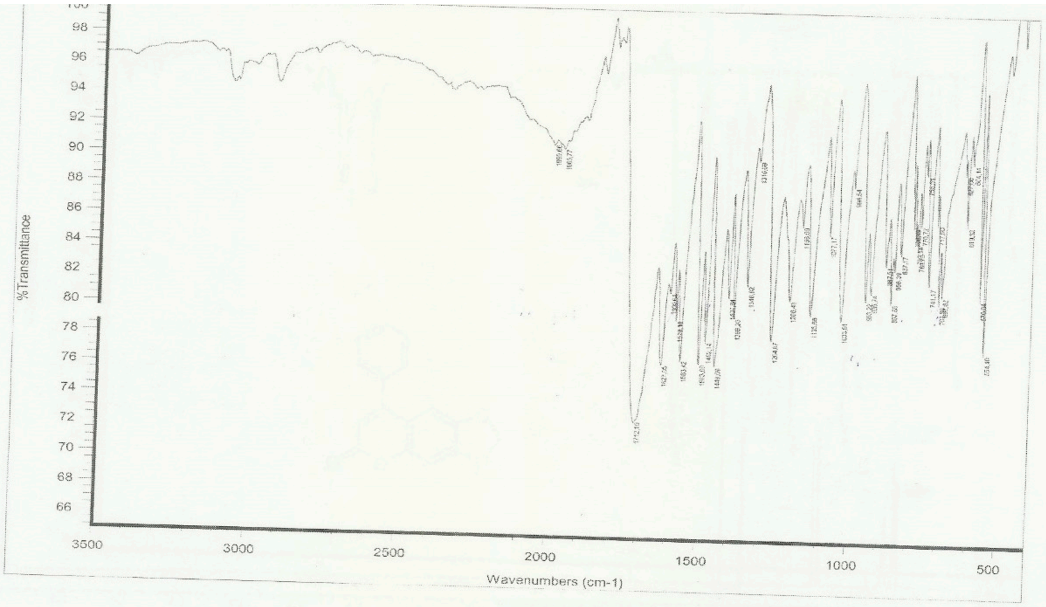

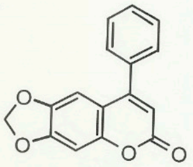

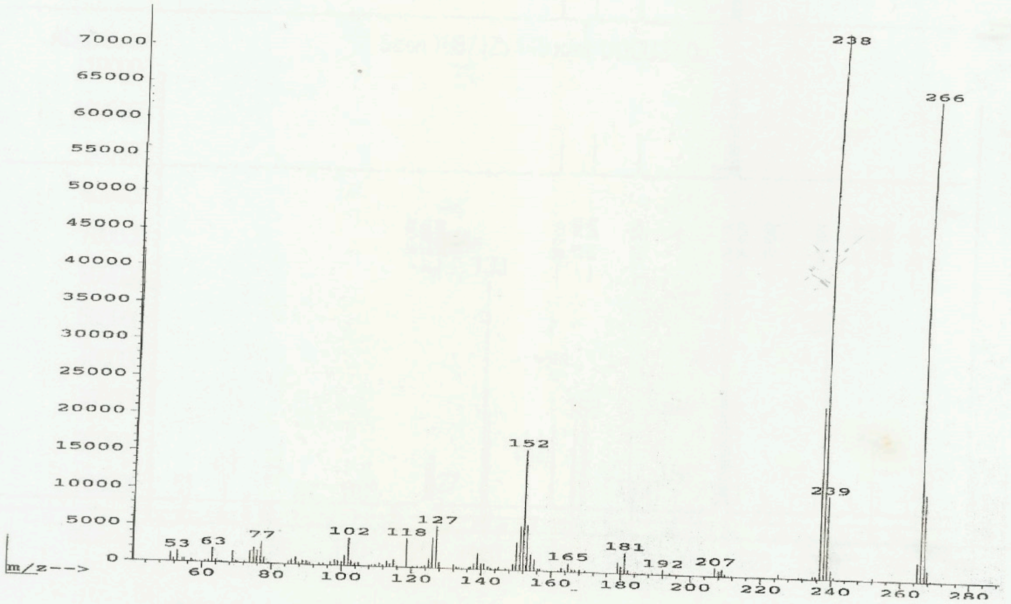

8-phenyl-6H-[1,3]dioxolo[4,5-g]chromen-6-one (**4**)

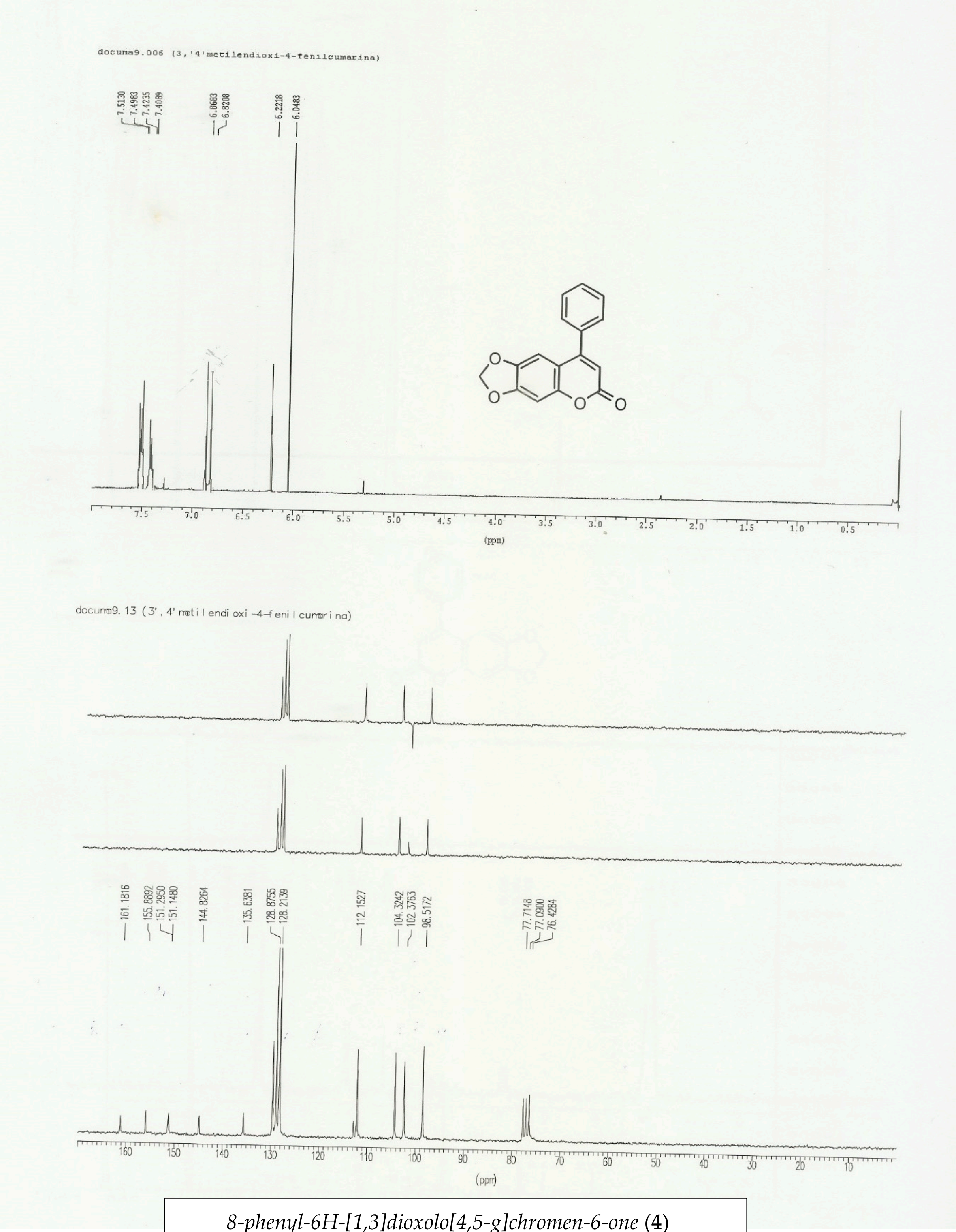

8-phenyl-6H-[1,3]dioxolo[4,5-g]chromen-6-one (**4**)

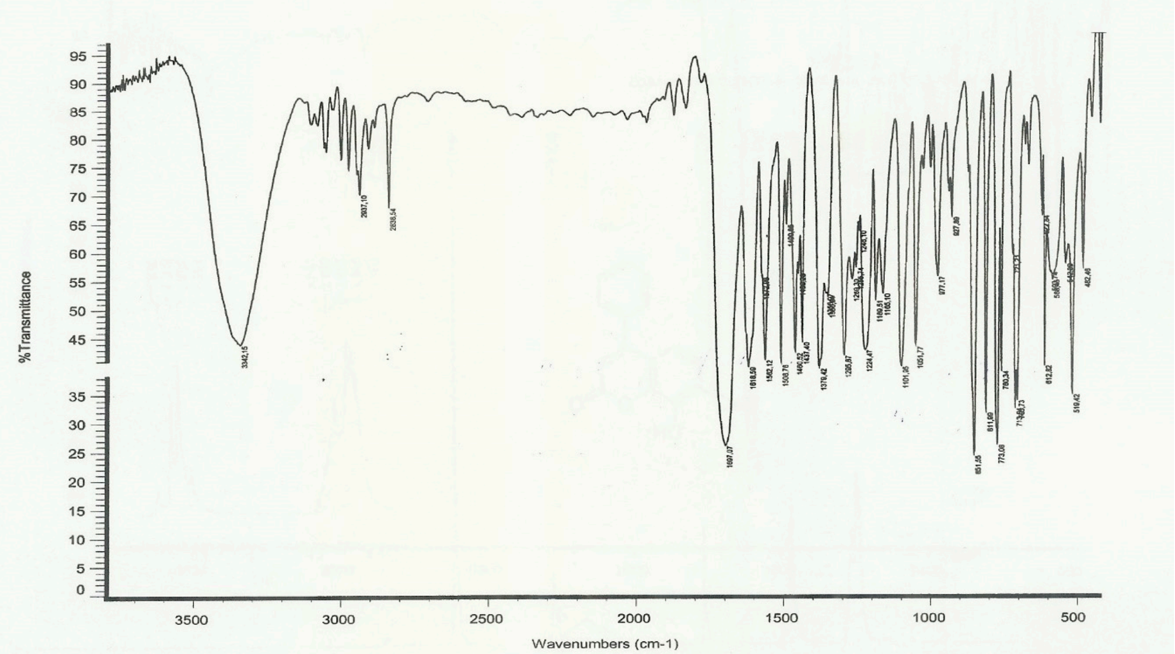

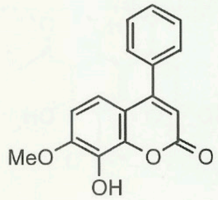

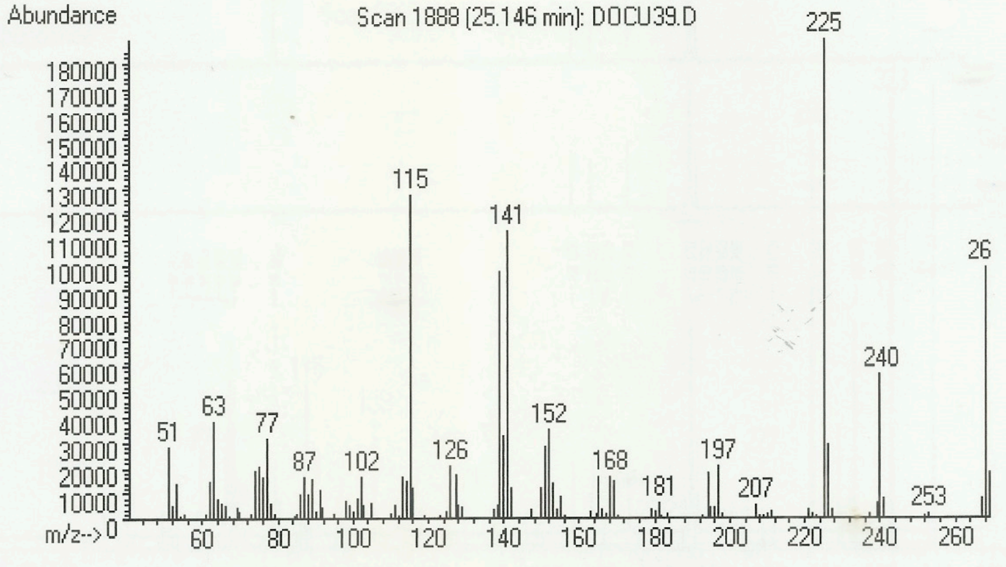

8-hydroxy-7-methoxy-4-phenyl-2H-chromen-2-one (**5**)

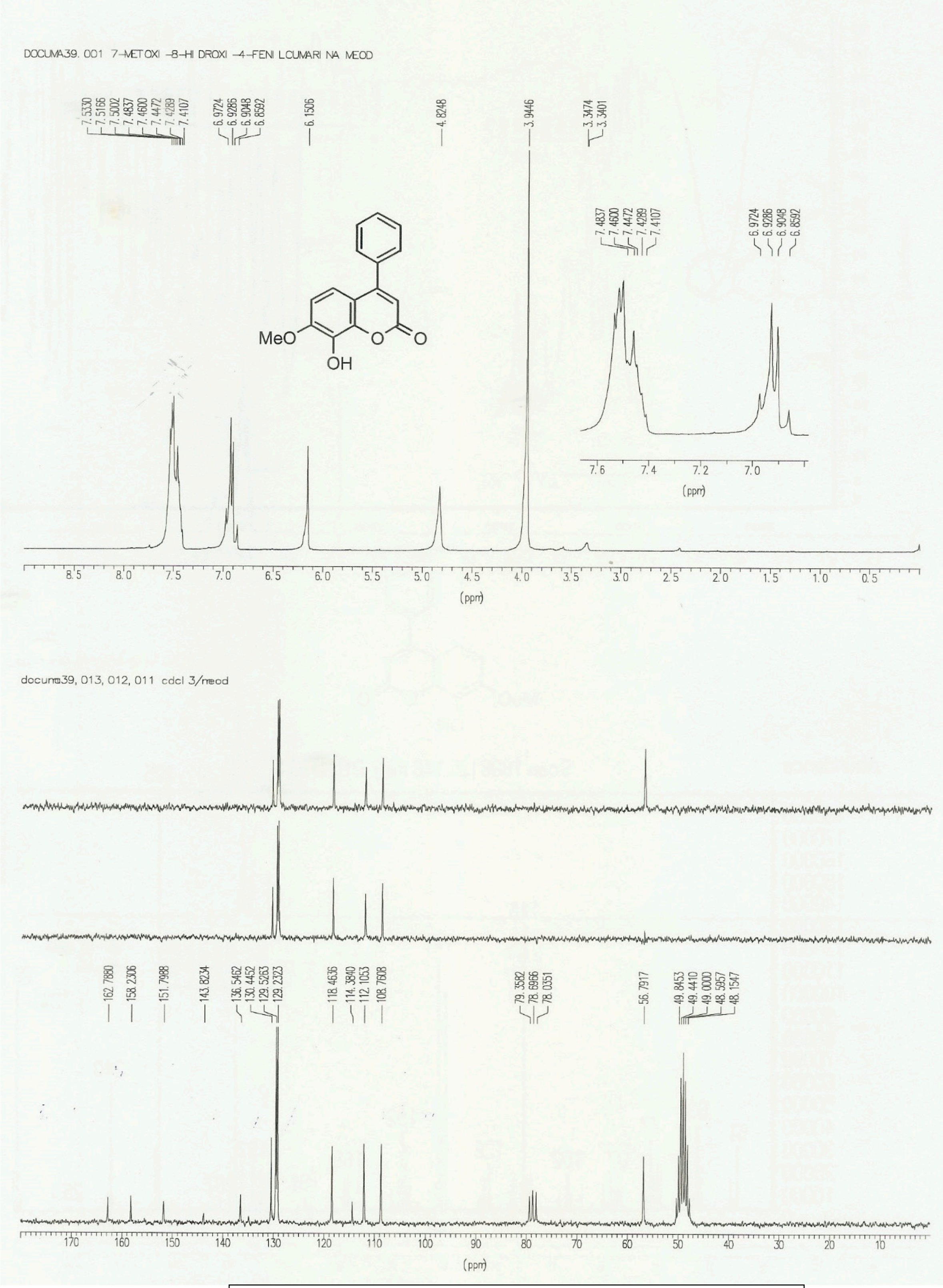

8-hydroxy-7-methoxy-4-phenyl-2H-chromen-2-one (**5**)

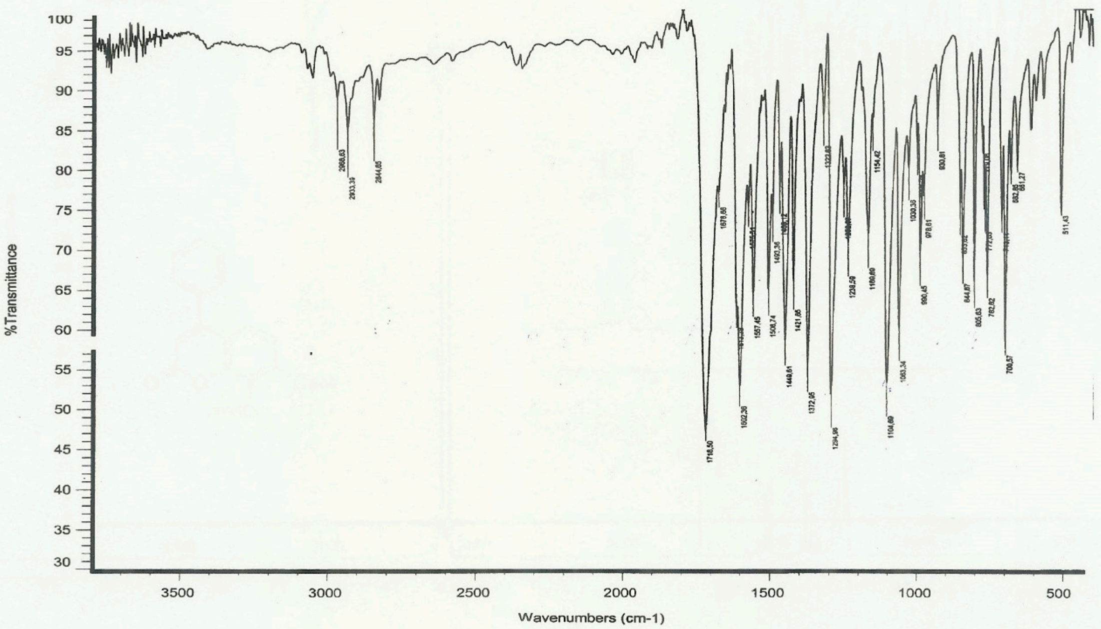

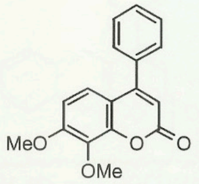

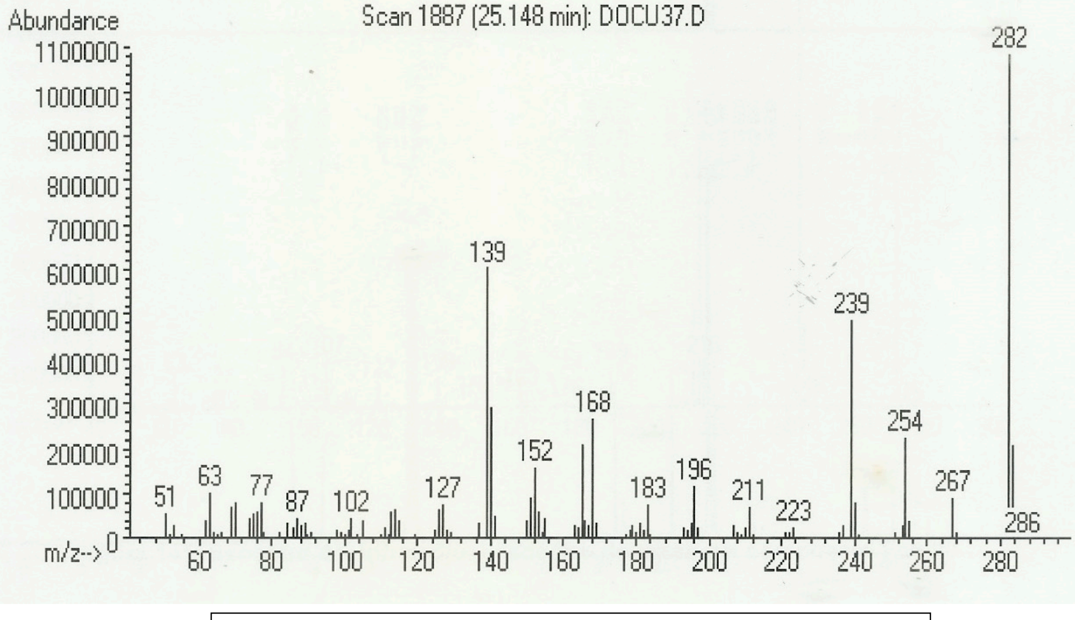

7,8-dimethoxy-4-phenyl-2H-chromen-2-one (**6**)

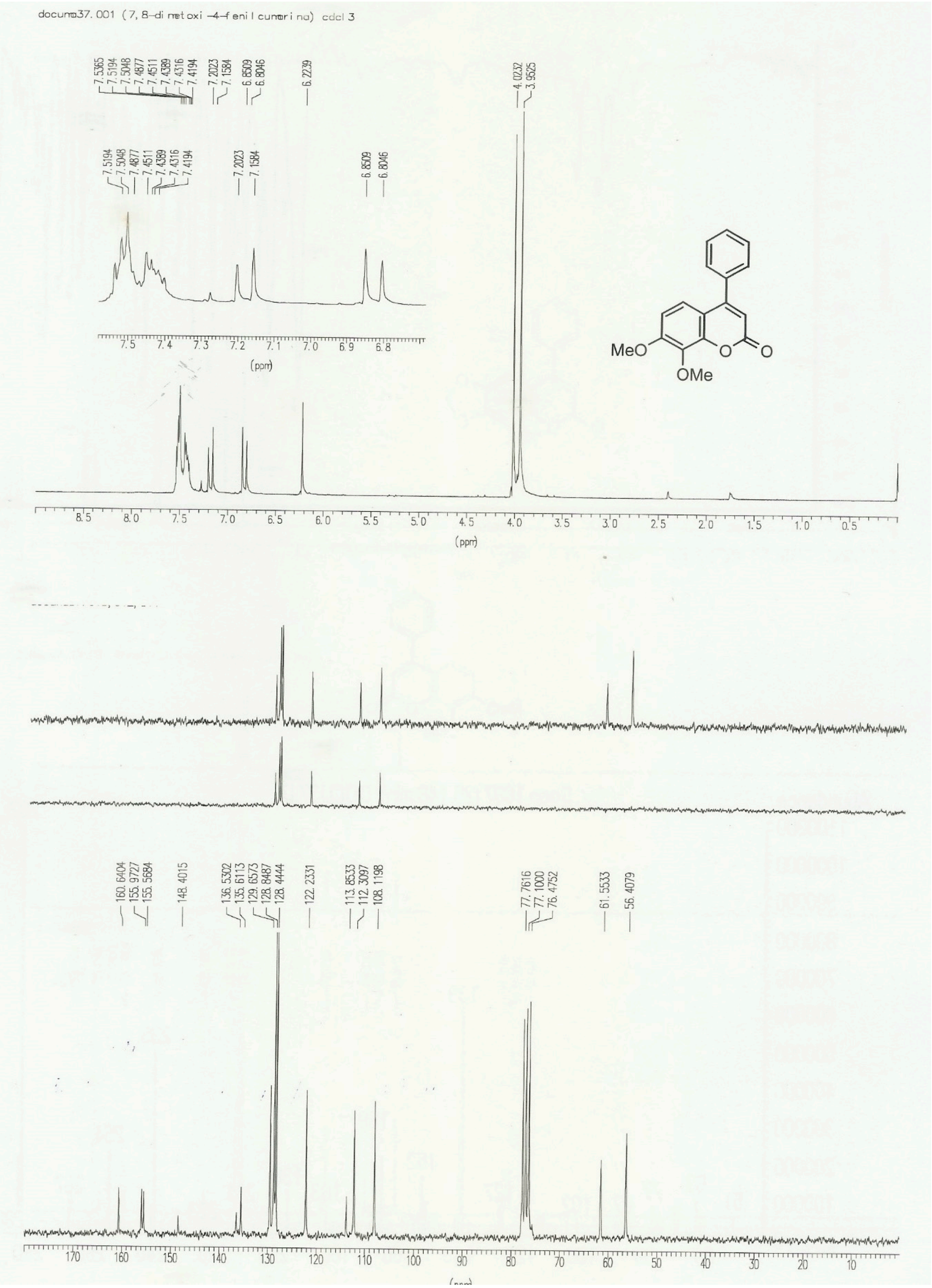

7,8-dimethoxy-4-phenyl-2H-chromen-2-one (**6**)

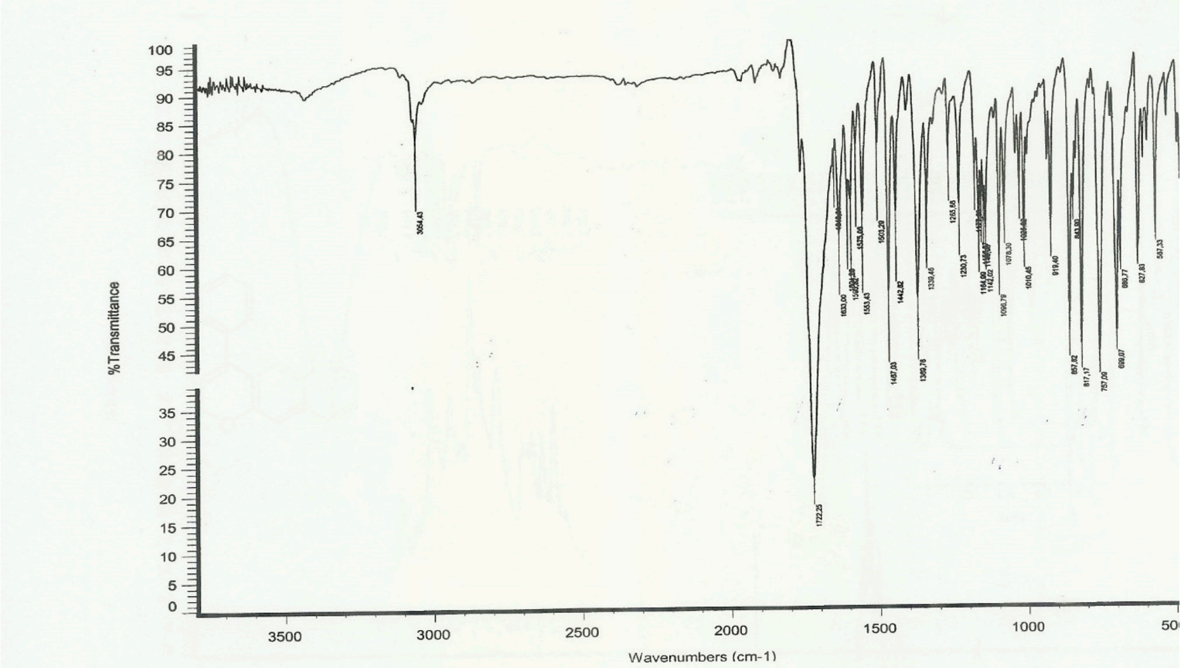

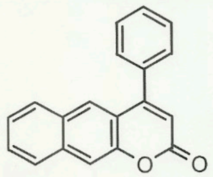

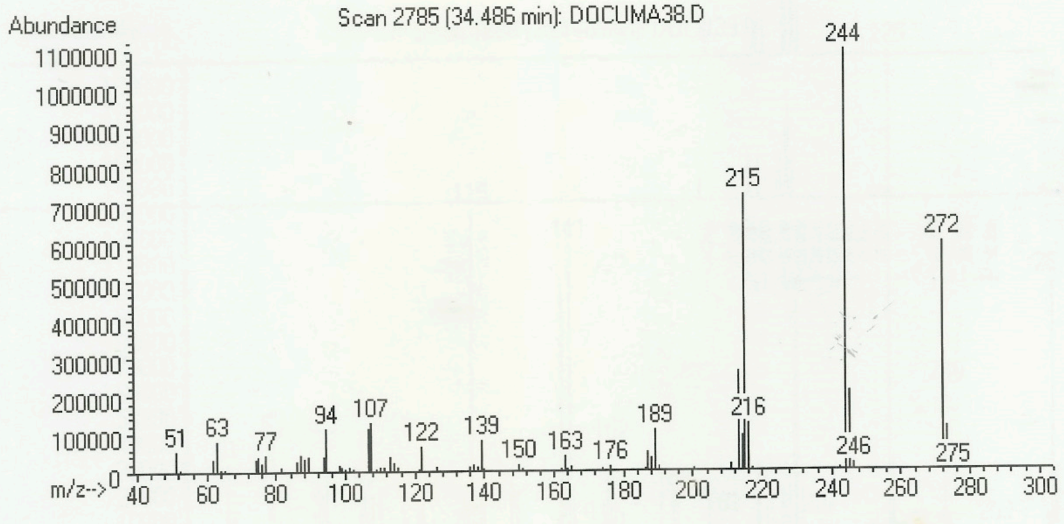

4-phenyl-2H-benzo[g]chromen-2-one (**7**)

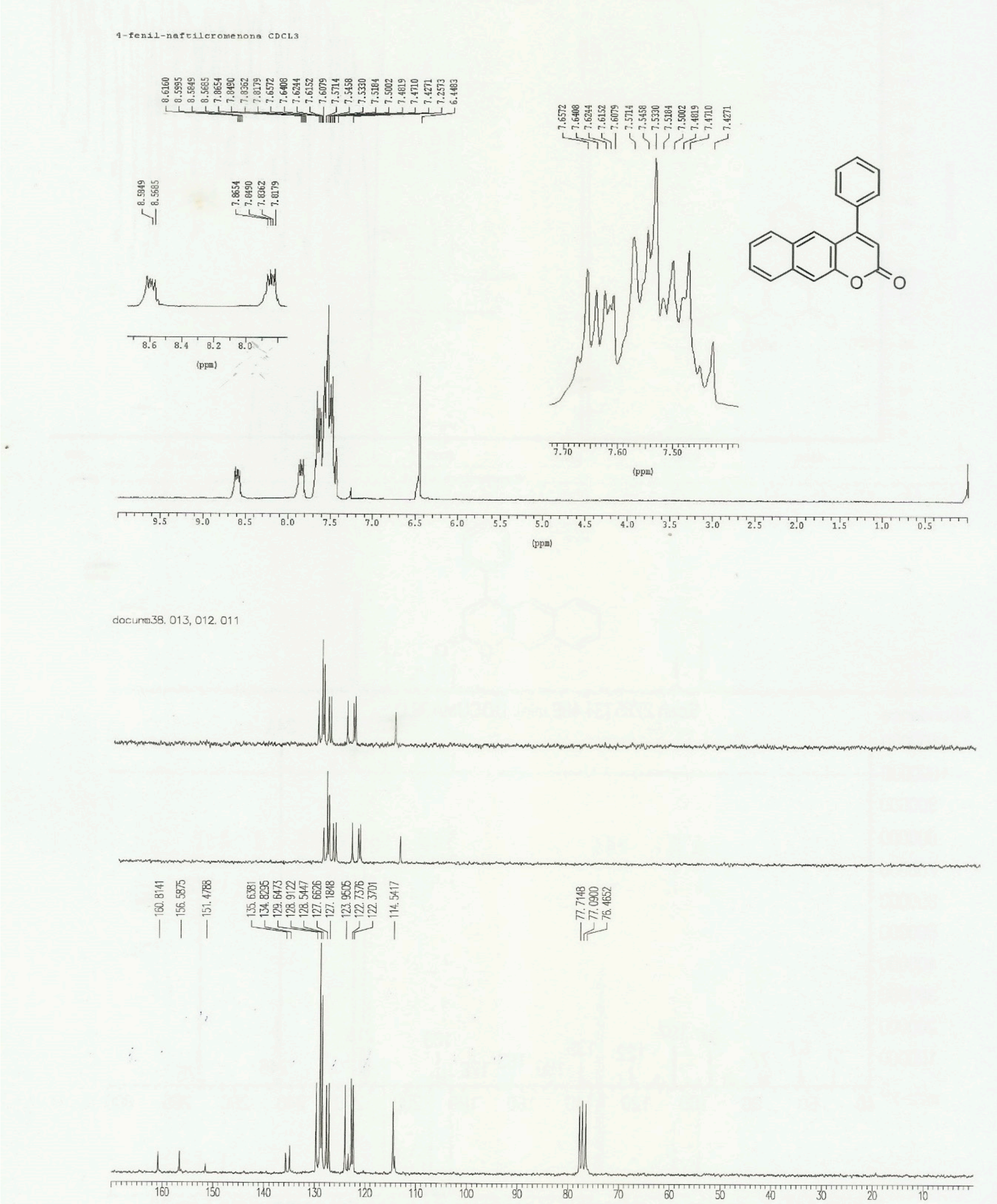

4-phenyl-2H-benzo[g]chromen-2-one (**7**)

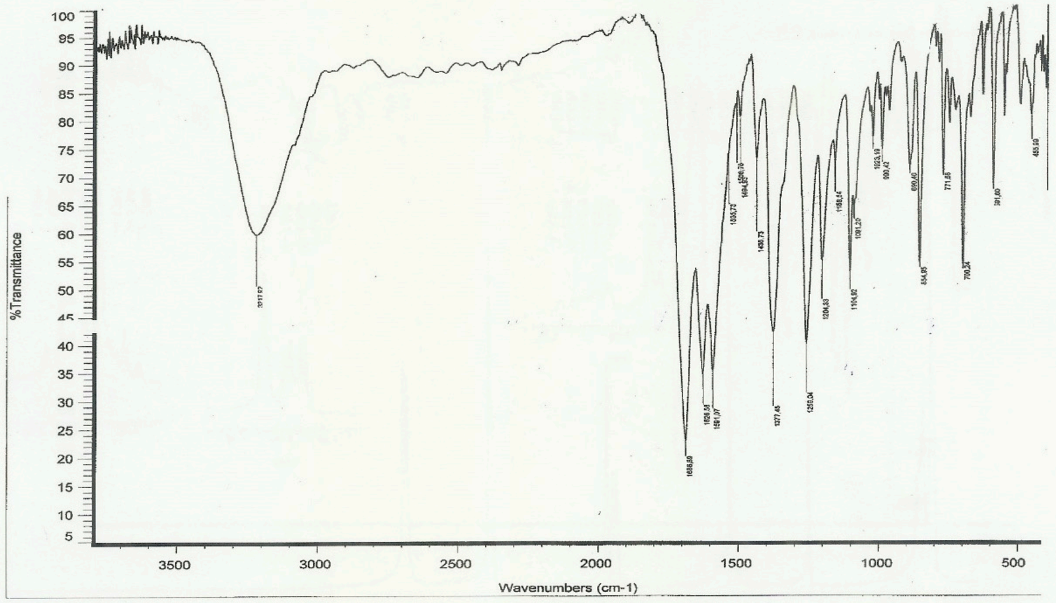

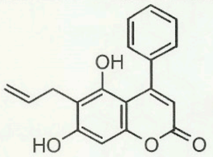

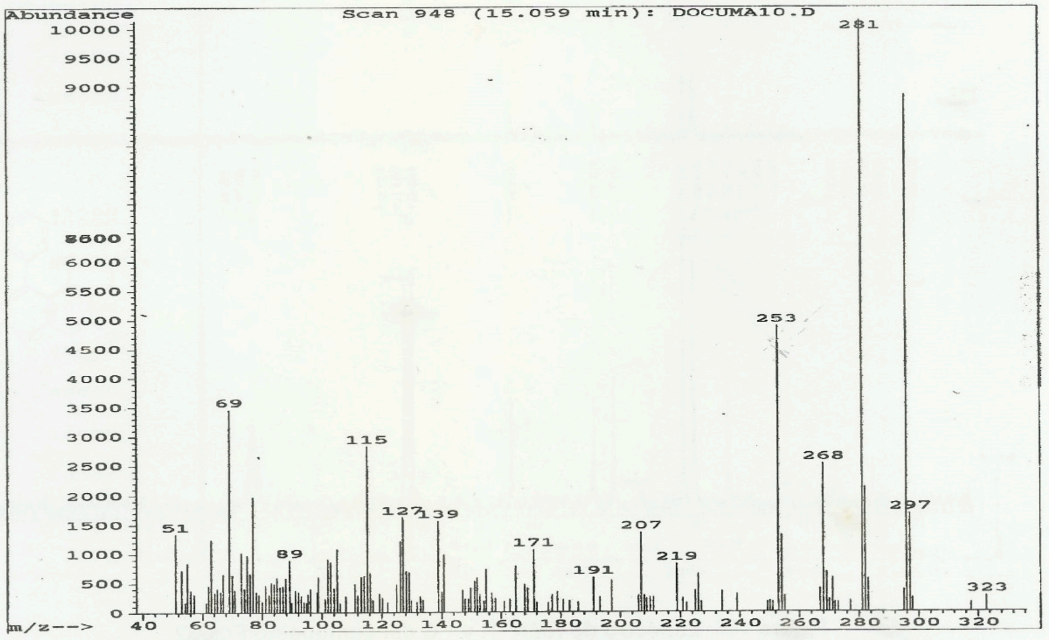

6-allyl-5,7-dihydroxy-4-phenyl-2H-chromen-2-one (**8**)

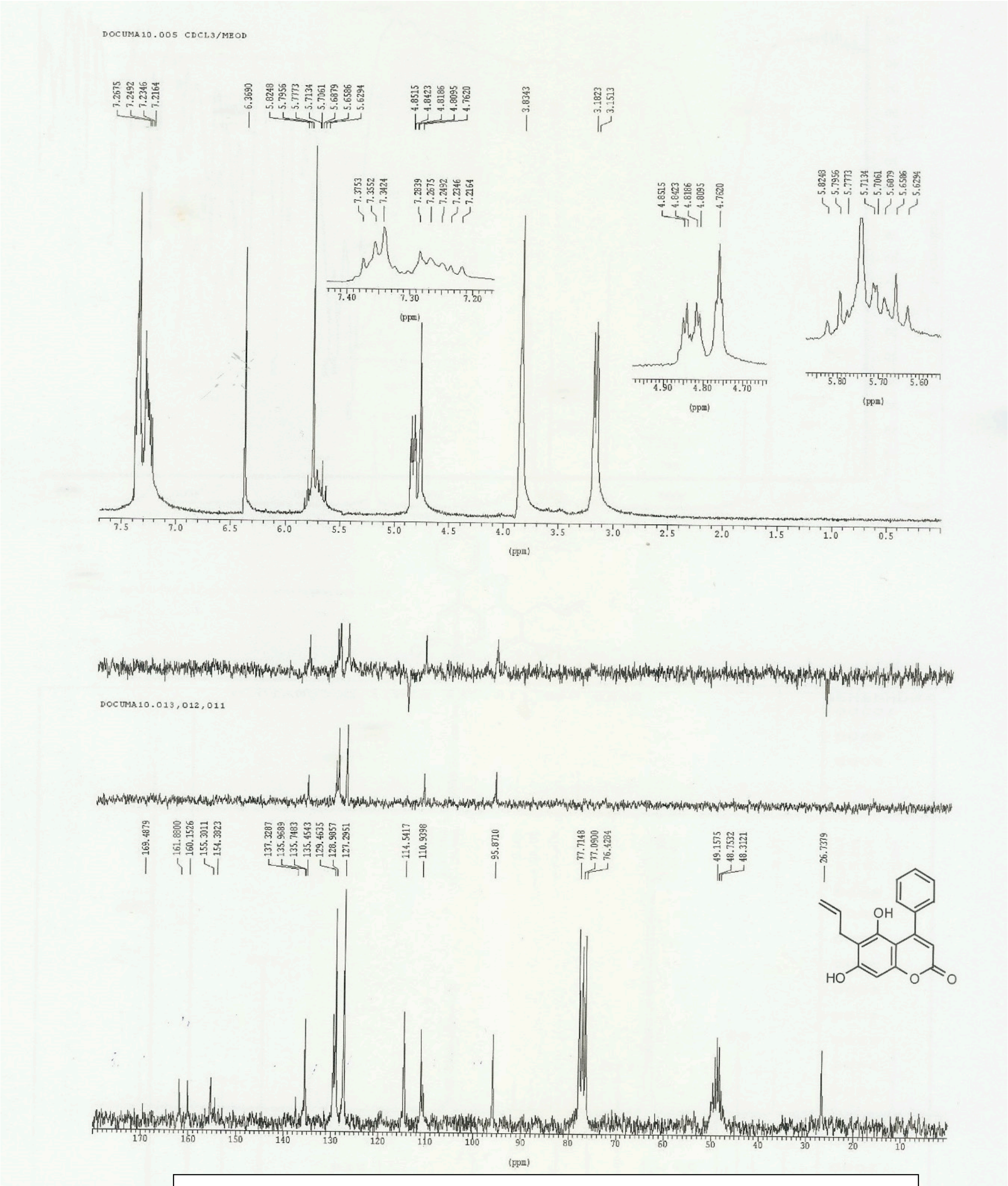

6-allyl-5,7-dihydroxy-4-phenyl-2H-chromen-2-one (**8**)

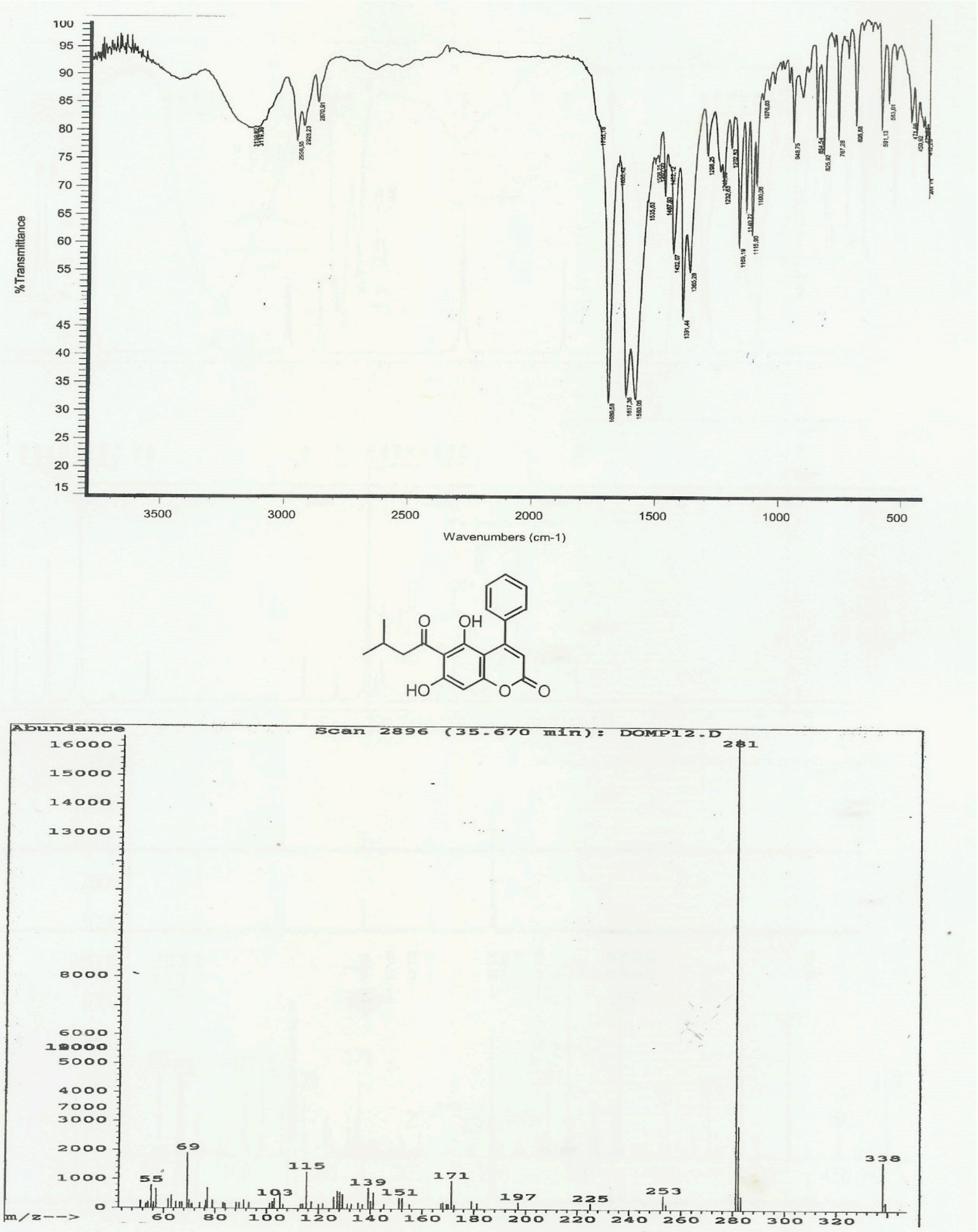

5,7-dihydroxy-6-(3-methylbutanoyl)-4-phenyl-2H-chromen-2-one (**9**)

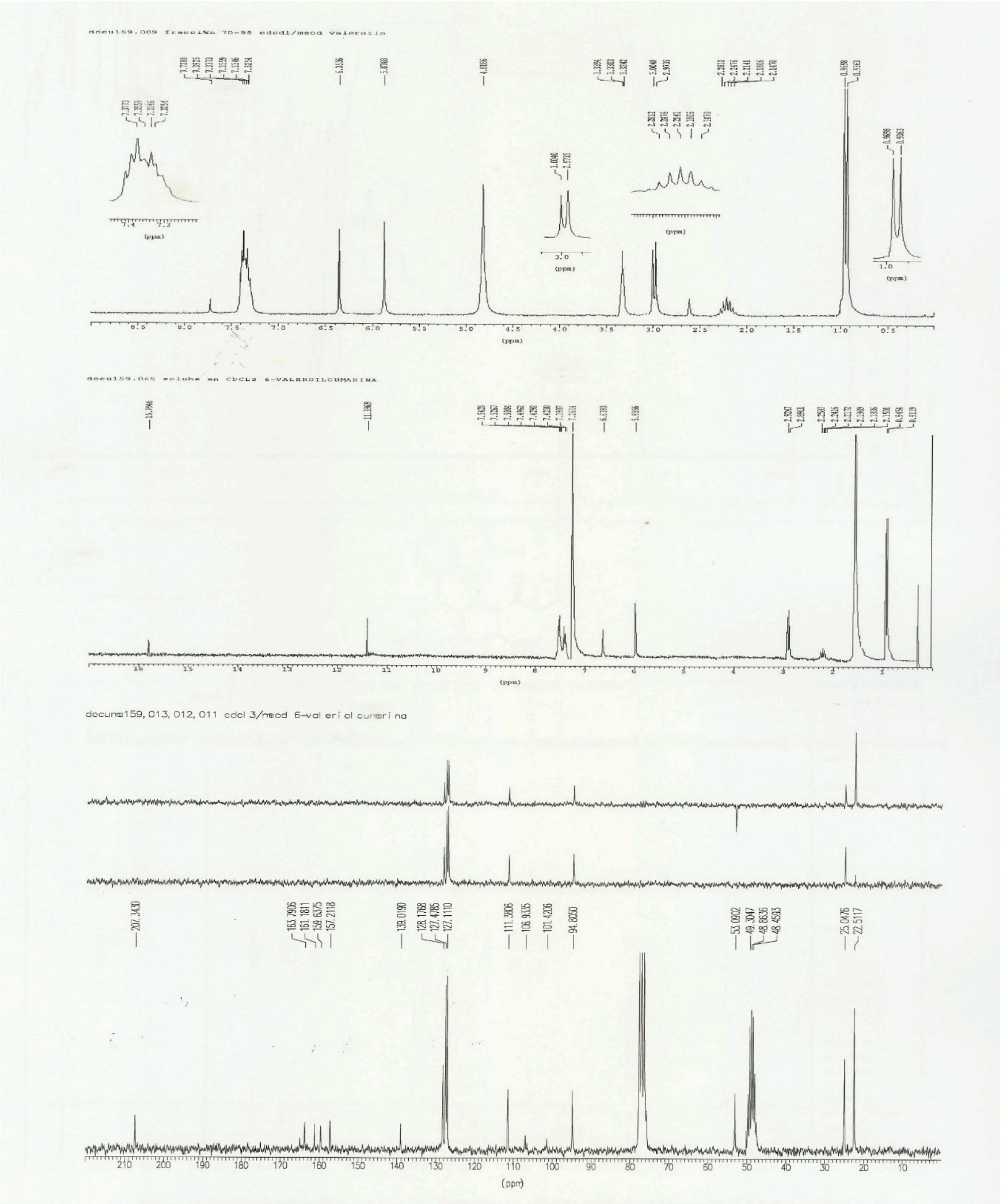

5,7-dihydroxy-6-(3-methylbutanoyl)-4-phenyl-2H-chromen-2-one (**9**)

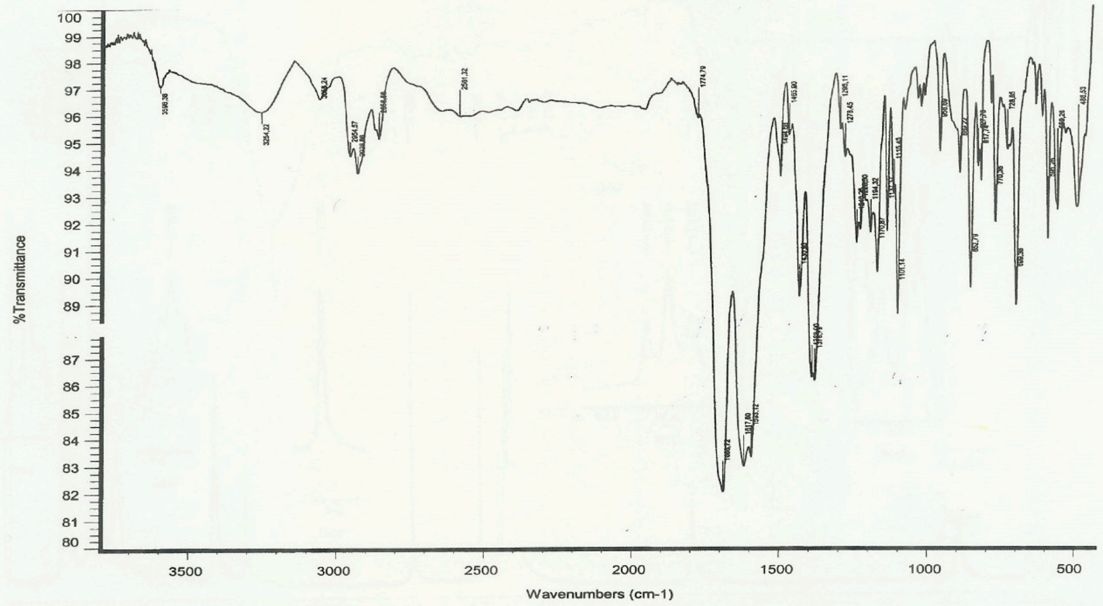

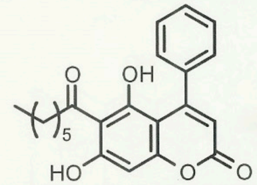

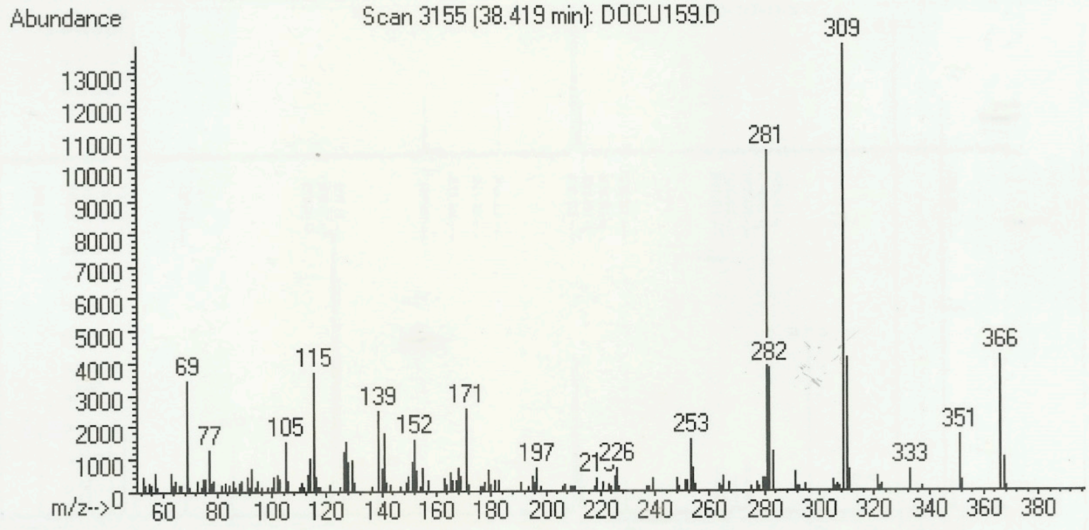

6-heptanoyl-5,7-dihydroxy-4-phenyl-2H-chromen-2-one (**10**)

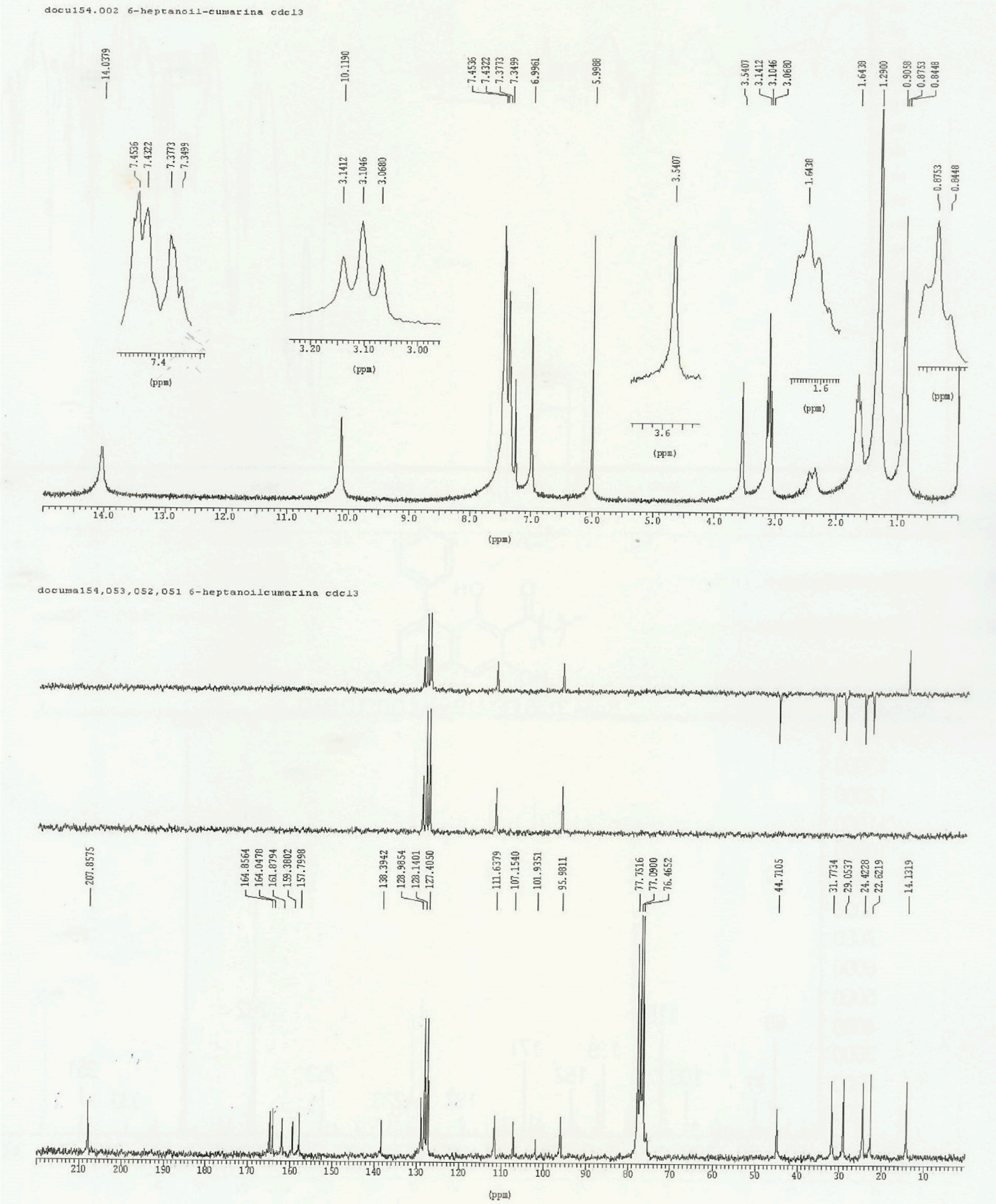

6-heptanoyl-5,7-dihydroxy-4-phenyl-2H-chromen-2-one (**10**)

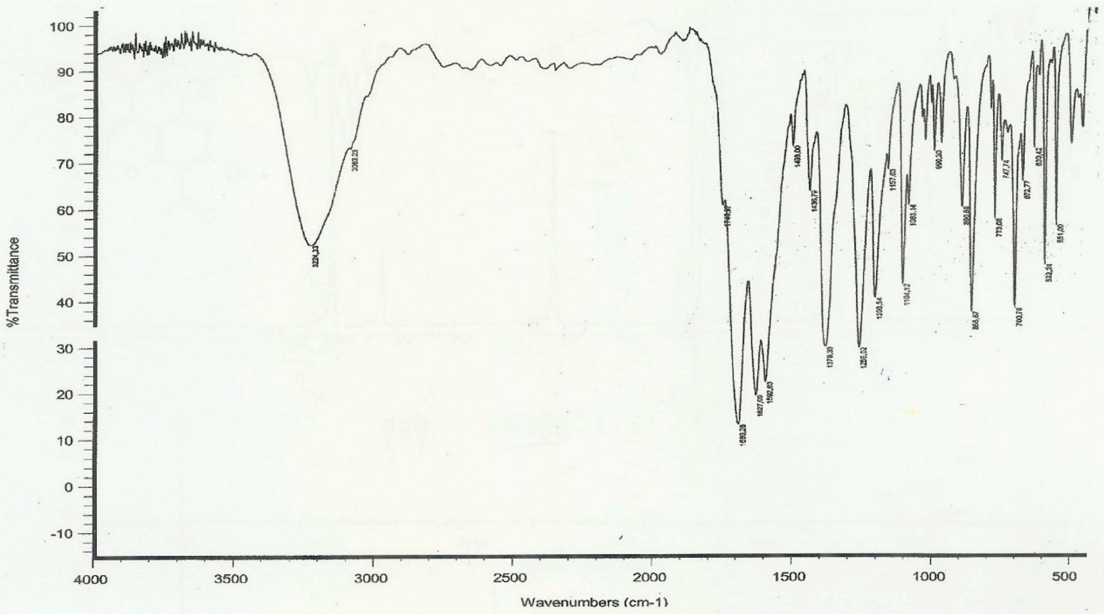

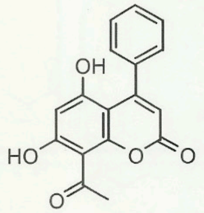

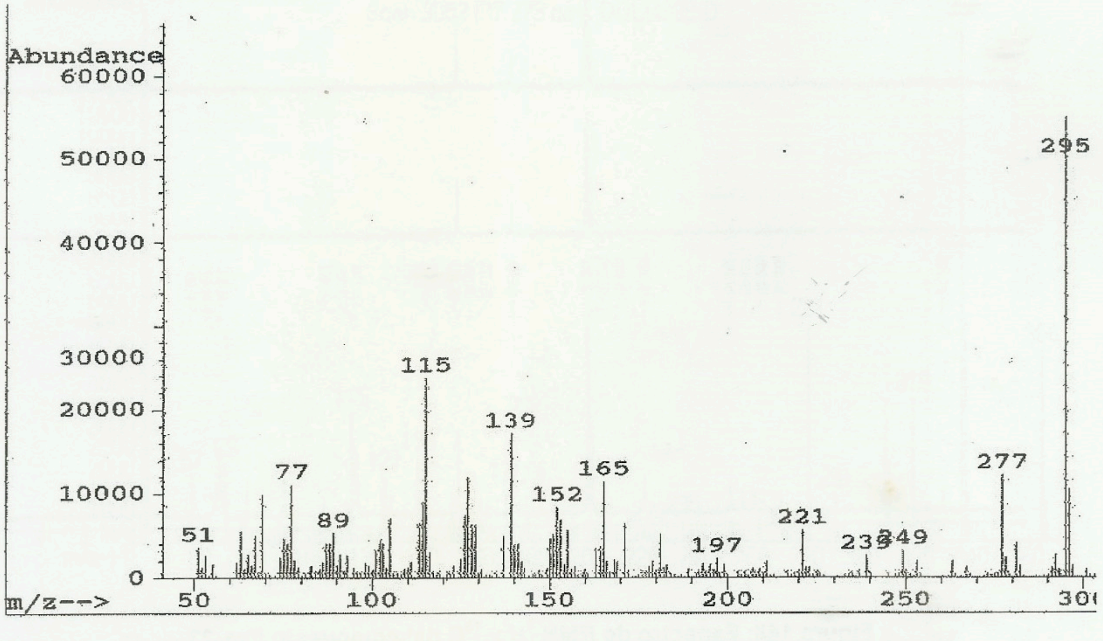

8-acetyl-5,7-dihydroxy-4-phenyl-2H-chromen-2-one (**12**)

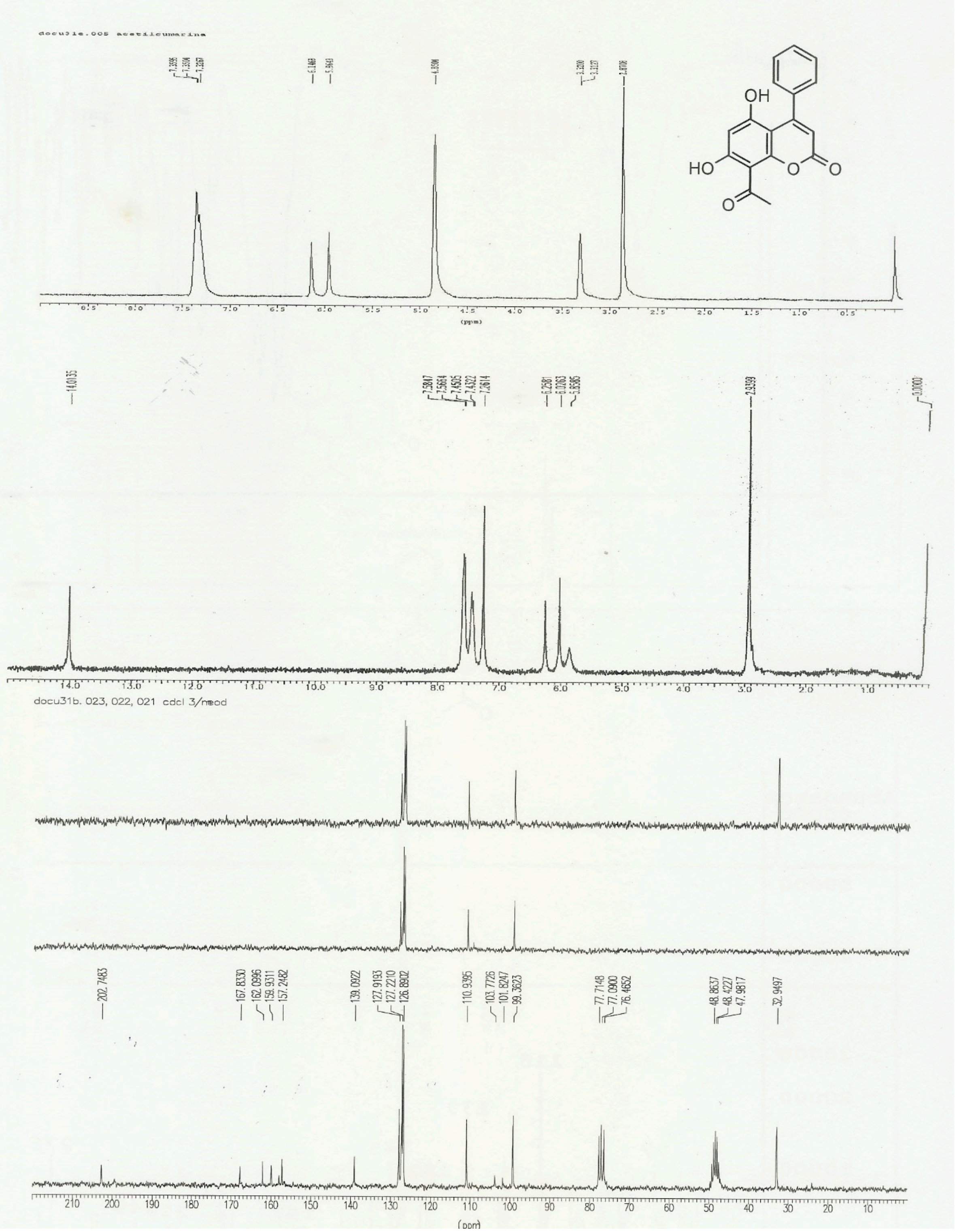

8-acetyl-5,7-dihydroxy-4-phenyl-2H-chromen-2-one (**12**)

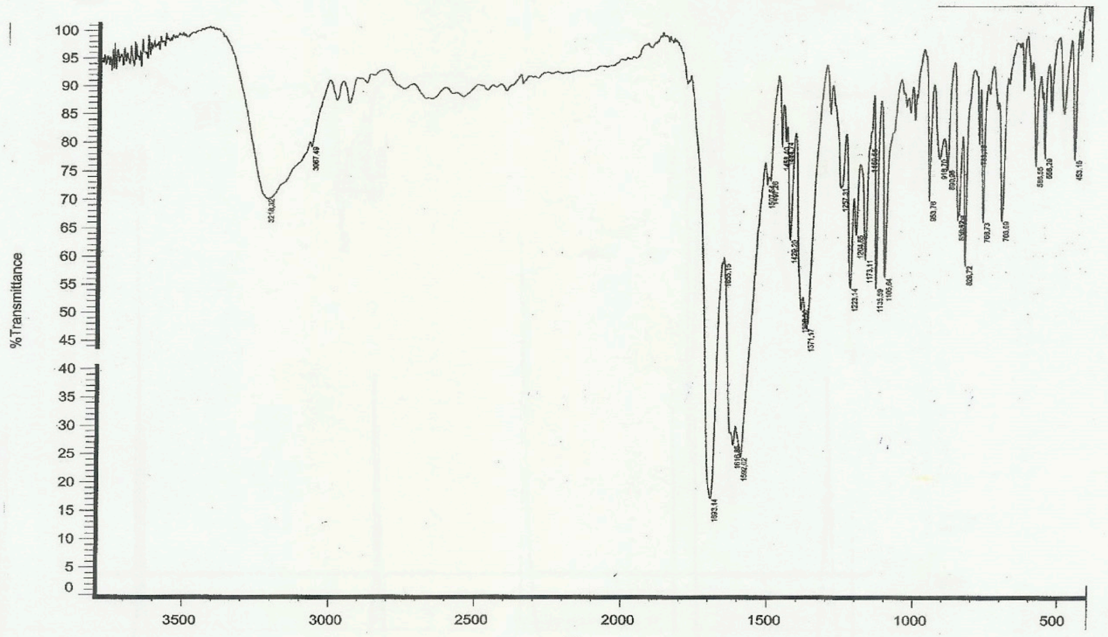

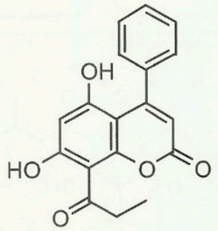

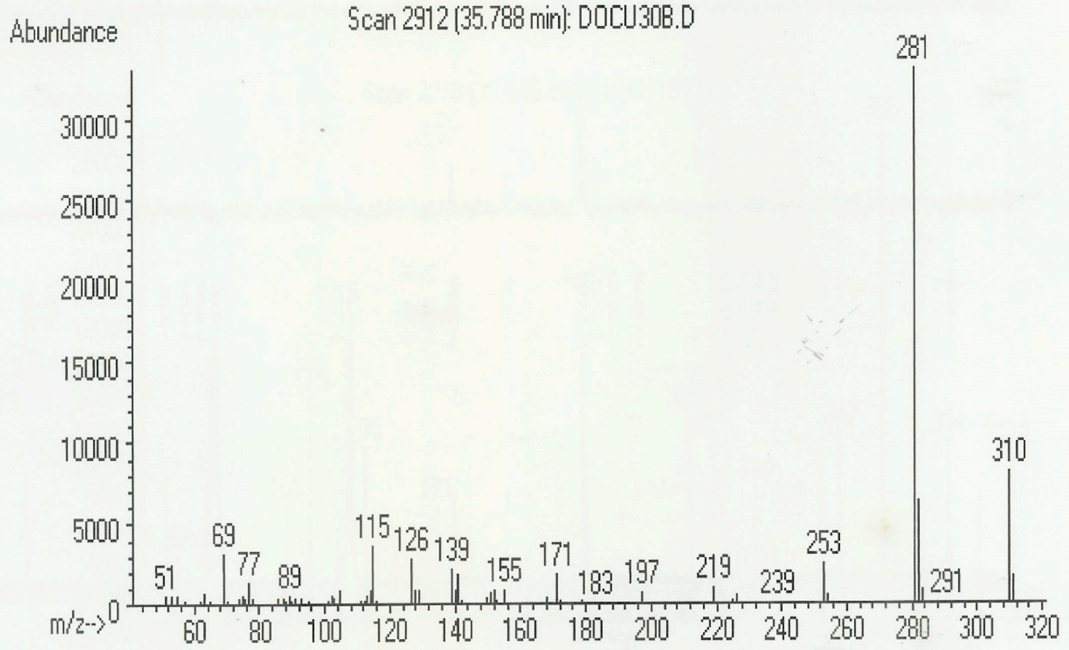

5,7-dihydroxy-4-phenyl-8-propionyl-2H-chromen-2-one (**13**)

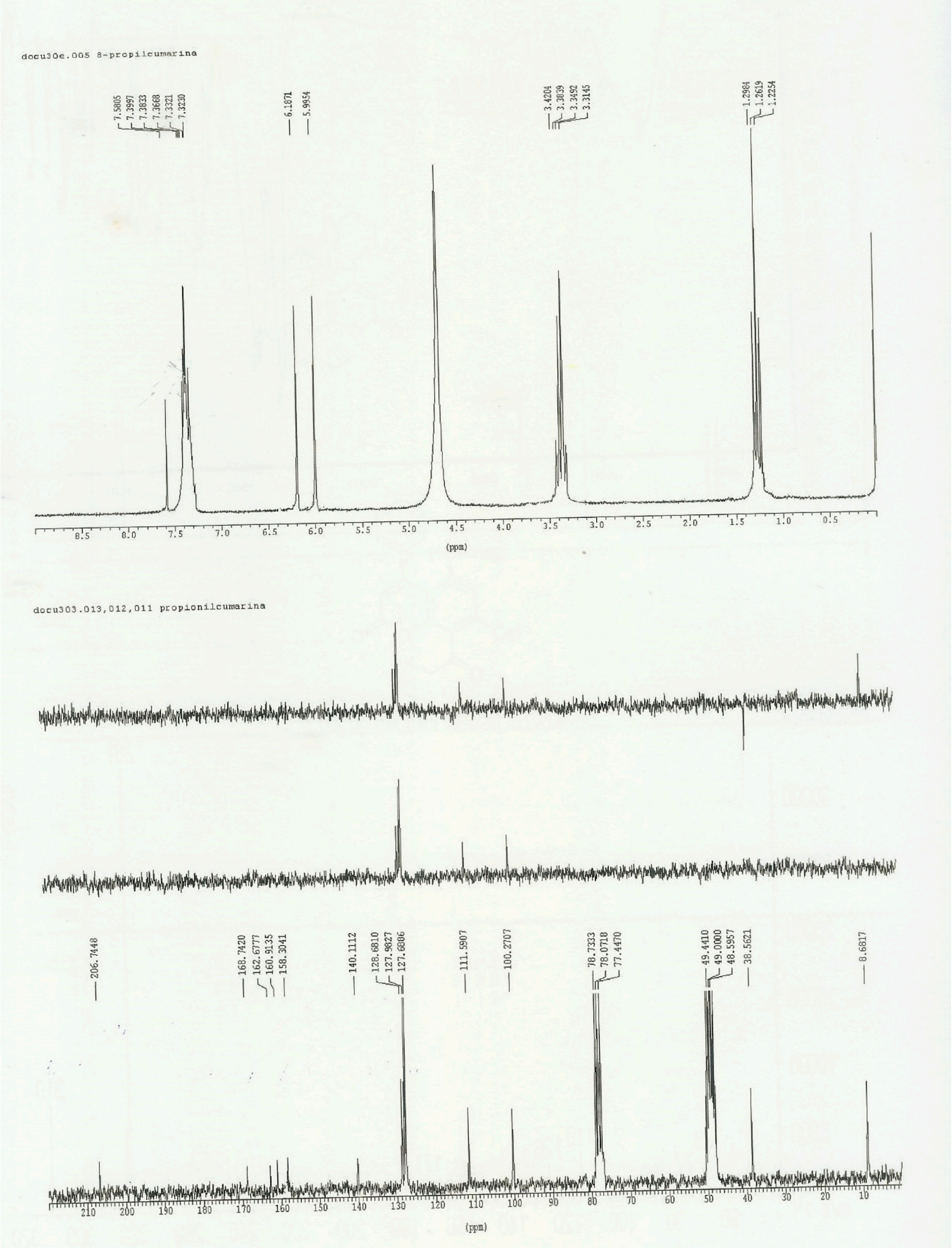

5,7-dihydroxy-4-phenyl-8-propionyl-2H-chromen-2-one (**13**)

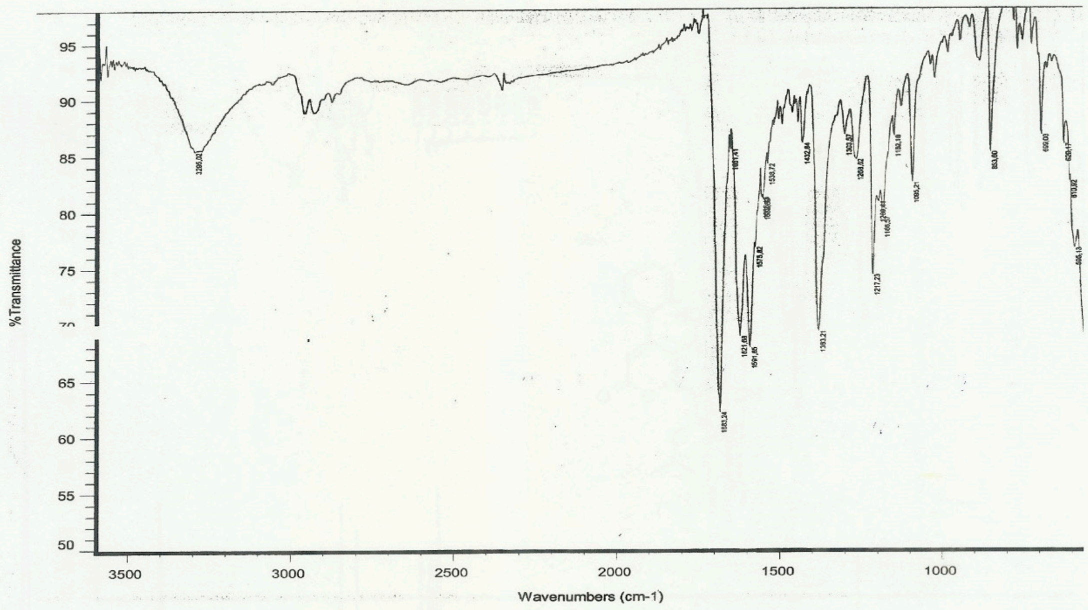

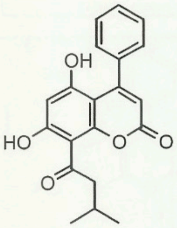

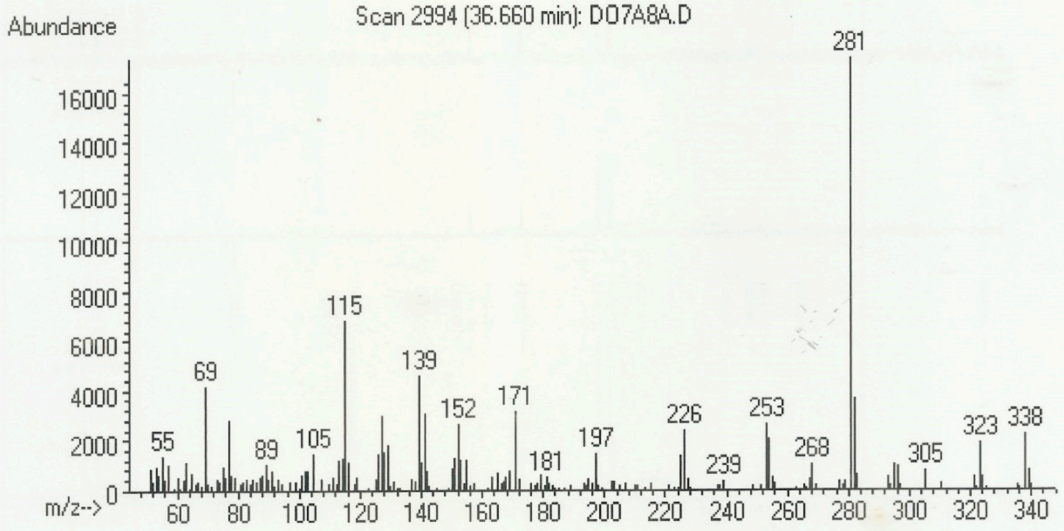

5,7-dihydroxy-8-(3-methylbutanoyl)-4-phenyl-2H-chromen-2-one (**14**)

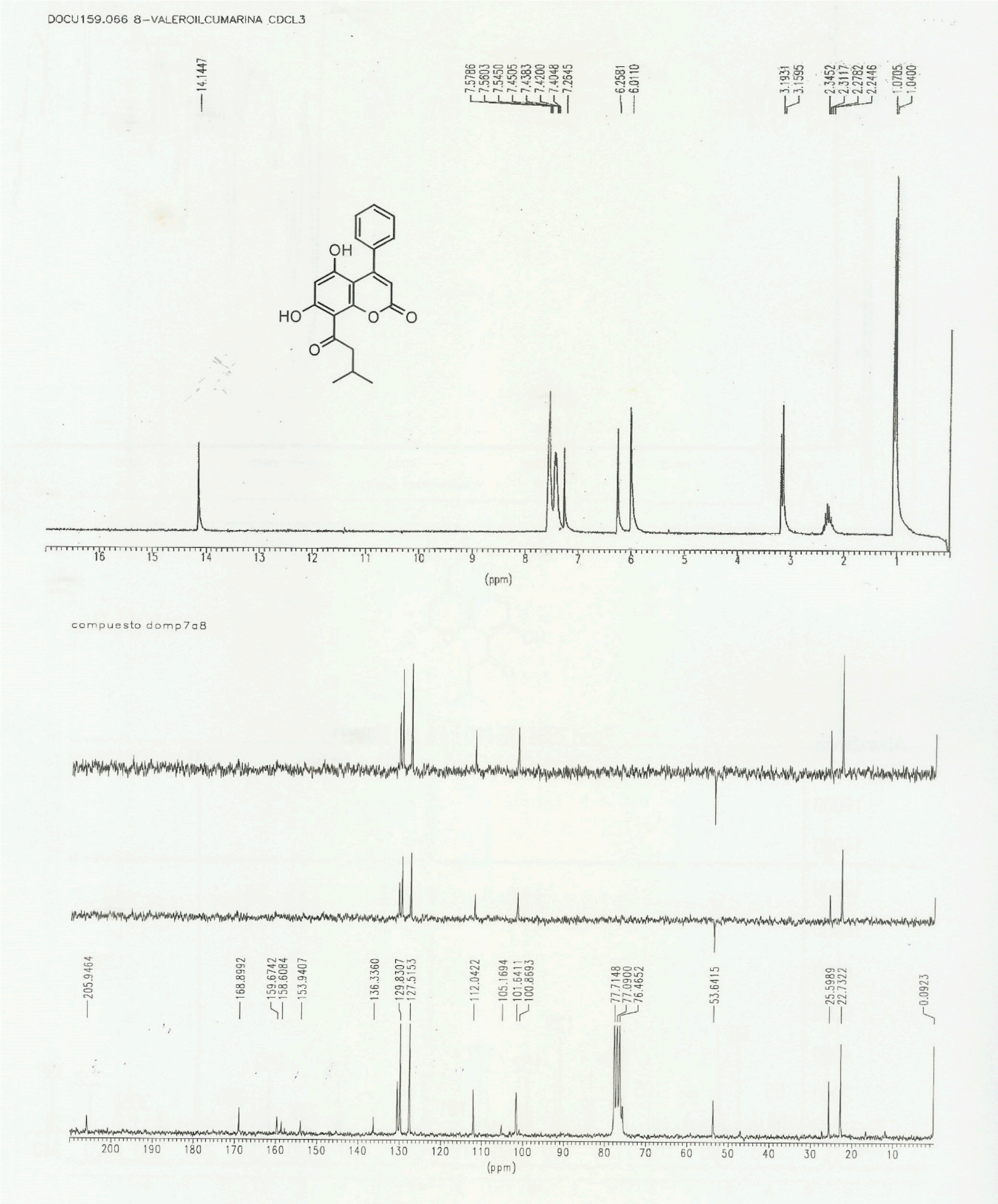

5,7-dihydroxy-8-(3-methylbutanoyl)-4-phenyl-2H-chromen-2-one (**14**)

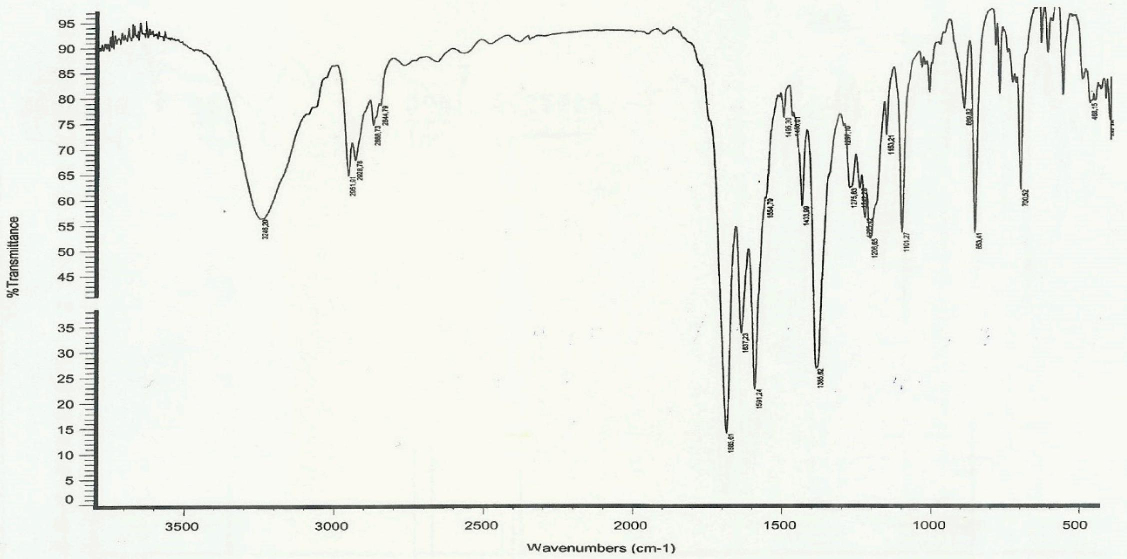

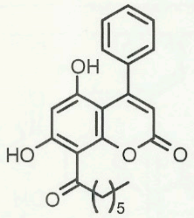

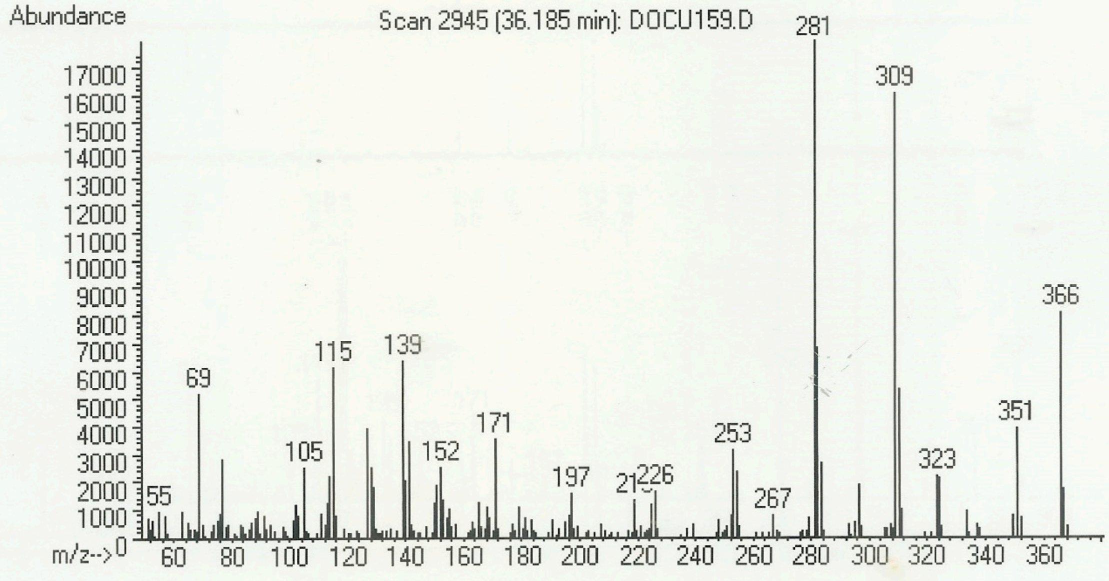

8-heptanoyl-5,7-dihydroxy-4-phenyl-2H-chromen-2-one (**15**)

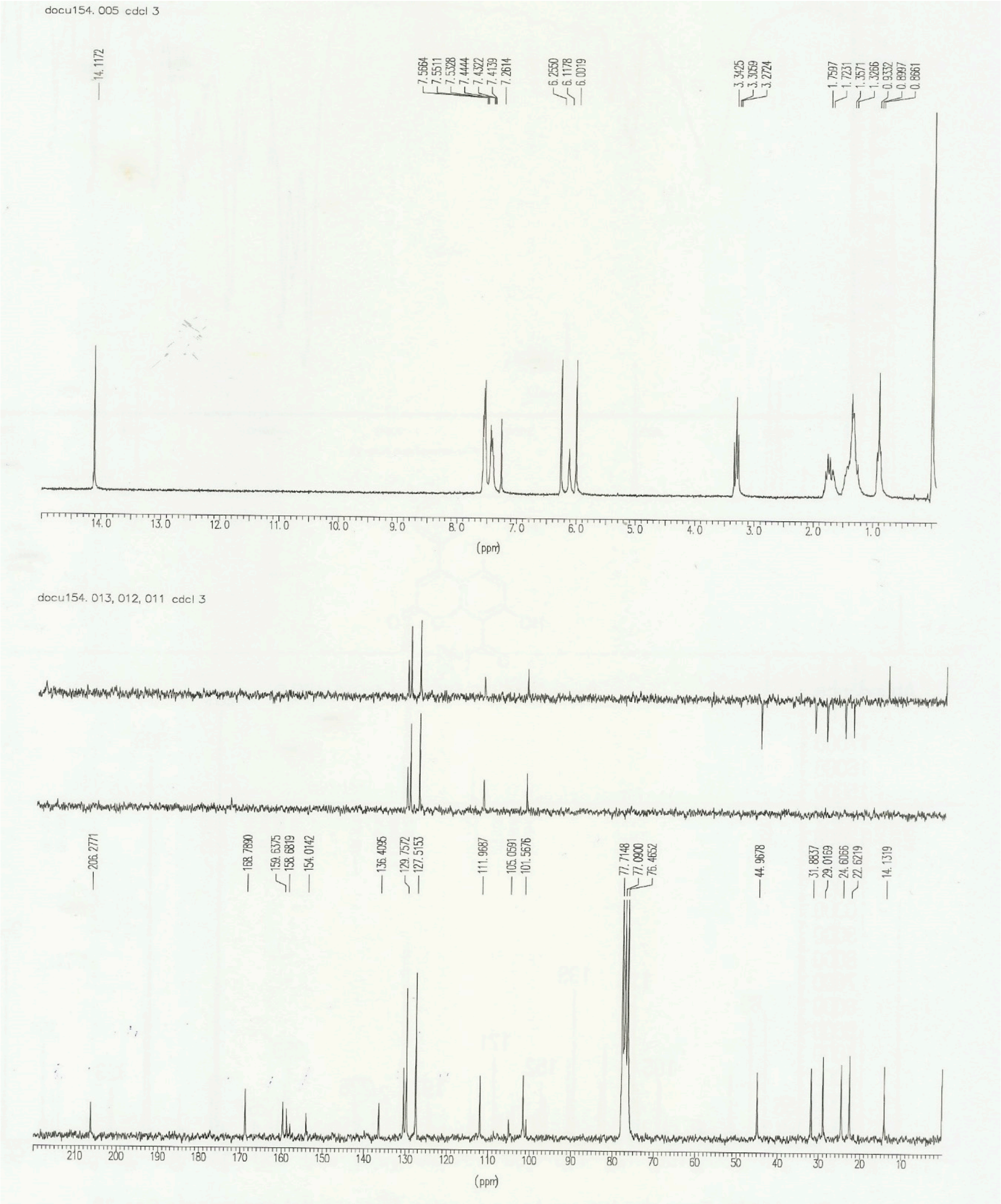

8-heptanoyl-5,7-dihydroxy-4-phenyl-2H-chromen-2-one (**15**)

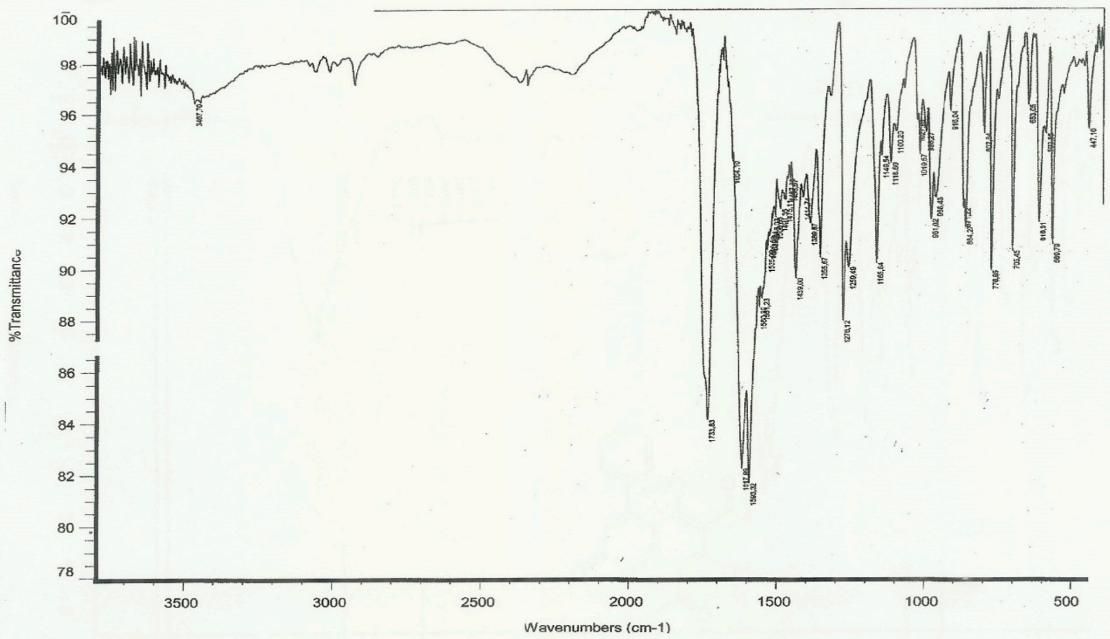

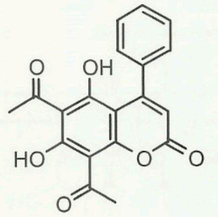

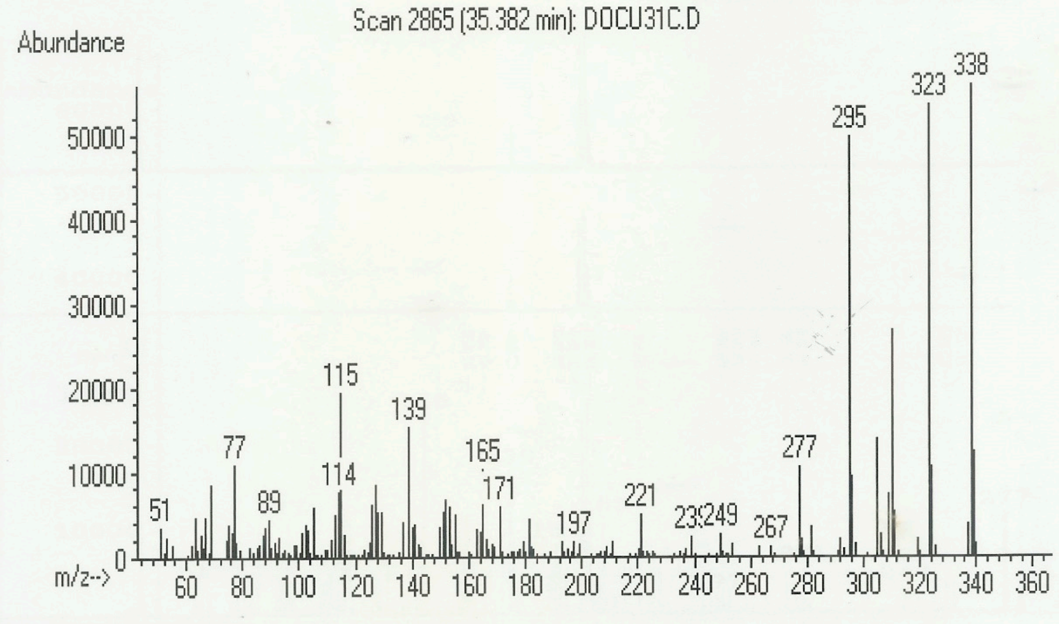

6,8-diacetyl-5,7-dihydroxy-4-phenyl-2H-chromen-2-one (**17**)

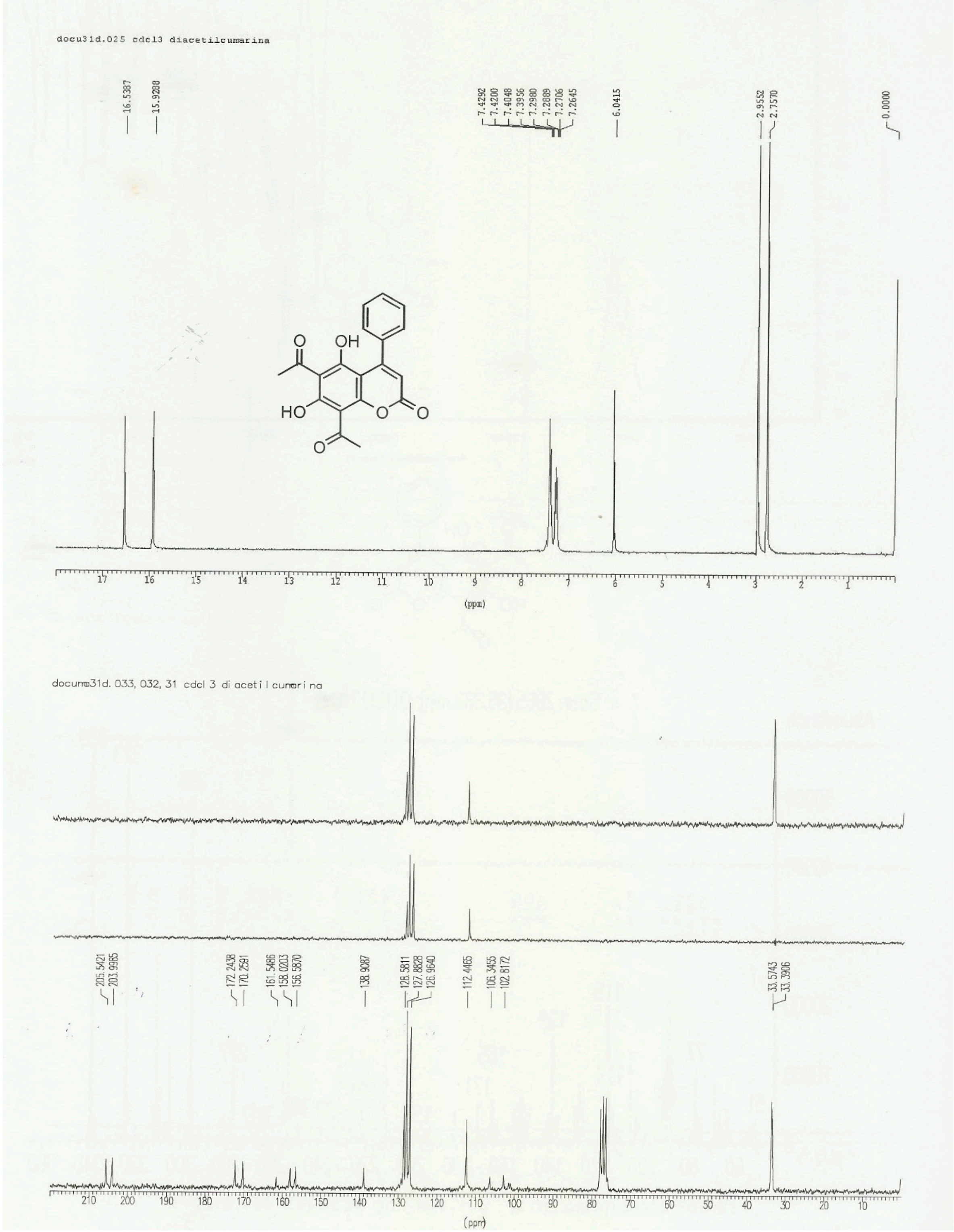

6,8-diacetyl-5,7-dihydroxy-4-phenyl-2H-chromen-2-one (**17**)

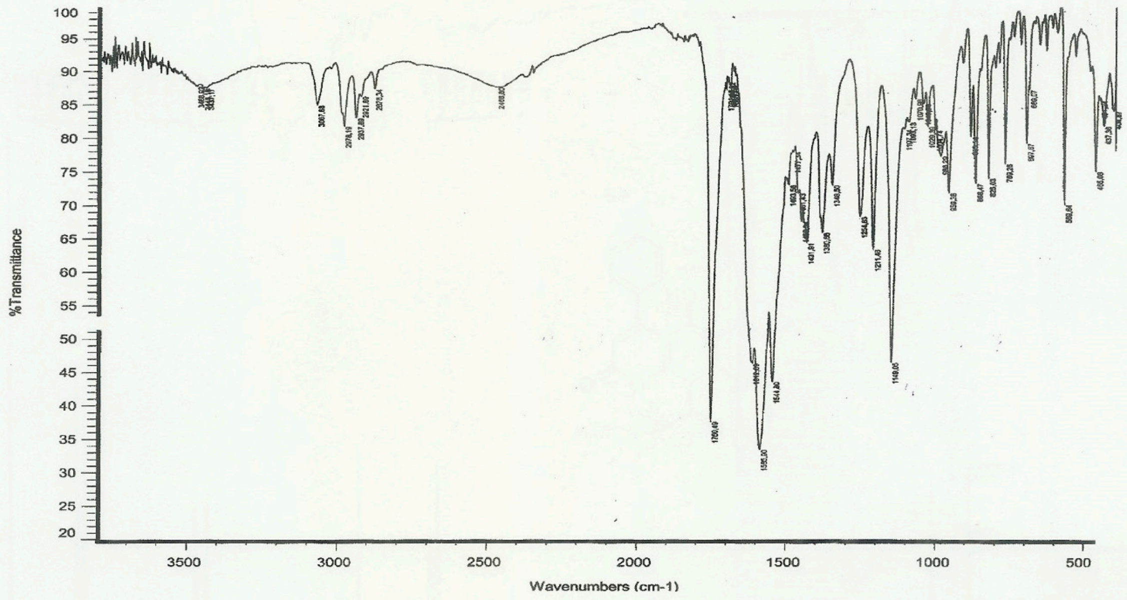

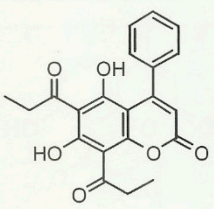

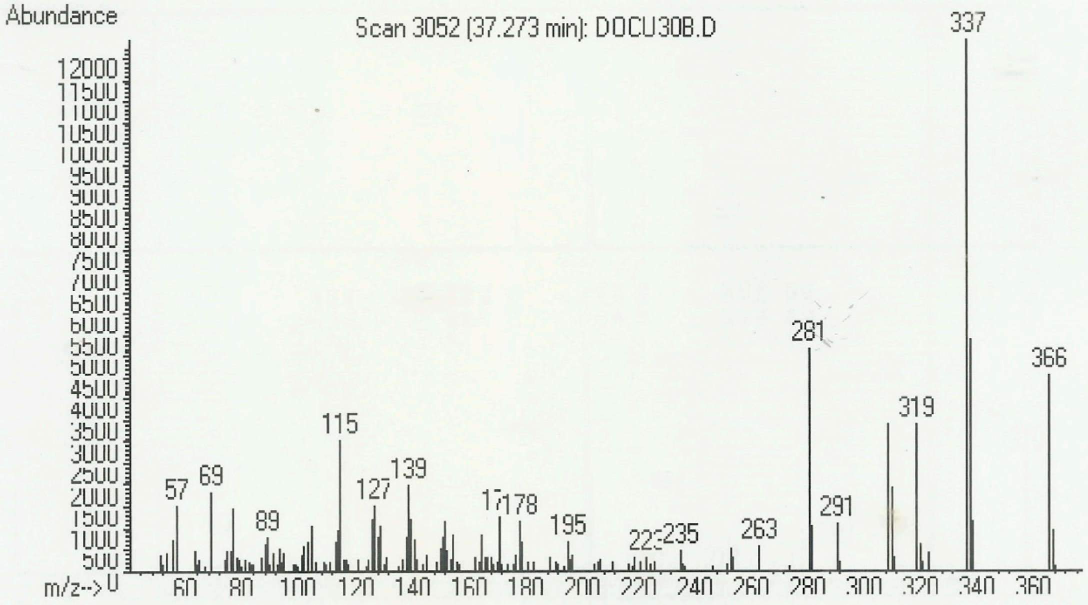

5,7-dihydroxy-4-phenyl-6,8-dipropionyl-2H-chromen-2-one (**18**)

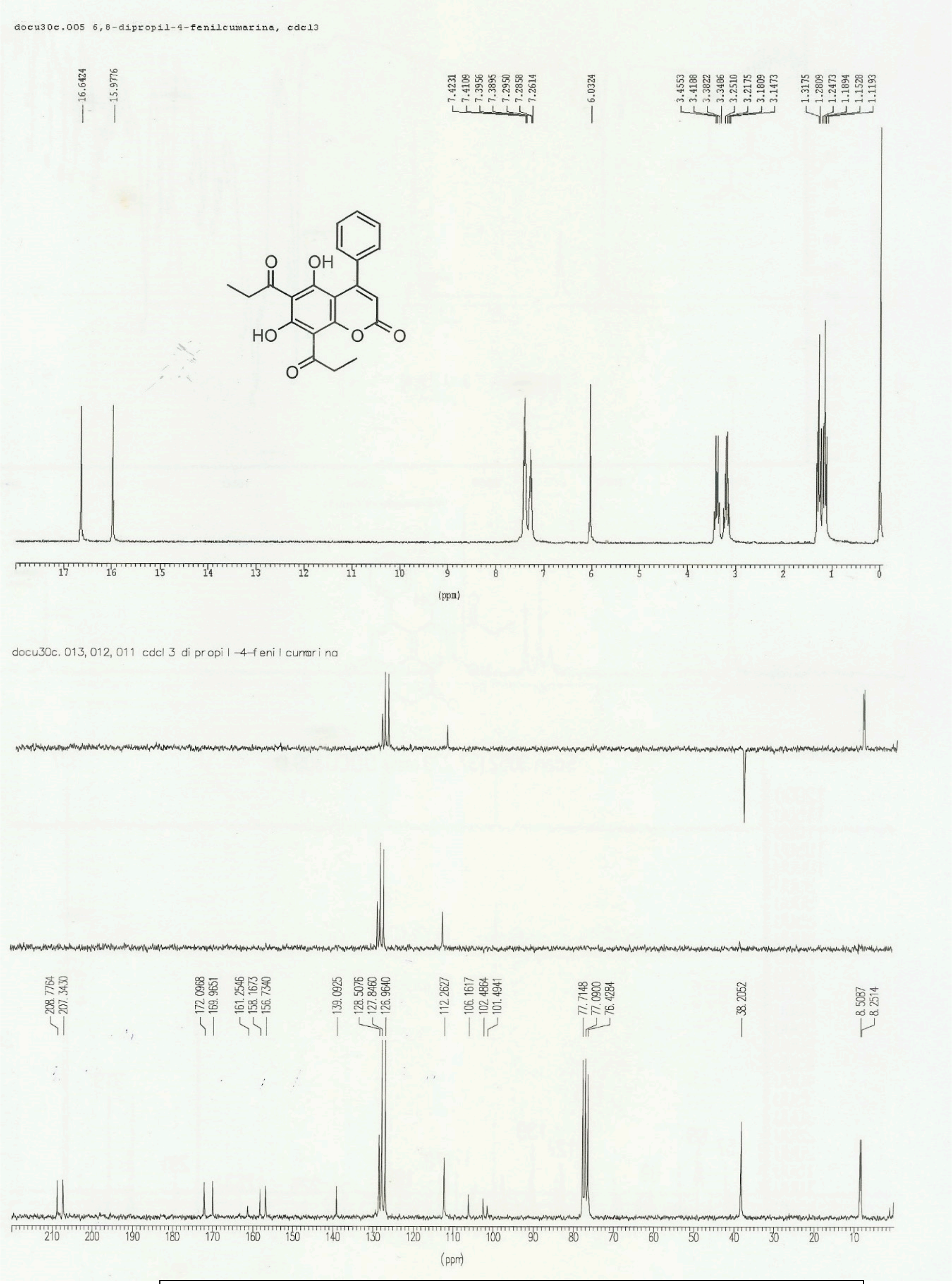

5,7-dihydroxy-4-phenyl-6,8-dipropionyl-2H-chromen-2-one (**18**)

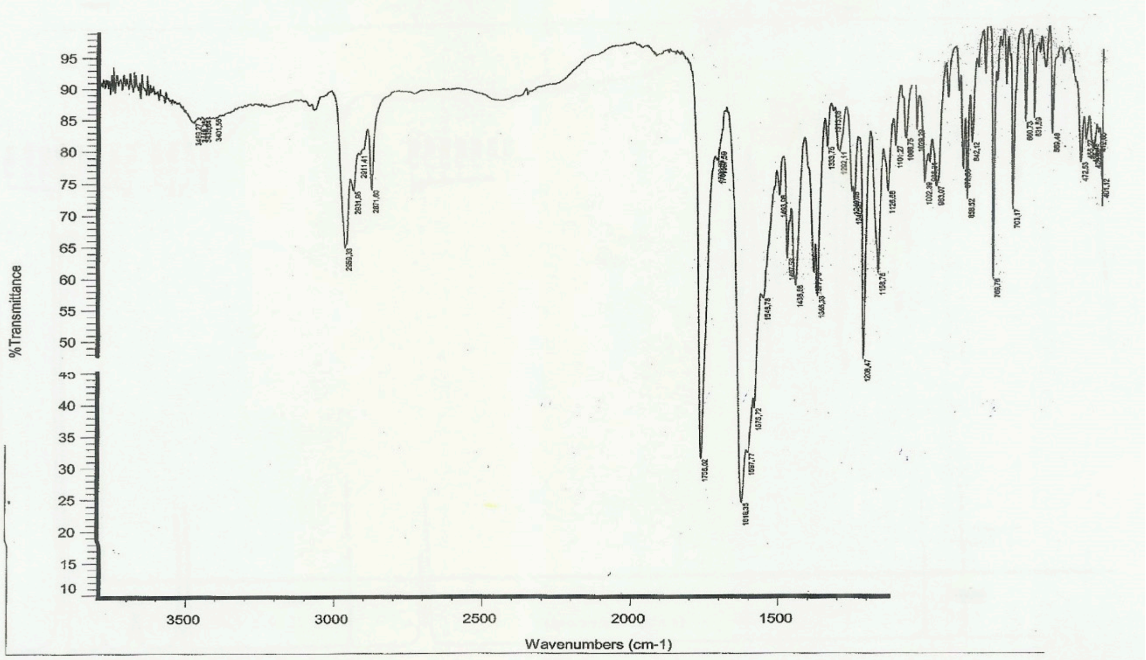

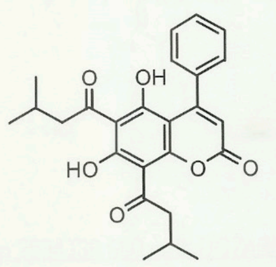

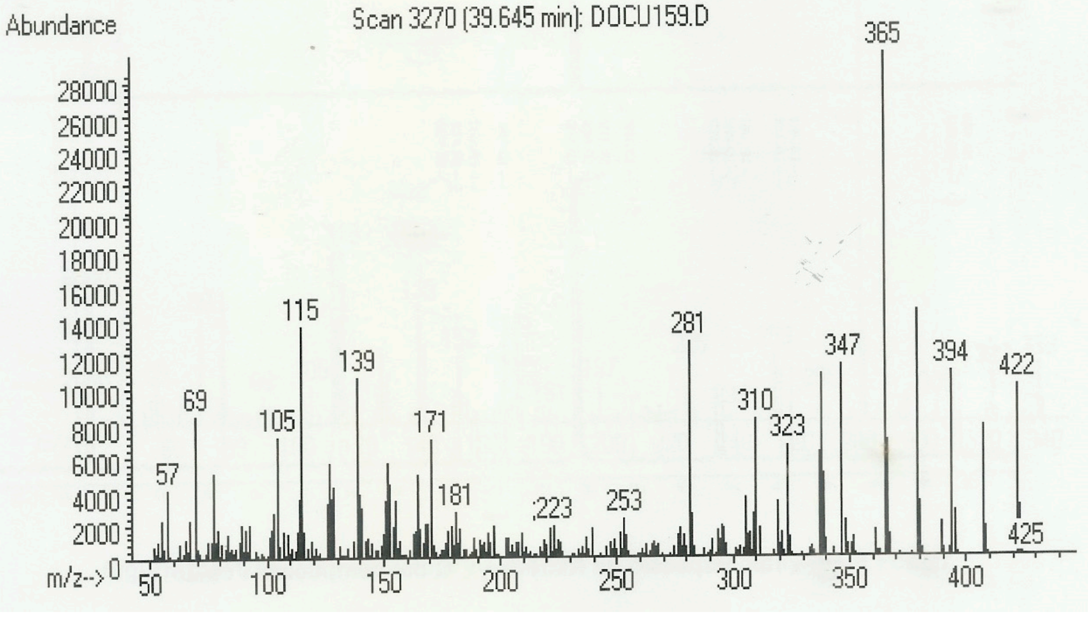

5,7-dihydroxy-6,8-bis(3-methylbutanoyl)-4-phenyl-2H-chromen-2-one (**19**)

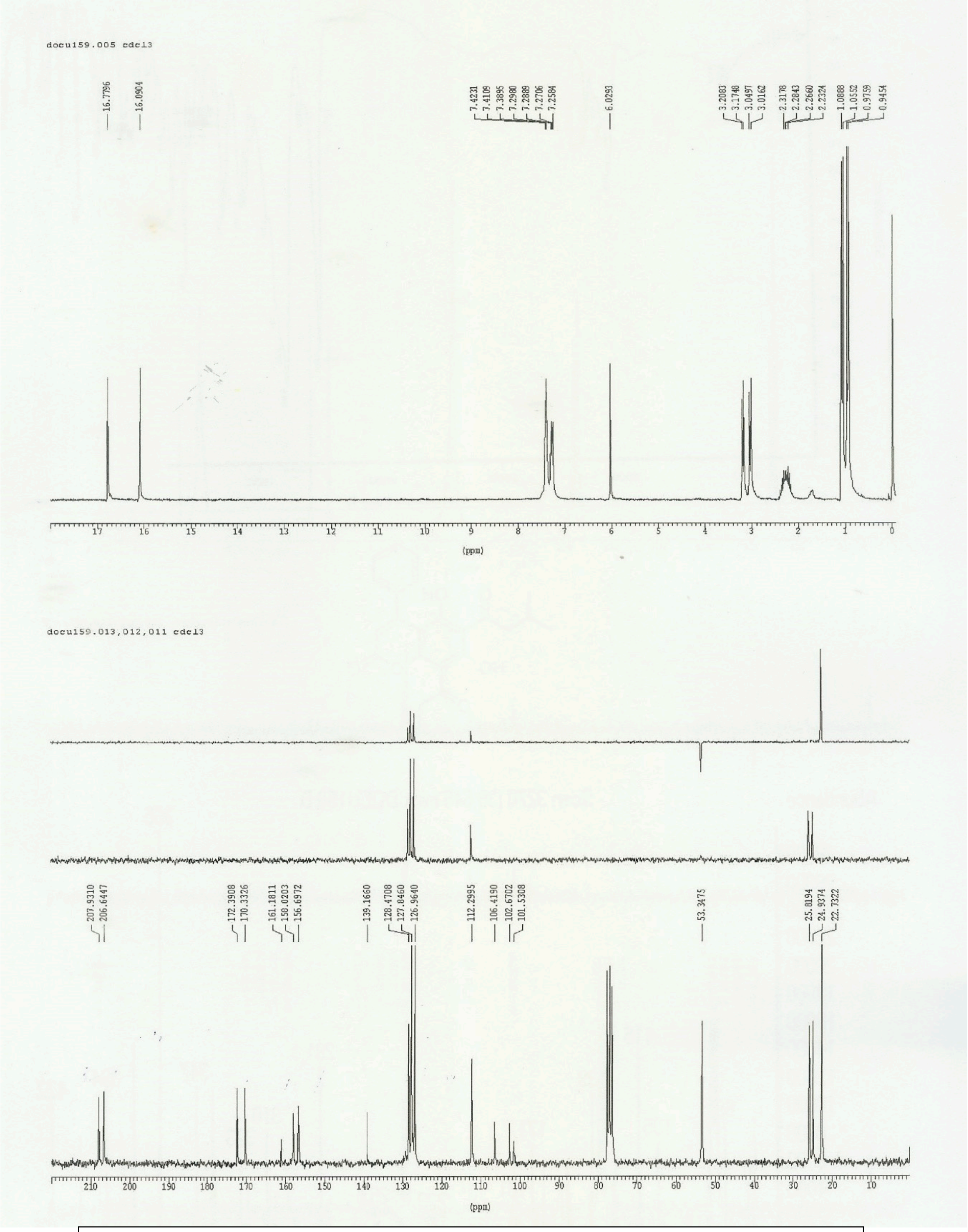

5,7-dihydroxy-6,8-bis(3-methylbutanoyl)-4-phenyl-2H-chromen-2-one (**19**)

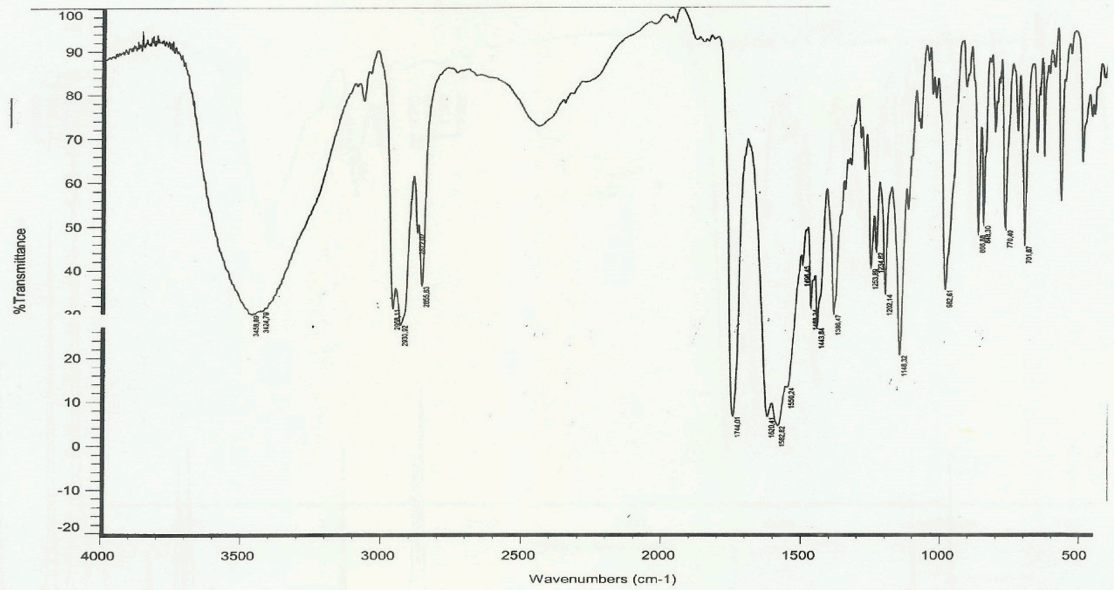

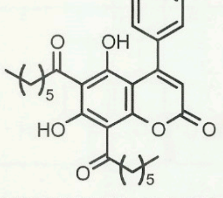

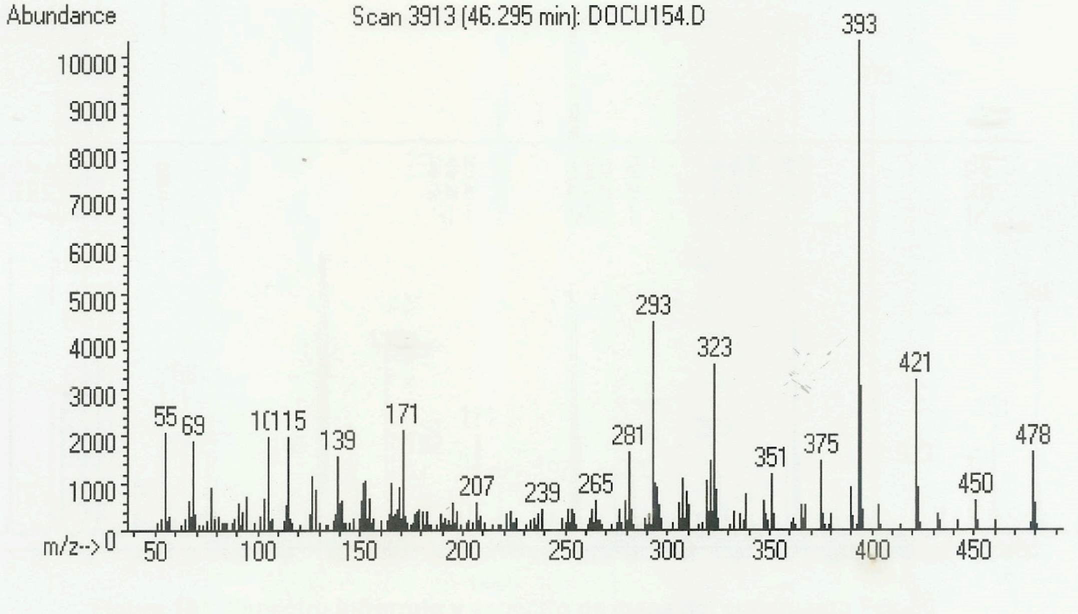

6,8-diheptanoyl-5,7-dihydroxy-4-phenyl-2H-chromen-2-one (**20**)

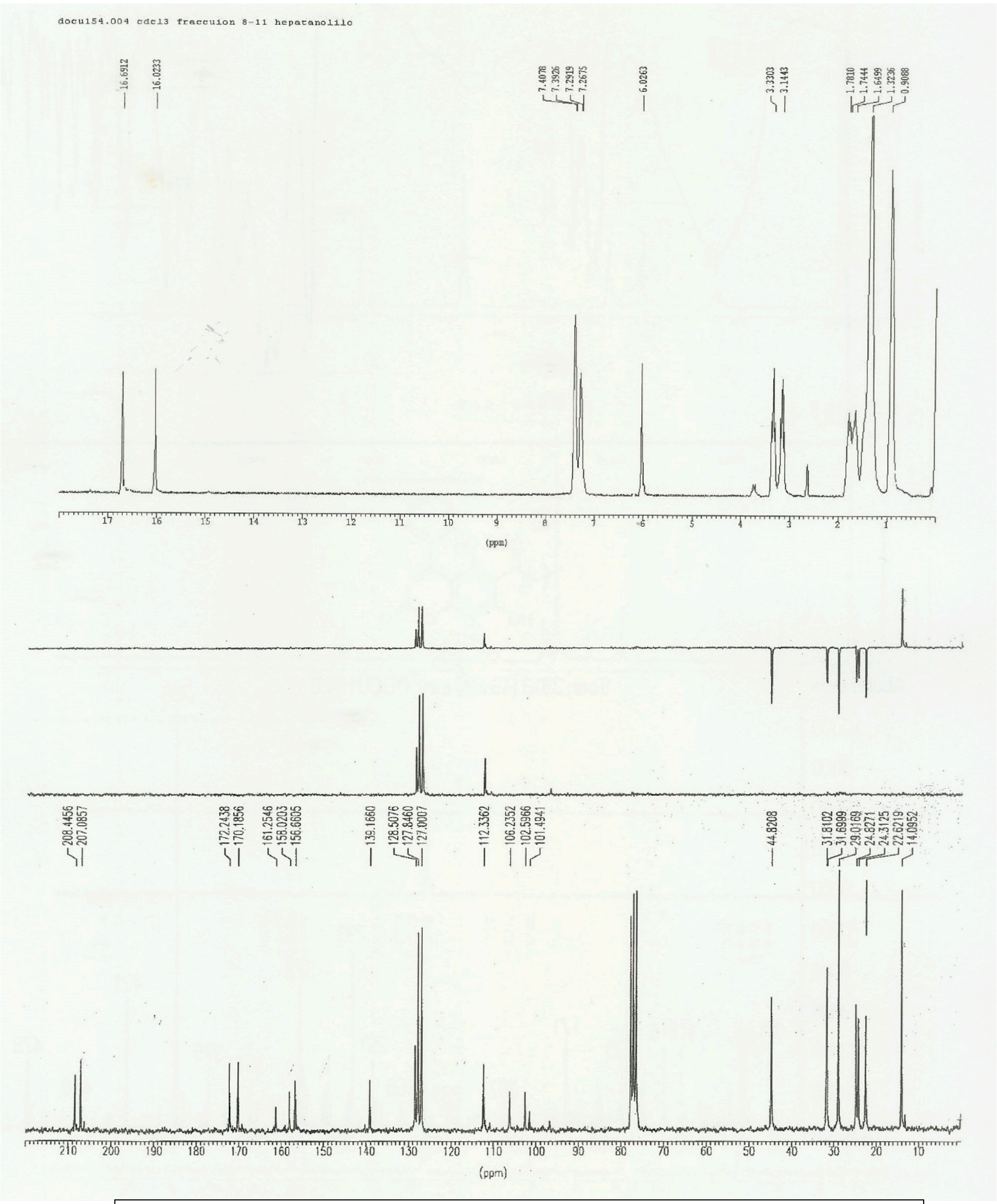

6,8-diheptanoyl-5,7-dihydroxy-4-phenyl-2H-chromen-2-one (**20**)

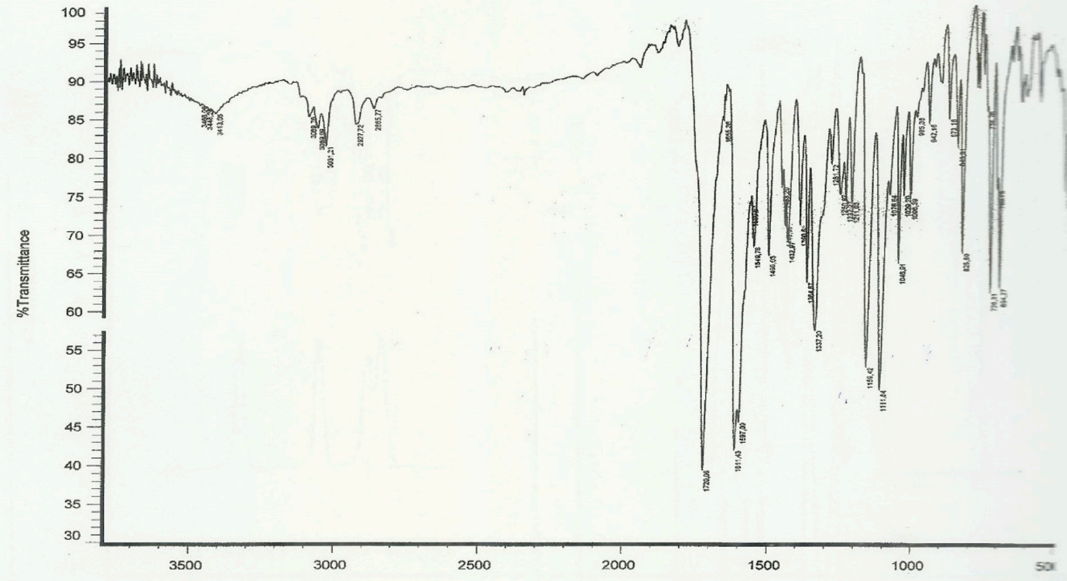

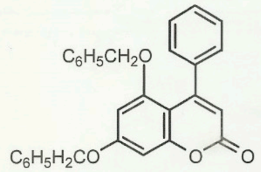

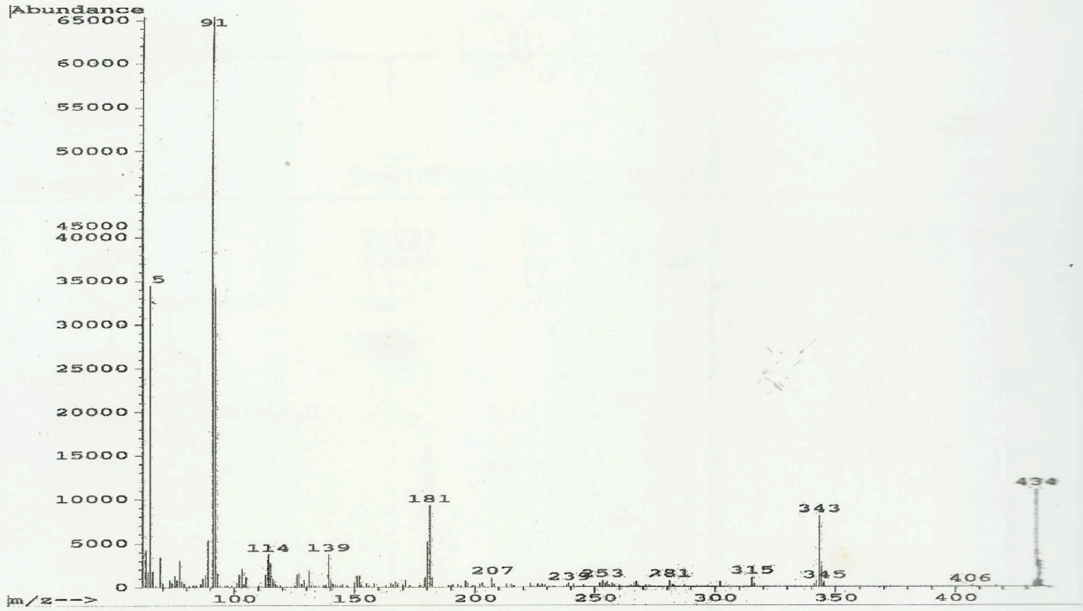

5,7-dibenzyloxy-4-phenyl-2H-chromen-2-one (**21**)

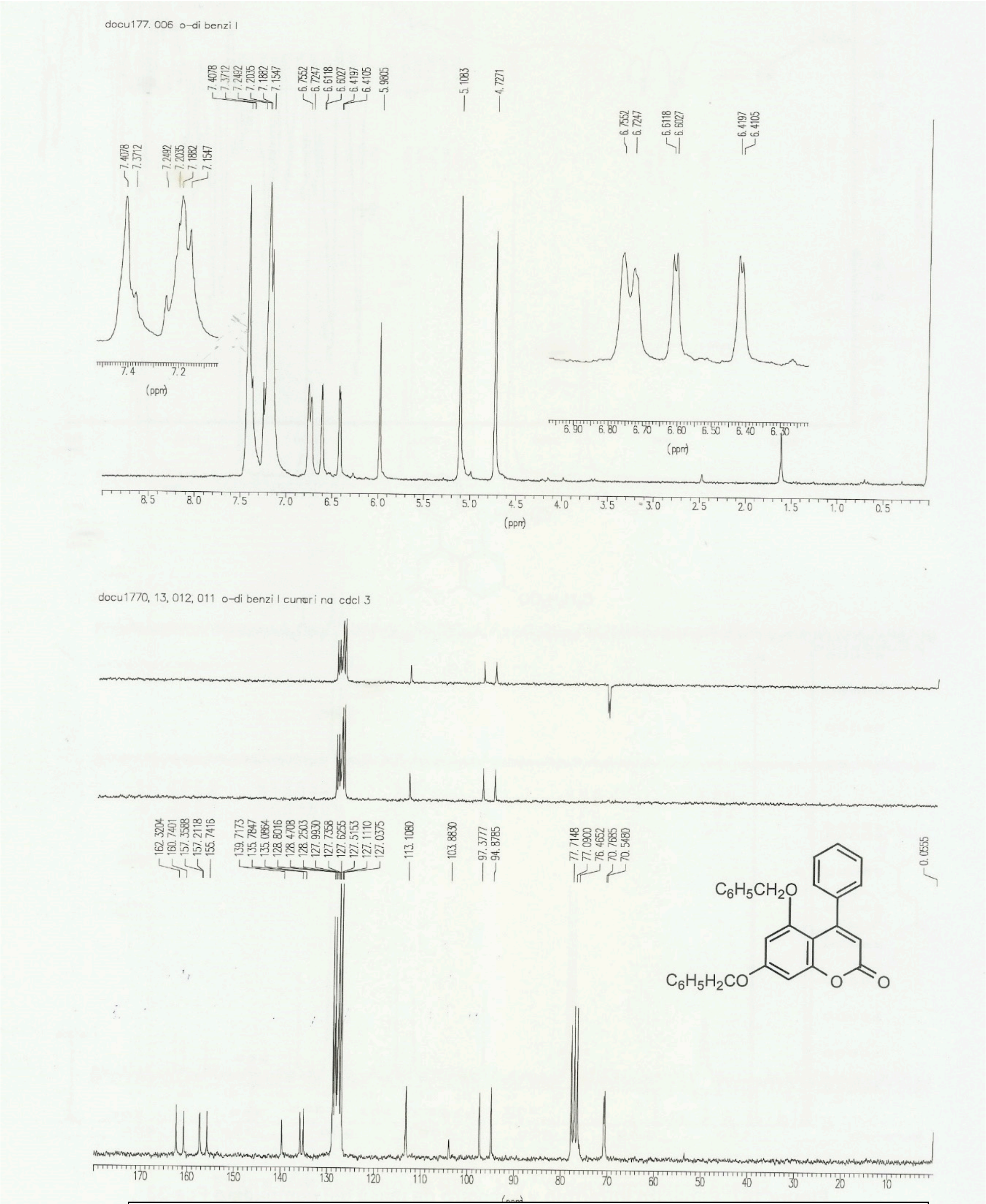

5,7-dibenzyloxy-4-phenyl-2H-chromen-2-one (**21**)

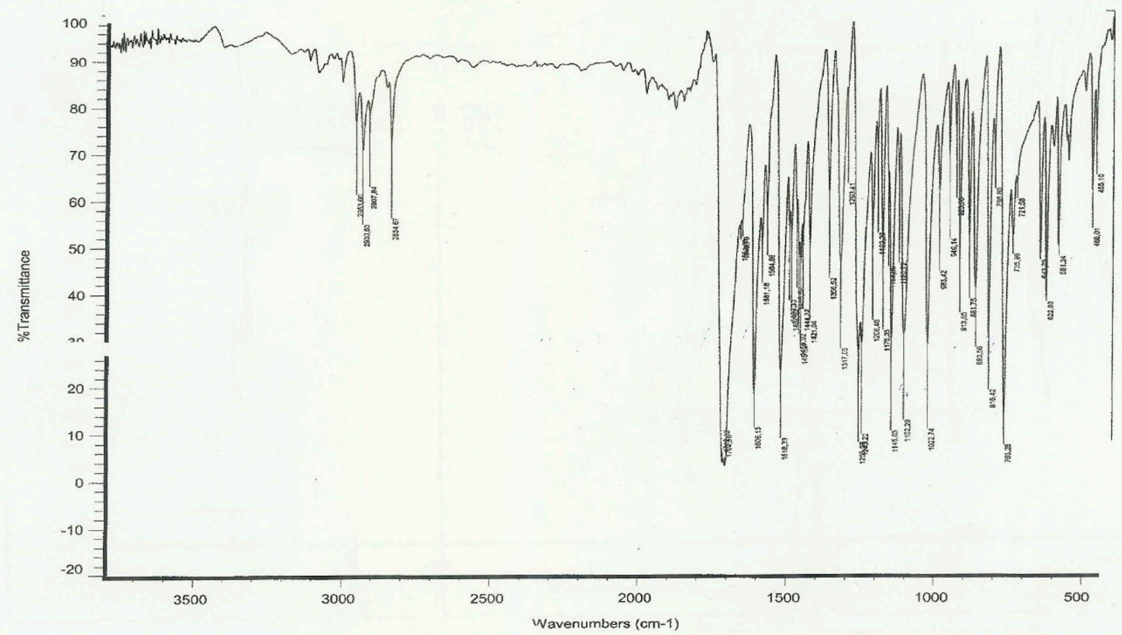

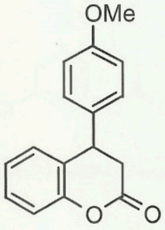

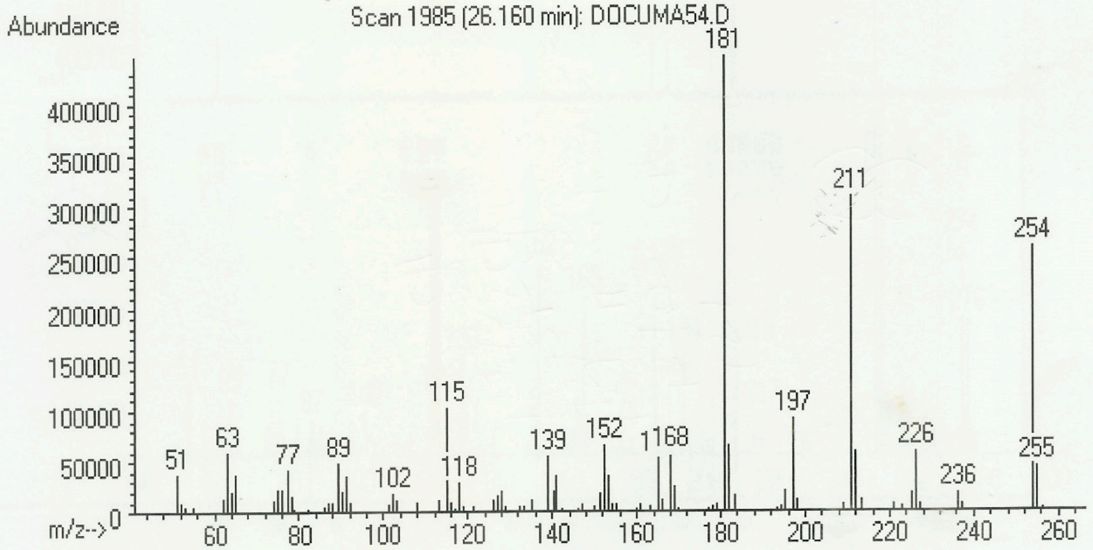

4-(4-methoxyphenyl)chroman-2-one (**22**)

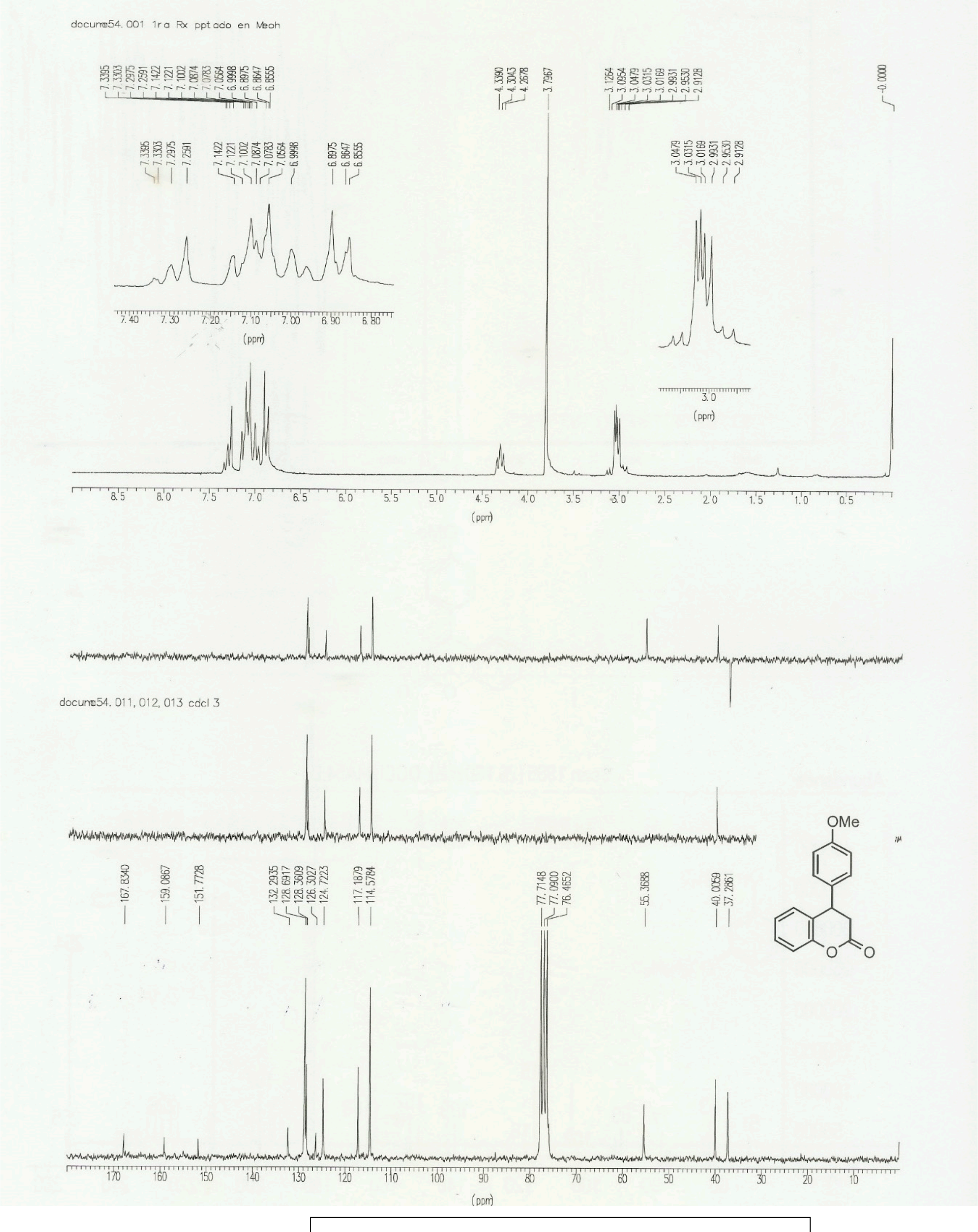

4-(4-methoxyphenyl)chroman-2-one (**22**)

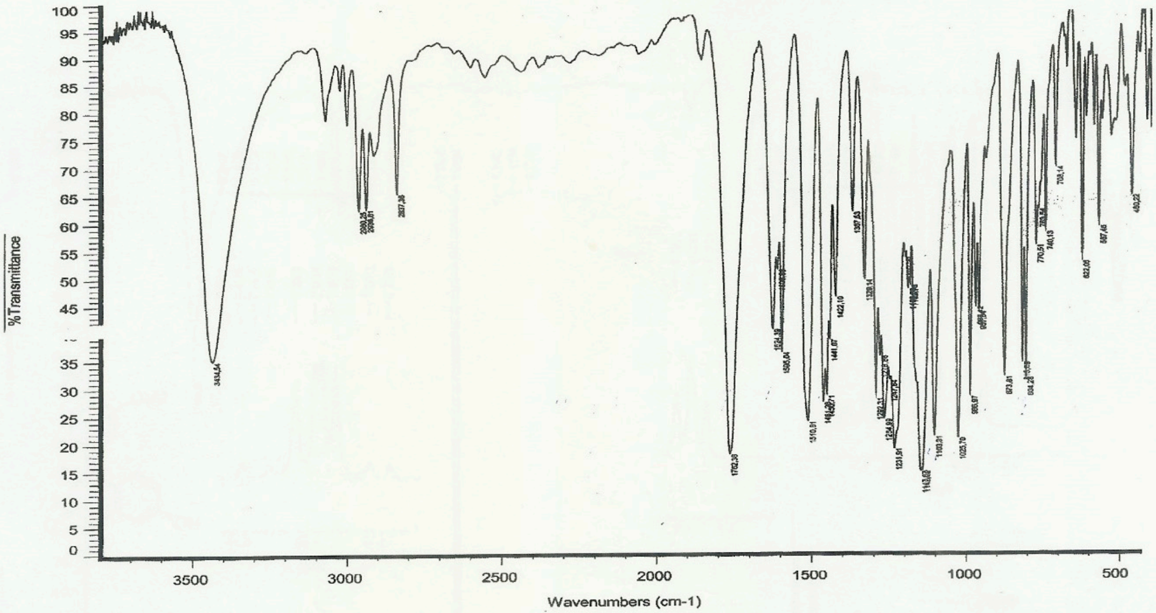

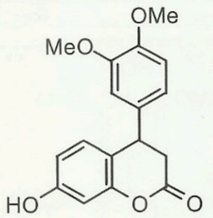

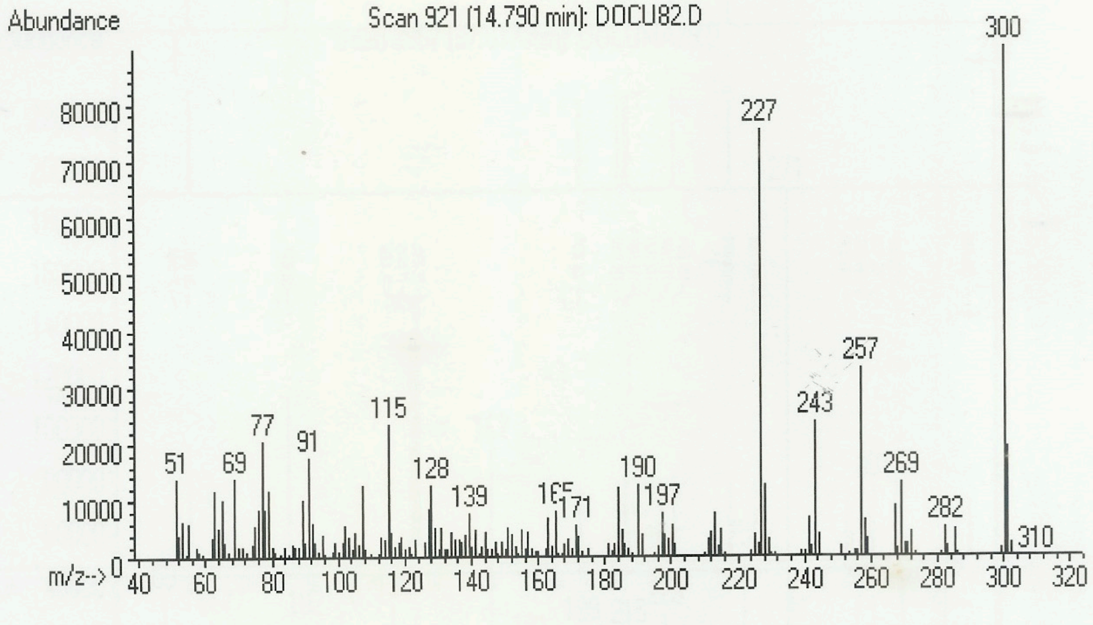

4-(3,4-dimethoxyphenyl)-7-hydroxychroman-2-one (**23**)

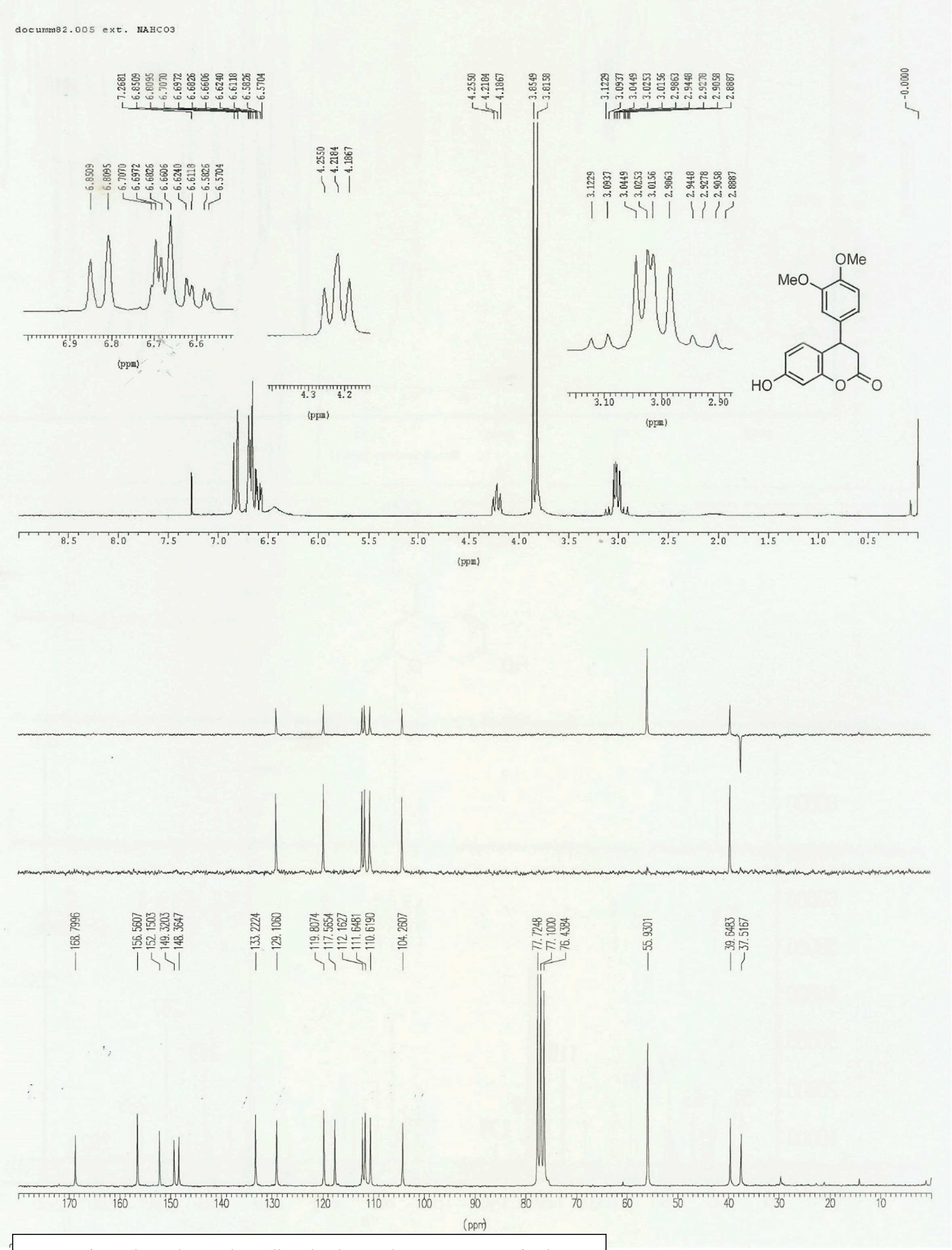

4-(3,4-dimethoxyphenyl)-7-hydroxychroman-2-one (**23**)

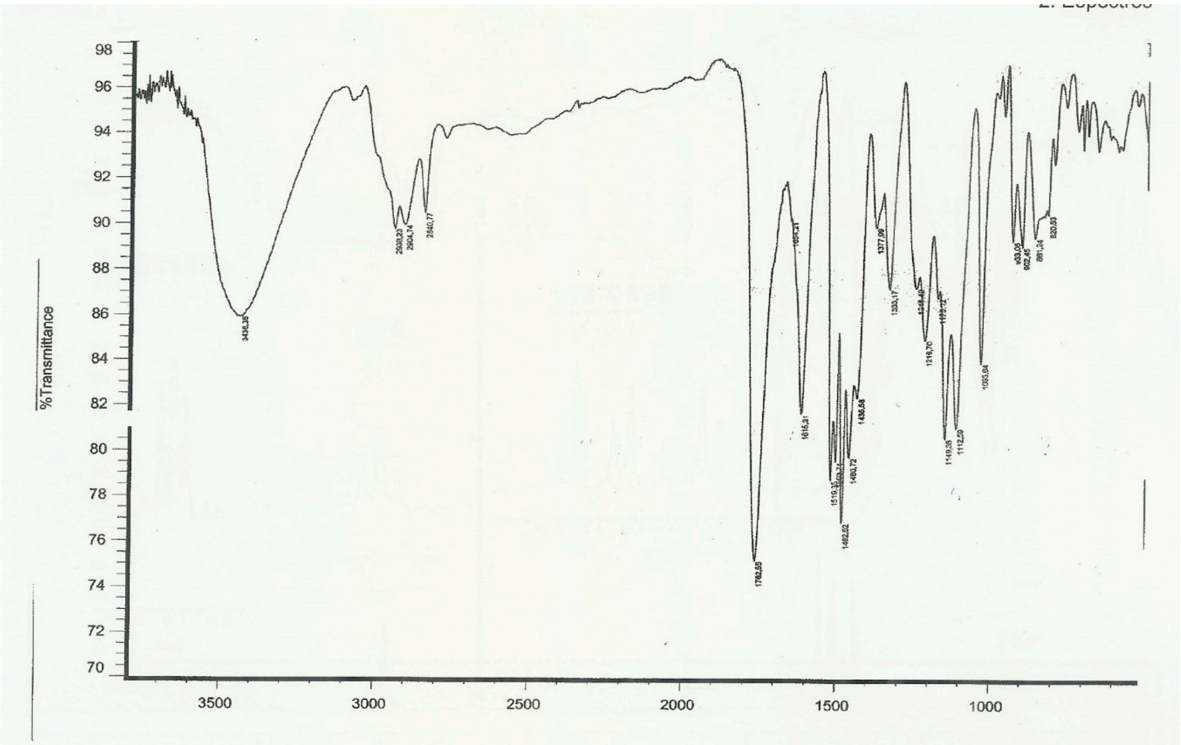

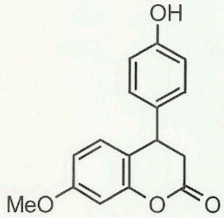

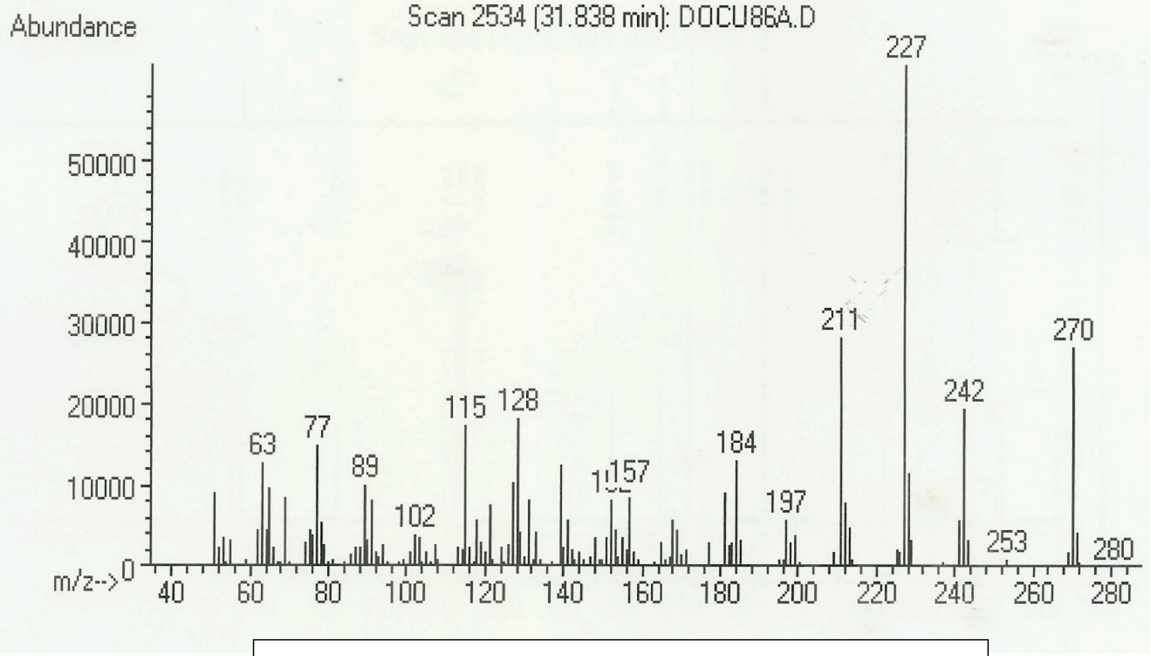

4-(4-hydroxyphenyl)-7-methoxychroman-2-one (**24**)

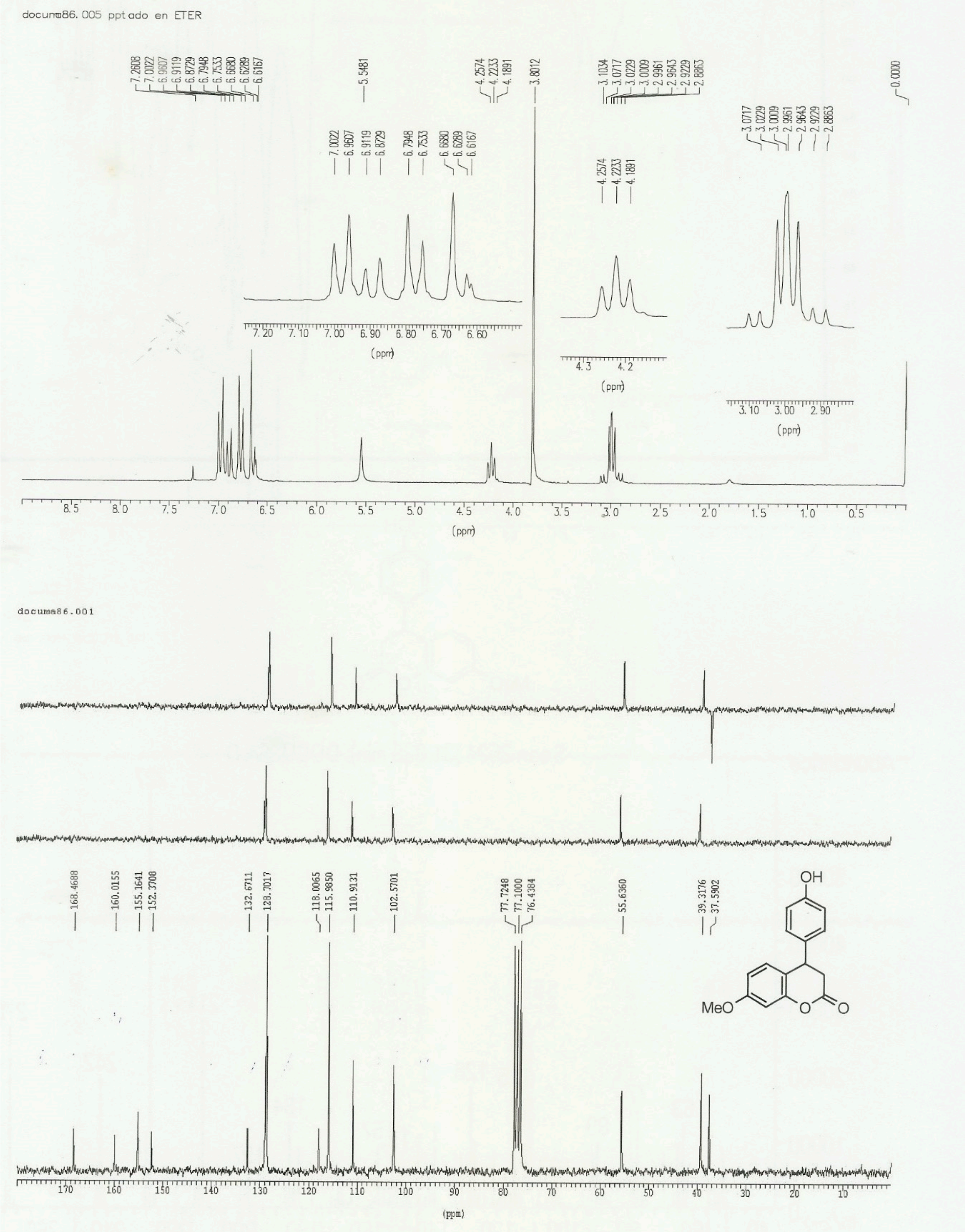

4-(4-hydroxyphenyl)-7-methoxychroman-2-one (**24**)

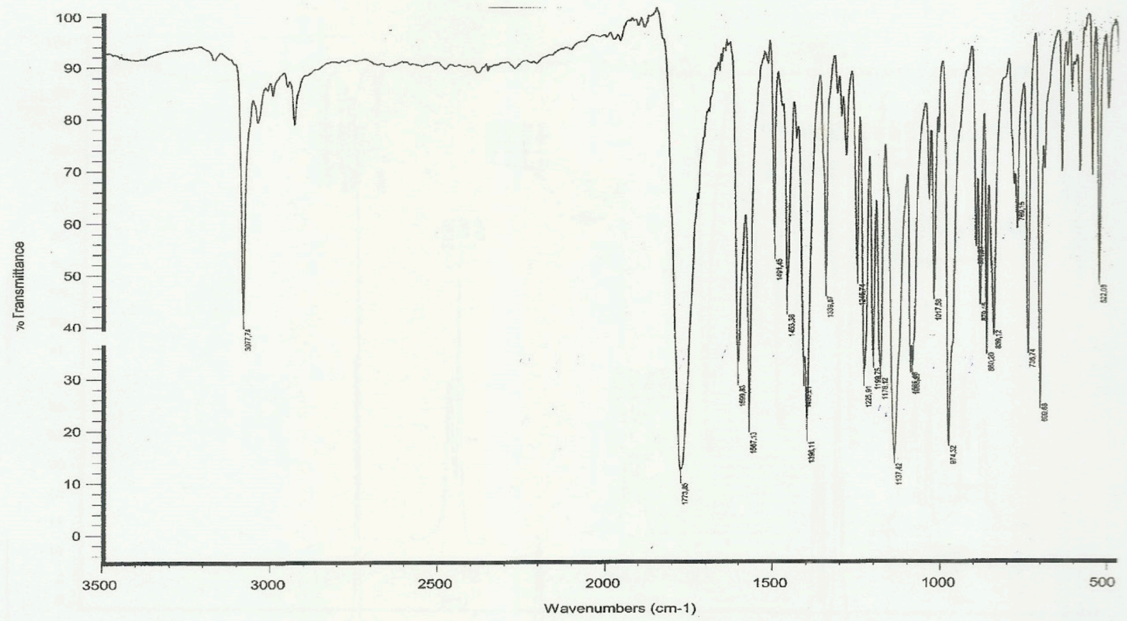

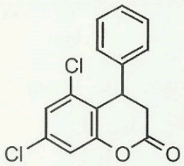

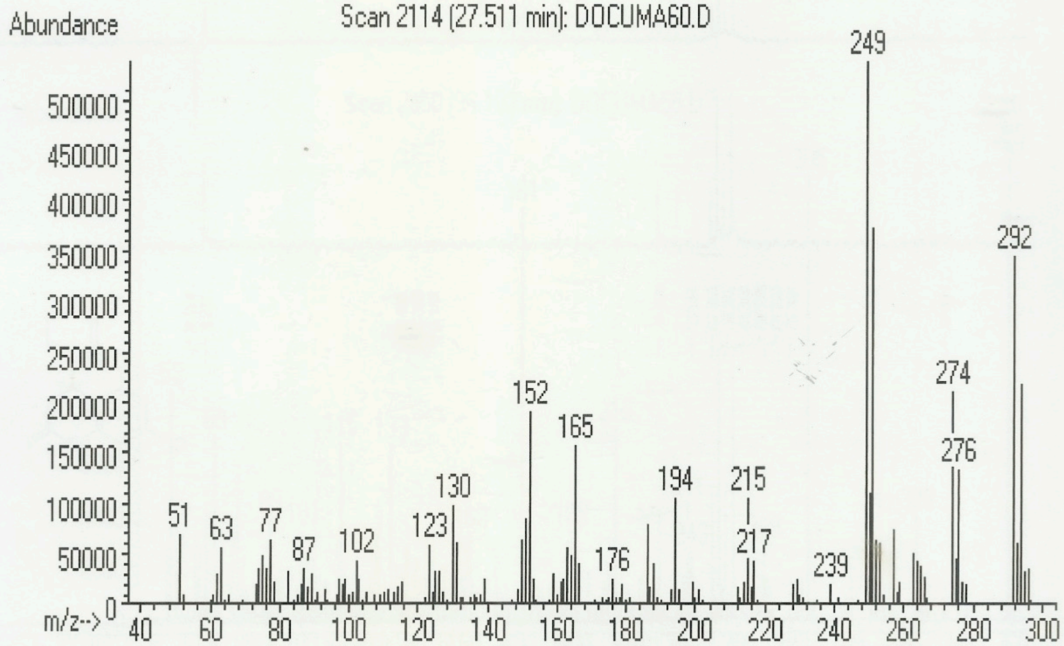

5,7-dichloro-4-phenylchroman-2-one (**25**)

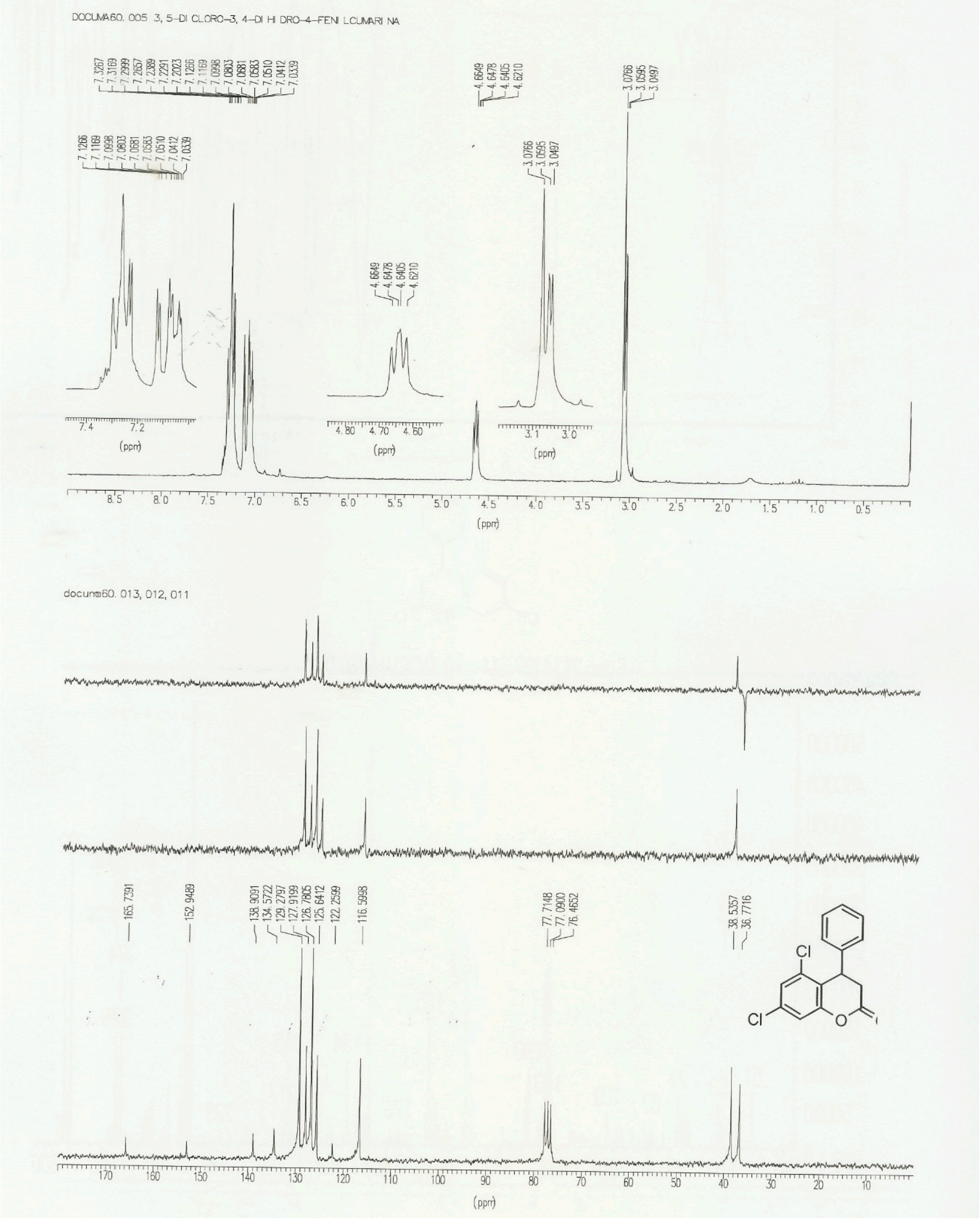

5,7-dichloro-4-phenylchroman-2-one (**25**)

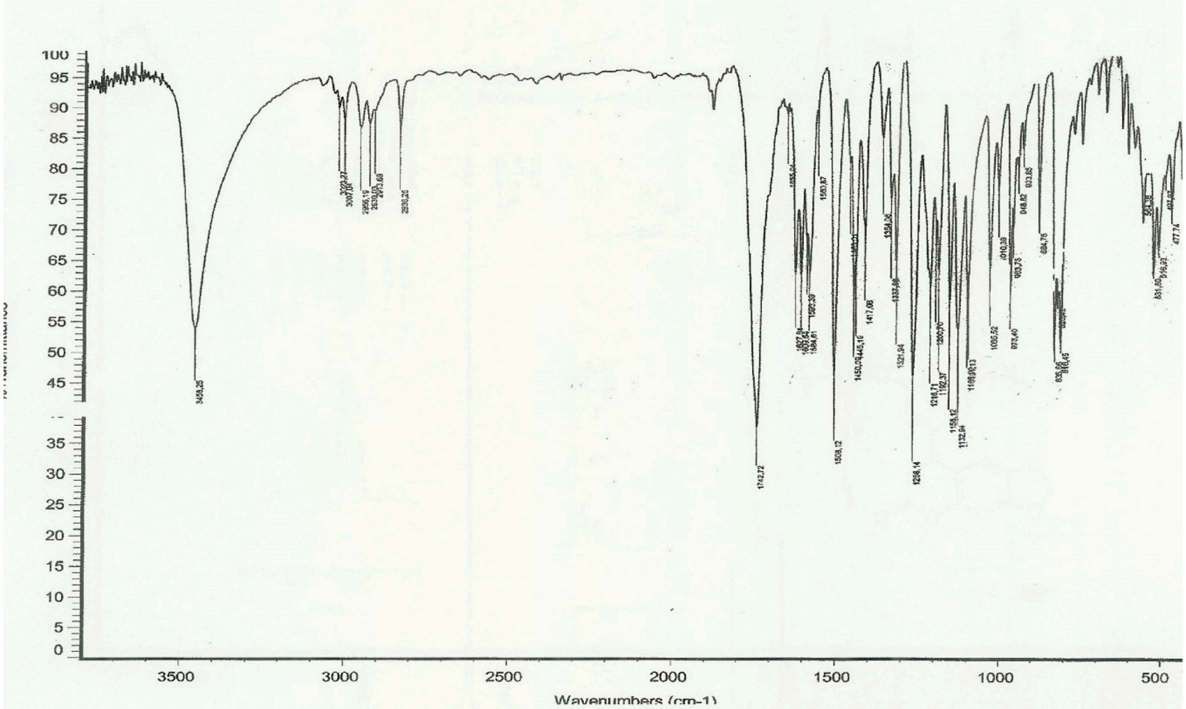

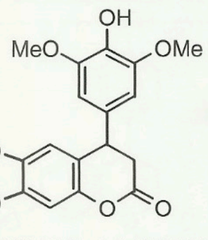

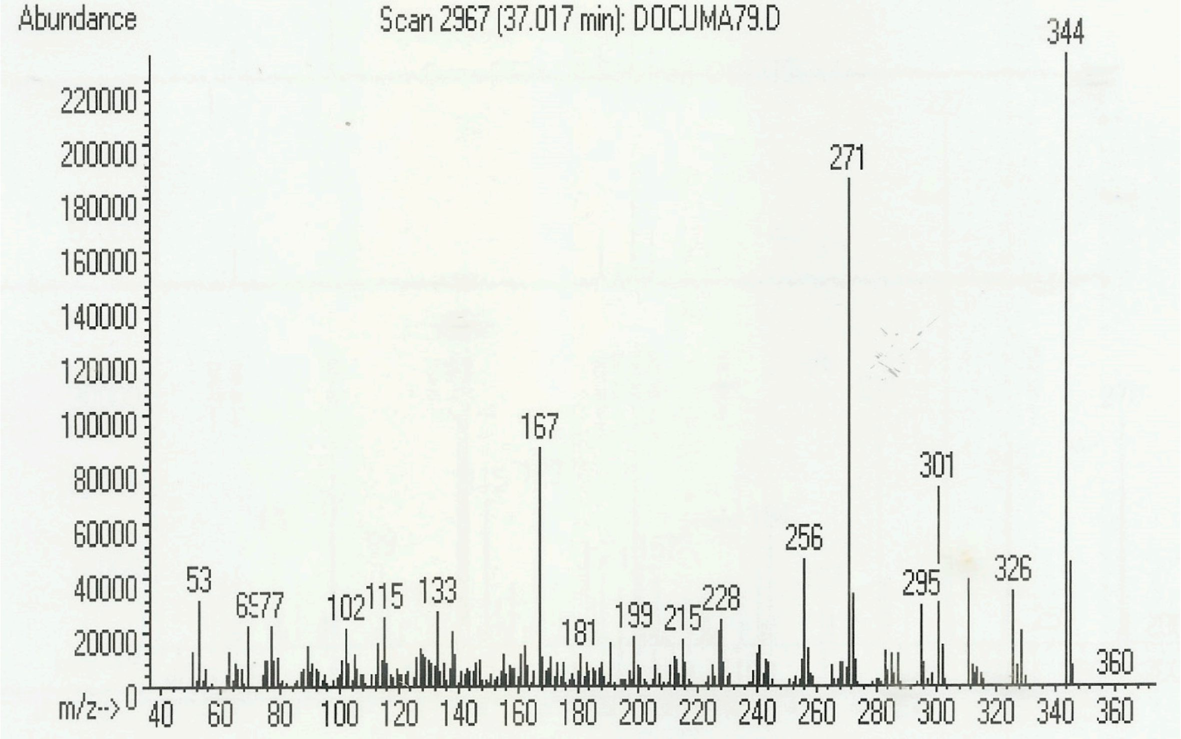

8-(4-hydroxy-3,5-dimethoxyphenyl)-7,8-dihydro-[1,3]dioxolo[4,5-g]chromen-6-one (**26**)

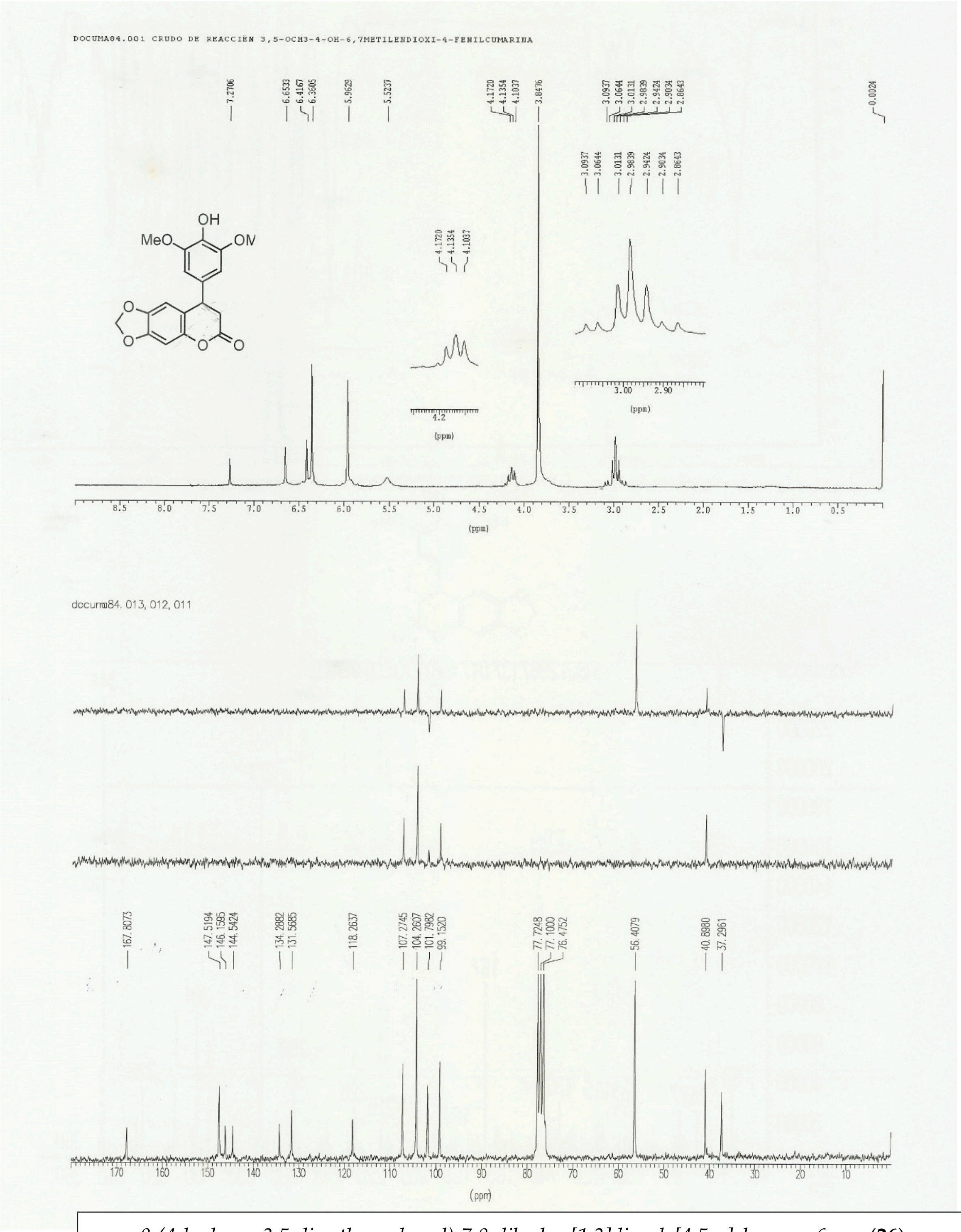

8-(4-hydroxy-3,5-dimethoxyphenyl)-7,8-dihydro-[1,3]dioxolo[4,5-g]chromen-6-one (**26**)

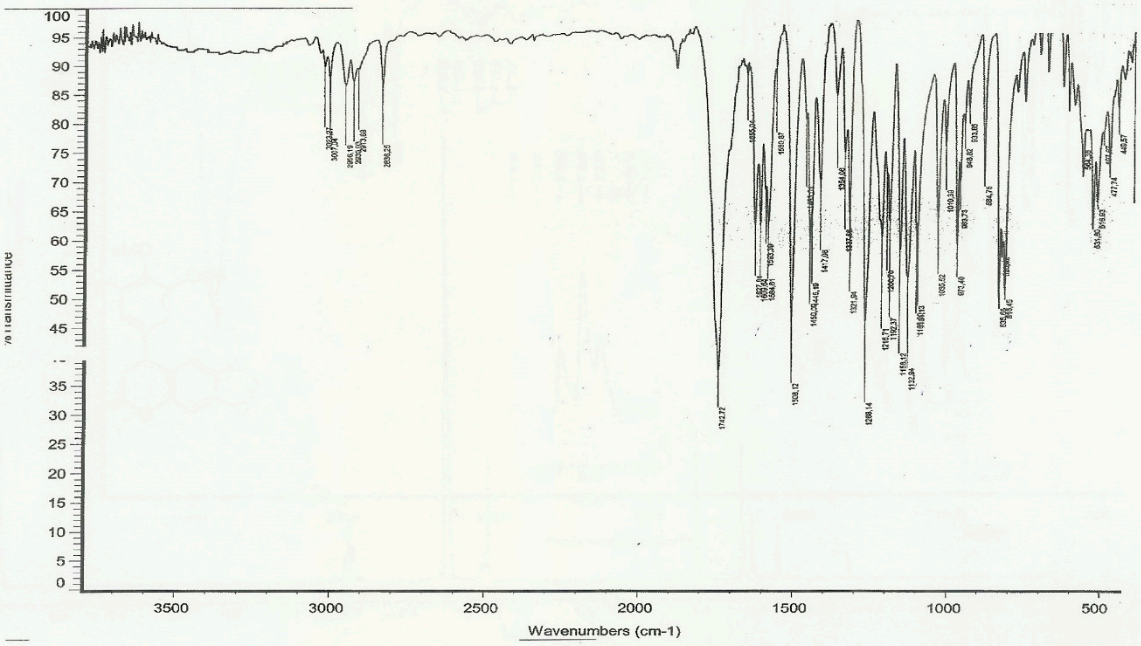

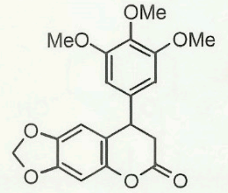

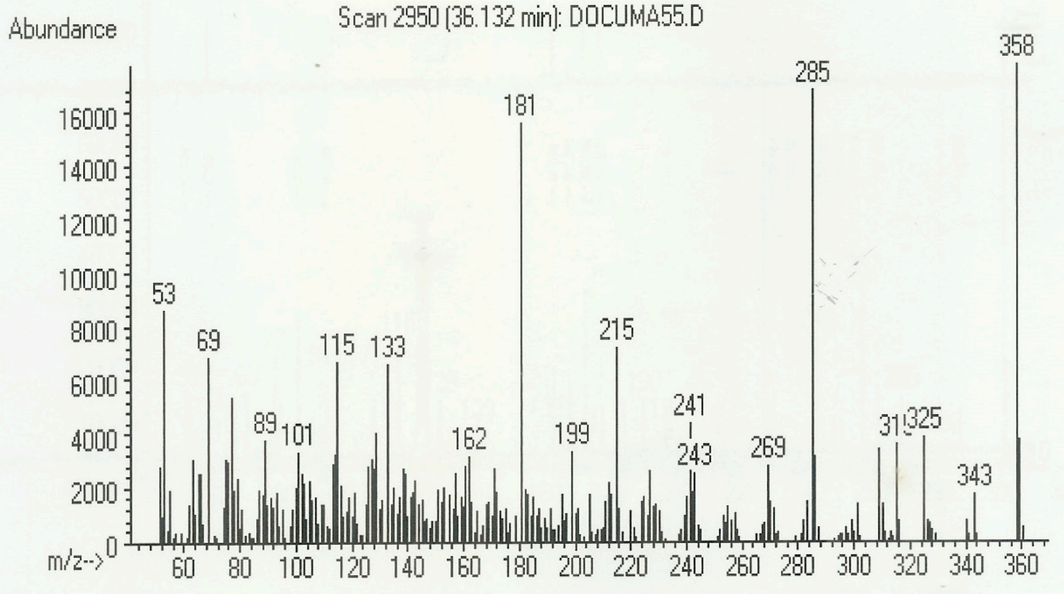

8-(3,4,5-trimethoxyphenyl)-7,8-dihydro-[1,3]dioxolo[4,5-g]chromen-6-one (**27**)

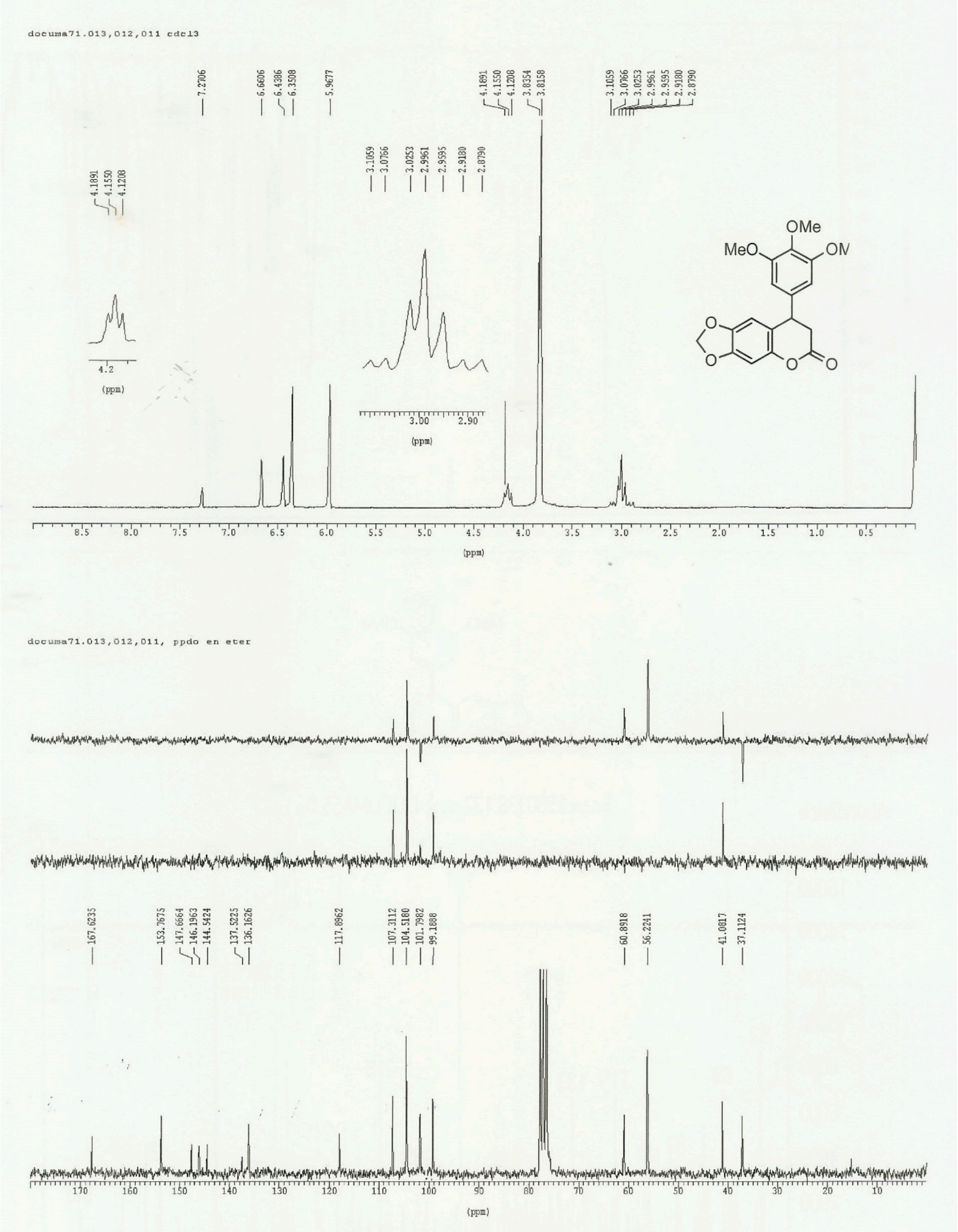

8-(3,4,5-trimethoxyphenyl)-7,8-dihydro-[1,3]dioxolo[4,5-g]chromen-6-one (**27**)

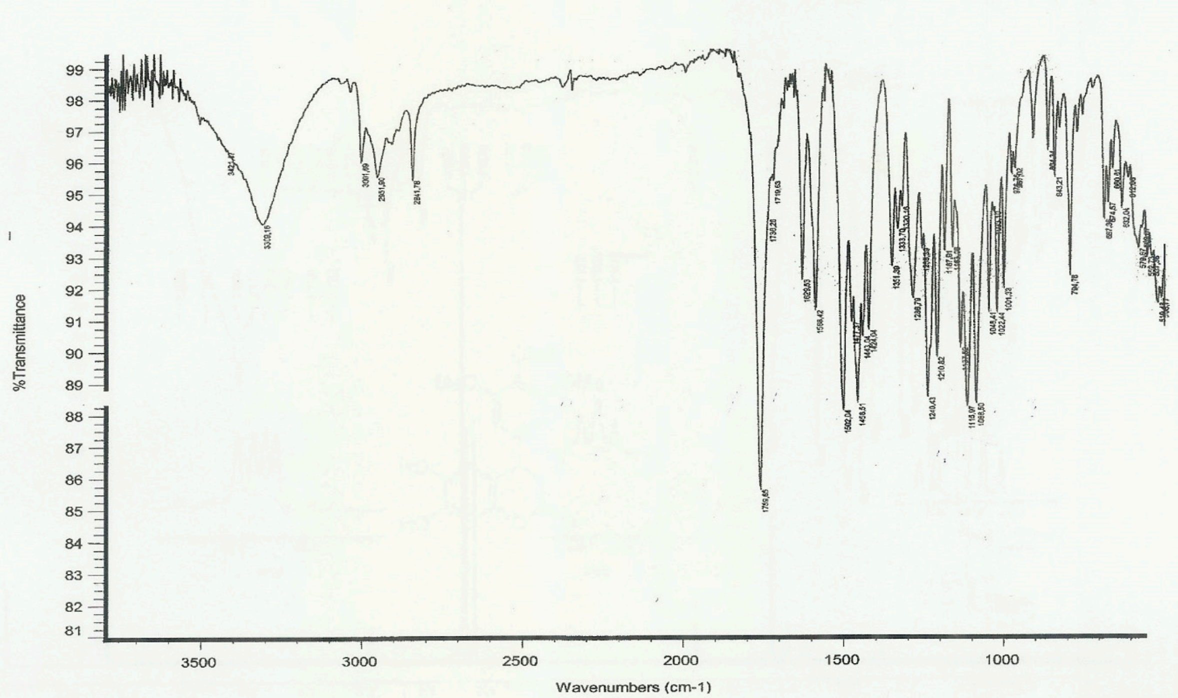

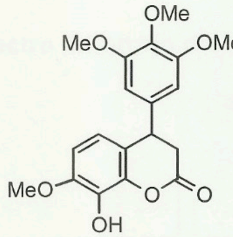

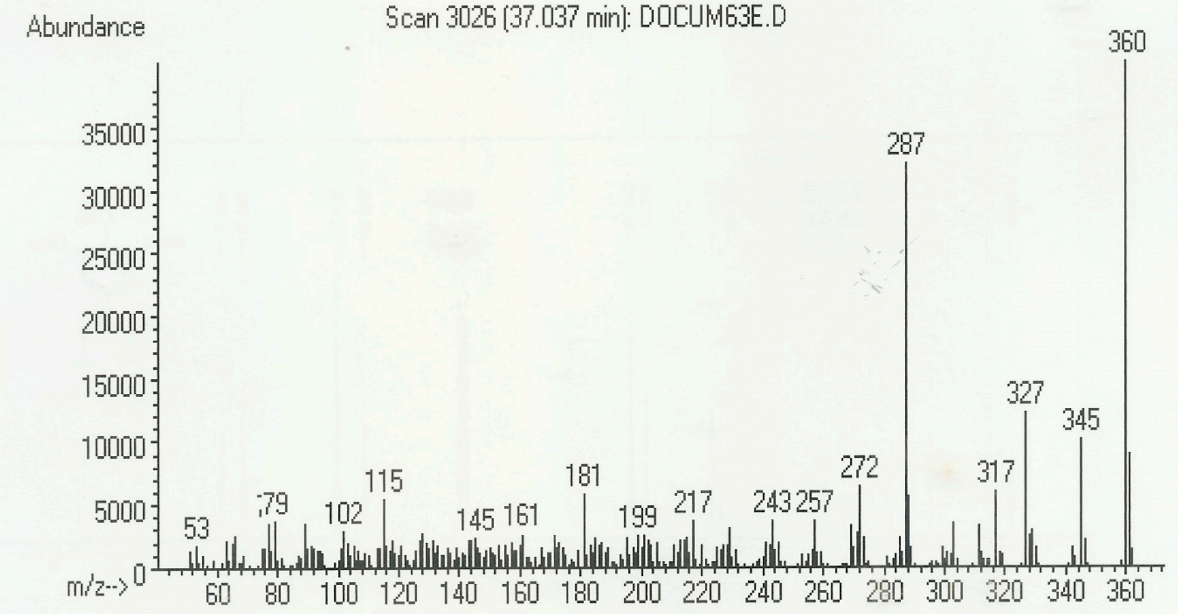

8-hydroxy-7-methoxy-4-(3,4,5-trimethoxyphenyl)chroman-2-one (**28**)

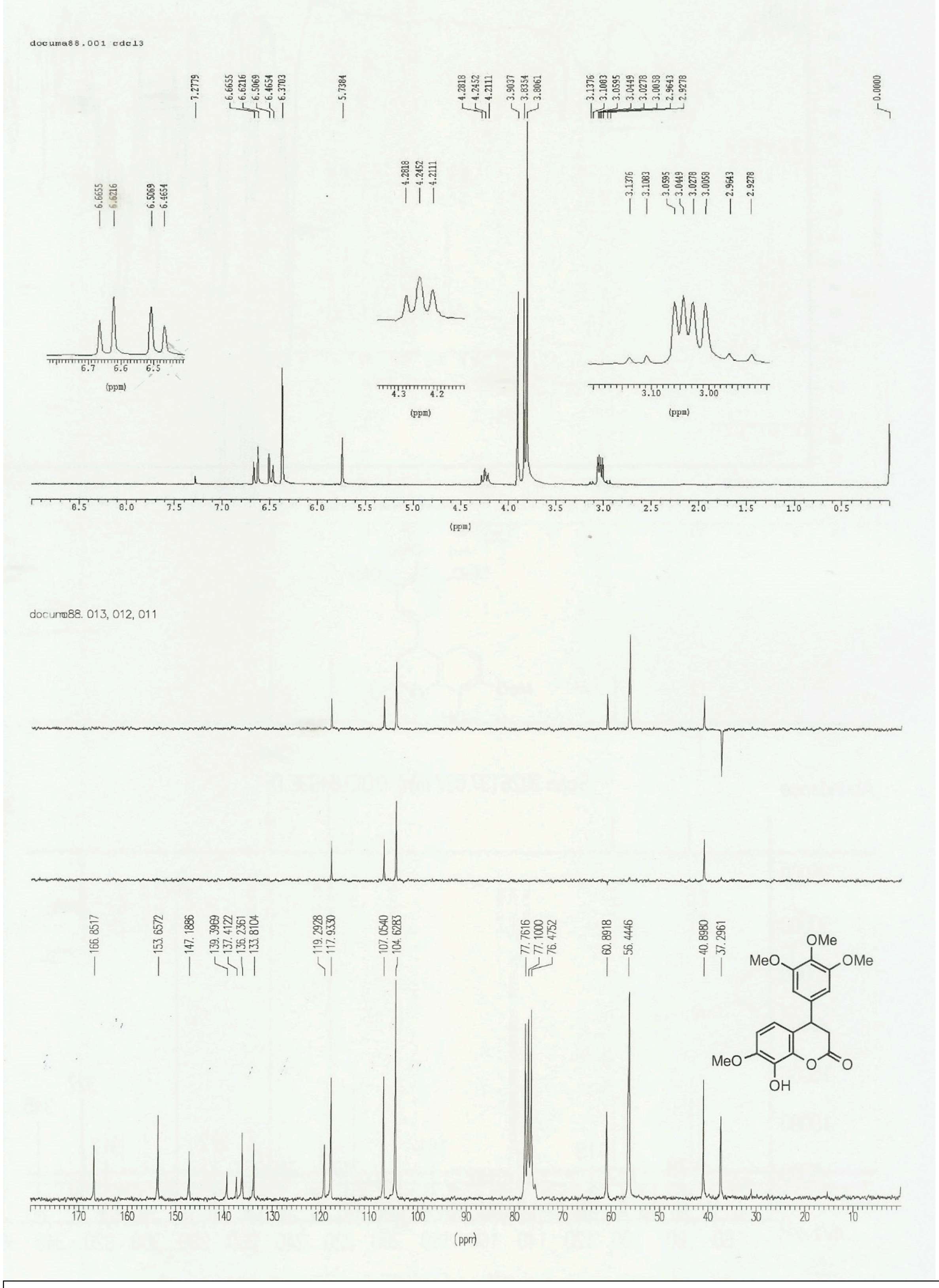

8-hydroxy-7-methoxy-4-(3,4,5-trimethoxyphenyl)chroman-2-one (**28**)
